# Application of andesite and hydrolyzed poly acrylonitrile andesite composite for adsorption of Al(III), Fe(III), CH_3_SH, and H_2_S form aqueous solutions

**DOI:** 10.1038/s41598-025-09497-8

**Published:** 2025-07-20

**Authors:** Abdalla M. Khedr, Nadia Elwakiel, Sameh E. Halawia, Ramadan Abdelghany Mansour

**Affiliations:** 1 Chemistry Department, Faculty of Science, Tanta University, Tanta, 31527 Egypt; 2 Basic Sciences and Engineering Department, Higher Institute of Engineering and Technology, New Damietta, 34517 Egypt

**Keywords:** Adsorption, Andesite, Composite, Isotherm, Kinetic, Thermodynamic, Chemistry, Materials science

## Abstract

The hydrolyzed poly acrylonitrile andesite composite (HPAA) was prepared and characterized using BET analysis, zeta potential measurements, XRD and XPS before and after the adsorption process. Both the HPAA composite and andesite were analyzed using FTIR spectroscopy. The effect of adsorption on the surface morphology and crystallinity of andesite was evaluated using SEM imaging. To successfully extract metal ions and gas molecules Al(III), Fe(III), H_2_S, and CH_3_SH from an aqueous solution, andesite and HPAA composite were employed. This study examined the adsorption process on andesite and the HPAA composite for Al(III) and Fe(III) under the following circumstances: temperature (25–50) °C, retention time (5–90) minutes, pH (2–8), dose (0.005–0.1) g L⁻^1^, and initial concentration (0.1, 0.2, 0.4, 0.5) mg L⁻^1^; for H_2_S and CH_3_SH, dose (0.02–2) g L⁻^1^, retention time (2–25) minutes, pH (2–10), temperature (25–50) °C, and (20–100) mg L⁻^1^ H_2_S and (2–10) CH_3_SH mg L⁻^1^. All of these factors influence adsorption capacity, which increases with retention time, pH, dosage, temperature and initial concentration but adsorption efficiency% decreases as initial concentration increases. For Al(III) and Fe(III), the ideal numbers for pH, retention time, ion concentration, dosage, and temperature were 6.00, 30 min., 0.5 mg L⁻^1^, 0.025 g L⁻^1^, and 25 °C, accordingly; for H_2_S and CH_3_SH, they were 9.00, 10 min. (100 mg L⁻^1^ for H_2_S and 10 mg L⁻^1^ for CH_3_SH), 1.00 g L⁻^1^, and 25 °C. The PAA composite was prepared using the bulk technique, while the HPAA composite was prepared using hydrolyzed. The maximal adsorption capacity and adsorption efficiency% for Al(III) and Fe(III) on andesite were (17.35, 15.39) mg g⁻^1^ and (96.00, 94.08), respectively; when utilizing HPAA, was (18.15, 17.79) mg g⁻^1^ and (100.00, 97.58). The highest adsorption capacity and adsorption efficiency% for H_2_S and CH_3_SH on andesite were (94.48, 9.08) mg g⁻^1^ and (97.50, 95.00), respectively, while the HPAA was (98.40, 9.75) mg g⁻^1^ and (100.00, 99.00). The assessment of thermodynamic parameters, such as ΔH, ΔG, and ΔS, was essential in demonstrating that the heavy metal adsorption process on andesite and HPAA was endothermic, indicating that its physical characteristics enhanced with an increase in temperature. It was shown that the linear form of the Langmuir adsorption equation corresponded to the adsorption of Al(III), Fe(III), H_2_S, and CH_3_SH on andesite and HPAA. The linear version of the Freundlich and Temkin adsorption equations is satisfied by the adsorption of H2S and CH3SH on andesite and HPAA. The pseudo-second-order kinetic model better predicts the sorption of Al(III), Fe(III), H2S, and CH3SH by andesite and HPAA. The HPAA composite was applied as an adsorbent for the extraction of Al^3^⁺, Fe^3^⁺, H_2_S, CH_3_SH, Na⁺, NH₄⁺, Cl⁻, Br⁻, NO₃⁻, SO₄^2^⁻, and K⁺ from real wastewater samples.

## Introduction

Many industrial processes can produce heavy metals, including lead smelting, textile manufacturing, metal finishing, electroplating, plating, and the glass and ceramics sectors. Humans readily absorb aluminum (Al) due to its widespread use in daily life and its status as the most extensively distributed metal in the environment. This hazardous metal can be inhaled through food, drink, and the air. It can also be found in environmental, cosmetic, and medicinal items. One significant coagulant used in the purification and treatment of water is aluminum chloride (AlCl_3_). The element’s widespread distribution increases the risk of exposure to humans and harm^1–6^. Compared to the water we drink, which contains 0.2 to 0.4 mg of aluminum per day, meals and drinks include 2.5 to 13 mg. Drugs may be a factor in increased aluminum levels. Antiacids (two tablets) can give up to 500 mg of Al^7^.

The main ways that populations become vulnerable to iron are through food and drink^8^. Iron (Fe) is a basic element that practically all living things use. It is commonly included in oxygen transport proteins, including myoglobin in muscle cells and hemoglobin in red blood cells, as well as the heme complex, which mediates redox reactions. It is also present in the liver, spleen, and bone marrow and is essential for energy^9,10^ production and immune system response. Additionally, the recommended daily intake of iron changes as people age. Adults should take ten times as much (18 mg/kg/day)^11^ as toddlers up to three months old, who should take 1.7 mg/kg/day. Neurodegenerative disorders are exacerbated by iron’s detrimental effects on neural tissue. Additionally, individuals with a variety of neurological disorders, including Alzheimer’s disease, Parkinson’s disease, and stroke^12^, have observed iron deposits. Advanced neuroimaging methods and pathology investigations have shown that as people age, their brain iron levels increase.

Heavy metals have been eliminated from aqueous solutions using a variety of technologies, including the removal of Cu^2+^, Fe^3+^, Mn^2+^, Pb^2+^, and Zn^2+^ from water-based solutions under various conditions^13^, taking heavy metal ions out of wastewater, a thorough and crucial analyze^14^, studies of the physical characteristics of geopolymers to substitute activated carbon for the elimination of heavy metals^15^, the removal of protons, aluminum Al(III), and iron Fe(II) on lepidocrocite (γ-FeOOH)^16^, and chitosan used for Al(III) adsorption^17^.

CH_3_SH is a colorless, ephemeral, and unstable organic sulfur molecule, whereas methyl mercaptan (methanethiol) is esteemed for its pungent odor. Numerous chemical and agricultural processes can release it, which is abundant in petroleum, coal, and natural gas. By altering the Earth’s radiative equilibrium and contributing to the production of sulfate particles in the atmosphere, CH_3_SH can cause acid rain. It can lead to issues in the chemical industry and harm the environment. Because of its potent adsorption onto metal or metal oxide surfaces, sulfur is a renowned toxin for many industrial catalysts. Current bimetallic reforming catalysts, for instance, have significantly decreased catalytic activity when as little as 1.00 ppm sulfur is present. Sulfur also contributes to reactor and infrastructure corrosion. Thus, it is crucial to remove CH_3_SH from input gas^18^.

With considerable volatility (Henry’s law coefficient of 0.2 mol/L at 25 °C) and a low odor level (0.9–8.5 ppb), CH_3_SH is a distinctive volatile sulfur component in LFG. CH_3_SH’s toxicity and disagreeable smell have earned it a bad reputation. Interaction with cytochrome C oxidase can decrease liver mitochondrial respiration. However, it can also damage membranes by binding directly to the erythrocyte membrane^19^ or blocking enzymes that guard against oxidative damage. Nitrogen-rich coconut shell-activated carbon^19^ prevented CH_3_SH.

Hydrogen sulfide (H_2_S) is a major issue in most energy businesses, including fuel cells, natural gas, liquefied petroleum gas, tail gas, and transportation gases like jet fuel, petrol, and diesel. It should be eliminated from the environment since it is a hazardous material that creates acid rain when oxidizing to SO_2_. The typical technique for treating H_2_S involves adsorption, washing, and low-temperature biological therapy (298–373 K)^20^.

The dry adsorption method is cost-effective^21^, environmentally friendly, and readily available. Several techniques and adsorbent types have been used, including metal oxides, activated carbons, and zeolites^22^. We evaluated the optimal conditions for removing Al(III), Fe(III), H_2_S, and CH_3_SH from an aqueous solution. Table 1 displays the highest heavy metal ion adsorption efficiency for an appropriate adsorbent.

**Table 1:** Analysis of the maximum adsorption capacities of various adsorbents.

Heavy metals	Adsorbents	Adsorption capacity (mg g⁻^1^)
Al^3+^	Dye-affinity^23^	17.50
	Native and oxidized starches^24^	28.74
	Precursor pistachio shells^25^	12.77
	Date-pit^26^	5.83
	BDH activated carbon^26^	6.56
	R. opacus^27^	41.58
	RHAC^28^	34.48
Fe^3+^	RHAC ^28^	45.45
	C. vulgaris^28^	24.49
	R. arrhizus^29^	34.73
	Raw clinoptilolite^30^	98.00
	Geobacillus thermodenitrificans^31^	79.90
H_2_S	Activated carbon fibers^32^	4.57
	Impregnated coconut shell activated carbon^33^	20.30
CH_3_SH	Activated carbon containing nitrogen^34^	155.00
Al^3+^	(Andesite) ^Present study^	17.35
Fe^3+^		15.39
H_2_S		94.48
CH_3_SH		9.08
Al^3+^	(HPAA) ^Present study^	18.15
Fe^3+^		17.79
H_2_S		98.40
CH_3_SH		9.75

The World Health Organization (WHO) and the United States Environmental Protection Agency (US-EPA), have set regulations regarding the permissible levels of certain heavy metals in water for human consumption, as shown in Table 2 ref.^35^.

**Table 2:** Limits of heavy metals in wastewater, as recommended by WHO, USA (EPA).

Parameter (mg L⁻^1^)	WHO	US-EPA
Hg^2+^	0.001	0.00003
Cd^2+^	0.003	0.01
Pb^2+^	0.01	0.006
Cr^6+^	0.05	0.05
Fe^3+^	5.0	NA
Al^3+^	5.0	NA

Andesite stone’s enormous size, hardness, and mechanical properties make it a popular choice for roadbed construction. Its exceptional resistance to heat and frost has led to its usage in architectural art and the building of statues and monuments since ancient times. The adsorption and bioactive properties of Wheat Rice Stone (WRS), a porphyritic andesite with a vesicular or porous structure, have led to its usage in water treatment because of cation dissociation. Ammonia-rich swine manure was treated in an aerobic bioreactor built on a modified WRS bed. In agriculture, WRS was used to improve soil fertility and lessen the negative environmental consequences of the widespread use of highly soluble fertilizers^36^.

The study seeks to assess the use of andesite and HPAA composite as a cost-effective adsorbent, focusing on its adsorption efficacy and its correlation with specific contaminants in wastewater, including Al(III) and Fe(III) metal ions, as well as the gases H_2_S and CH_3_SH, along with their negative effects on public health. The impacts of temperature, pH, adsorbent dose, initial concentration, retention time, thermodynamics, kinetics, and isotherm studies upon the extraction of Al(III), Fe(III), H_2_S, and CH_3_SH from andesite and HPAA composite are all meticulously examined. In this study, andesite has been changed to produce a hydrolysis polyacrylonitrile andesite HPAA composite with enhanced surface area and, consequently, adsorption capacity. This is in line with previous studies where modifications were performed to increase specific surface area^37^. This indicates that andesite may serve as an effective adsorbent soon, potentially influencing extensive uses and wastewater treatment technologies.

## Experimental section

### Materials and methods

#### Andesite

According to a prior study, andesite can remove significant amounts of Hg(II) as well as some cations, anions, and gases from aqueous solutions^38^. It was gathered as a raw material in Egypt and processed. Subsequent to a 12-h soaking period, the material was washed with deionized water, dried for 24 h at 60 °C, sieved through a 200 μm screen, and kept inside a borosilicate container^38^.1

#### Preparation of (poly AN-Andesite) composite PAA

**Fig. 1:**
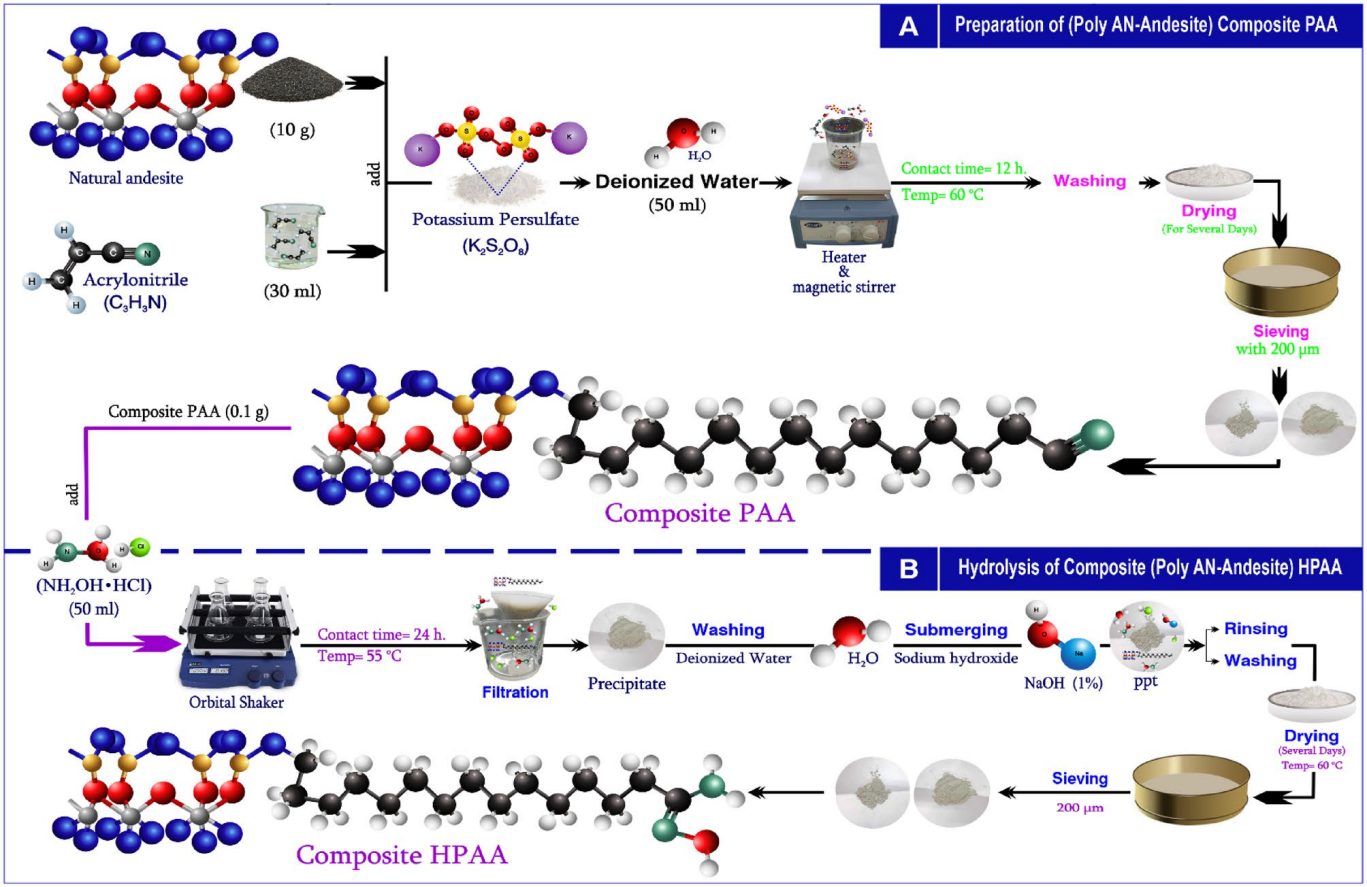
Preparation of (poly AN-Andesite) composite PAA (**A**) and composite HPAA (**B**).

Figure 1A shows, the bulk approach was utilized to prepare the composite. 10 g of andesite was combined with 30 ml of acrylonitrile and potassium persulfate, followed by the addition of 50 ml of deionized water. The resultant mixture was heated to 60 °C for 12 h. After rinsing with deionized water and drying for a four days at 60 °C, the precipitate was sieved using a 200 µm mesh screen. The chemical processes involved in the production of PAA are depicted in Eq. (1).

#### Hydrolyzed of composite (poly AN-Andesite) HPAA

0.1 g of PAA composite has been combined with 50 ml of hydroxylamine hydrochloride and shaken for 24 h at 55 °C. After rinsing with deionized water and drying for a few days at 60 °C, the precipitate was sieved using a 200 µm mesh screen. Filtration was required to obtain the PPT, which was rinsed with deionized water and submerged in a 1% solution of NaOH. Following filtering, the ppt was rinsed with deionized water, dried for several days at 60 °C, then sieved through a 200 µm mesh. The HPAA composite preparation process is illustrated in Fig. 1B. The following chemical processes occurred during the HPAA composite’s preparation:2

#### Reagents

All standards were acquired from inorganic enterprises, certified standard solution (CRM) for metals Al and Fe with concentrations of 1000 ± 3 µg/mL, indicating values of 1000 ppm. To study alternate circumstances and ultimately achieve the optimal conditions, prepare the concentration for the objective. Quality control is necessary for each sample to guarantee the accuracy of the results. The intended adsorbate concentrations are 0.1, 0.2, 0.4, and 0.5 mg L⁻^1^, sequentially. After the standard preparation, any gases that might have formed were removed using an ultrasonic device. The Millipore device provided second-hand water with a conductivity of 0.05 μS/cm, which was used after distillation. We adjusted the pH readings using 0.1 M NaOH and 0.1 M HCl. Acrylonitrile, potassium persulfate, and hydroxylamine hydrochloride (HCl) were purchased from Advent Chembio PVT. Different quantities of gas H_2_S and CH_3_SH were collected from raw wastewater samples with varying volumes.

#### Adsorption process

A wide range of factors were examined to find the ideal circumstances for the adsorption process, including temperature, dose, adsorbate initial concentration, retention time, and pH. Dispensing 20 mL of metal ions solution (0.1, 0.2, 0.4, 0.50) mg L⁻^1^ and 0.025 g L⁻^1^ of adsorbents (andesite, HPAA) in 50-ml capped borosilicate or polyethylene bottles. 0.1 M hydrochloric acid (HCl) and 0.1 M sodium hydroxide (NaOH) were utilized to modify the pH. Following agitation, suspensions were subjected to centrifugation for 10 minutess at 2500 revolutions per minute at 25 °C (± 2). The supernatant was collected for iron(III) and aluminum(III) analysis using an inductively coupled plasma (ICP-7900 MS) with argon and helium gas to achieve the required temperature to transition the metal from an excited to an ionization state to determine heavy metal concentrations. The supernatant was also collected for H_2_S and CH_3_SH analysis using the (MULTIRAE-LITE) equipment by using 20 mL in 50 mL capped borosilicate or polyethylene bottles of initial concentrations ranging from (20–100) mg L⁻^1^ and (2–10) mg L⁻^1^, respectively, and 1.00 g L⁻^1^ of the adsorbents (andesite and HPAA), utilizing the following calculations to evaluate the method’s efficiency:

3 $$\% \, R \, = \, \left[ {1 - \, \left( {C_{e} /C_{o} } \right)} \right] \, \times 100$$

4 $$Q_{e} = \, \left( {C_{o} - \, C_{e} } \right) \, V/W$$

"C_o_" represents Al(III), Fe(III), CH_3_SH, and H_2_S solution’s initial concentration in mg L⁻^1^; "C_e_" represents Al(III), Fe(III), CH_3_SH, and H_2_S solution’s final concentration in mg L⁻^1^; "V" represents the volume of Al(III), Fe(III), CH_3_SH, and H_2_S solution in L; "W" represents the adsorbent’s mass in g; "Q_e_" represents the equilibrium adsorption capacity in (mg g⁻^1^)^39^. Figures S1 and S2 provide a quick explanation of the procedure.

#### Instruments

FTIR spectra of andesite and the HPAA composite were recorded using a Thermo Scientific-Nicolet iS10 FTIR Spectrometer (USA) within the 400–4,000 cm⁻^1^ range, both before and after the adsorption process. A JEOL JSM 6510 Iv scanning electron microscope (SEM, England) was utilized to observe the surface morphology of andesite prior to and following adsorption of Al(III), Fe(III), CH_3_SH, and H_2_S. The specific surface area and porosity were analyzed through Brunauer–Emmett–Teller (BET) measurements using the BELSORP-miniX-10185 from Chromatic Tech, Japan. The zeta potential was determined via dynamic light scattering (DLS) with a ZetaSizer Nano Series (HT), Nano ZS from Malvern Instruments, UK. X-ray diffraction (XRD) analysis was carried out using a PANalytical X’Pert PRO diffractometer (Netherlands) to determine the crystalline structure of the synthesized materials. X-ray photoelectron spectroscopy (XPS) measurements were performed using a K-Alpha X-ray photoelectron spectrometer (Thermo Fisher Scientific, UK) to investigate the surface elemental composition and chemical states. The concentrations of CH_3_SH and H_2_S were measured with MULTIRAE-LITE, while Al(III) and Fe(III) concentrations were assessed using inductively coupled plasma mass spectrometry (ICP-MS7900; Agilent, Japan). The adsorbents were dried in a JSR-Json-050 oven (111226-42, Korea). Adsorption experiments were conducted using a Stuart CB162 magnetic stirrer (UK) and an ultrasonic bath (Branonic, 3510E-DTH, Mexico). All adsorption studies were performed on a spinning shaker (GS-10, USA) at 55 °C, which can take up to 24 h to establish equilibrium.

## Results and discussion

### Characterization

#### Fourier transform infrared (FTIR)

An FTIR spectrophotometer was used to evaluate andesite and HPAA composite before and following the metal ions adsorption (Al(III), Fe(III)) and molecules (H_2_S, CH_3_SH). The FTIR analysis displayed a variety of peaks, ranging from broad to faint signals. The peak at 3798.8 cm⁻^1^ corresponds to free hydroxyl (^-^OH) groups^40^. Absorption bands within the 3550–3200 cm⁻^1^ region suggest the presence of free hydroxyl (^-^OH) groups, along with stretching vibrations associated with OH⁻ groups in Al–OH–Al and Fe–OH–Al units within the octahedral structure of the adsorbent, as indicated in the andesite spectrum^41^. The absorption band at 3011.91 cm⁻^1^, attributed to N–H stretching, confirms the presence of amine groups, and C–C stretching at 2299.74 cm⁻^1^ ref.^40^. The C = N functional group is verified by the oxime stretching frequency at 1665.56 cm⁻^1^ ref.^42^. Additionally, the peak at 1636 cm⁻^1^ corresponds to the reversible adsorption of carbonate on the oxide surface. Furthermore, Fig. S3 highlights CH₂ group vibrations at 1480 cm⁻^1^.The stretching frequencies observed between 900 and 1250 cm⁻^1^ were attributed to Si-O. The peak at 589 cm⁻^1^ is possibly linked to the presence of Al₂O₃. A distinct peak at 531 cm⁻^1^ is attributed to Si-O-Al (octahedral) stretching vibrations. The prominent band at 464 cm⁻^1^ is likely associated with calcium silicate, while the signal at 428 cm⁻^1^ may originate from the asymmetric bending mode of Si-O^43^. Analysis of the HPAA composite spectrum reveals the appearance of additional peaks, indicating successful composite formation.

**Fig. 2:**
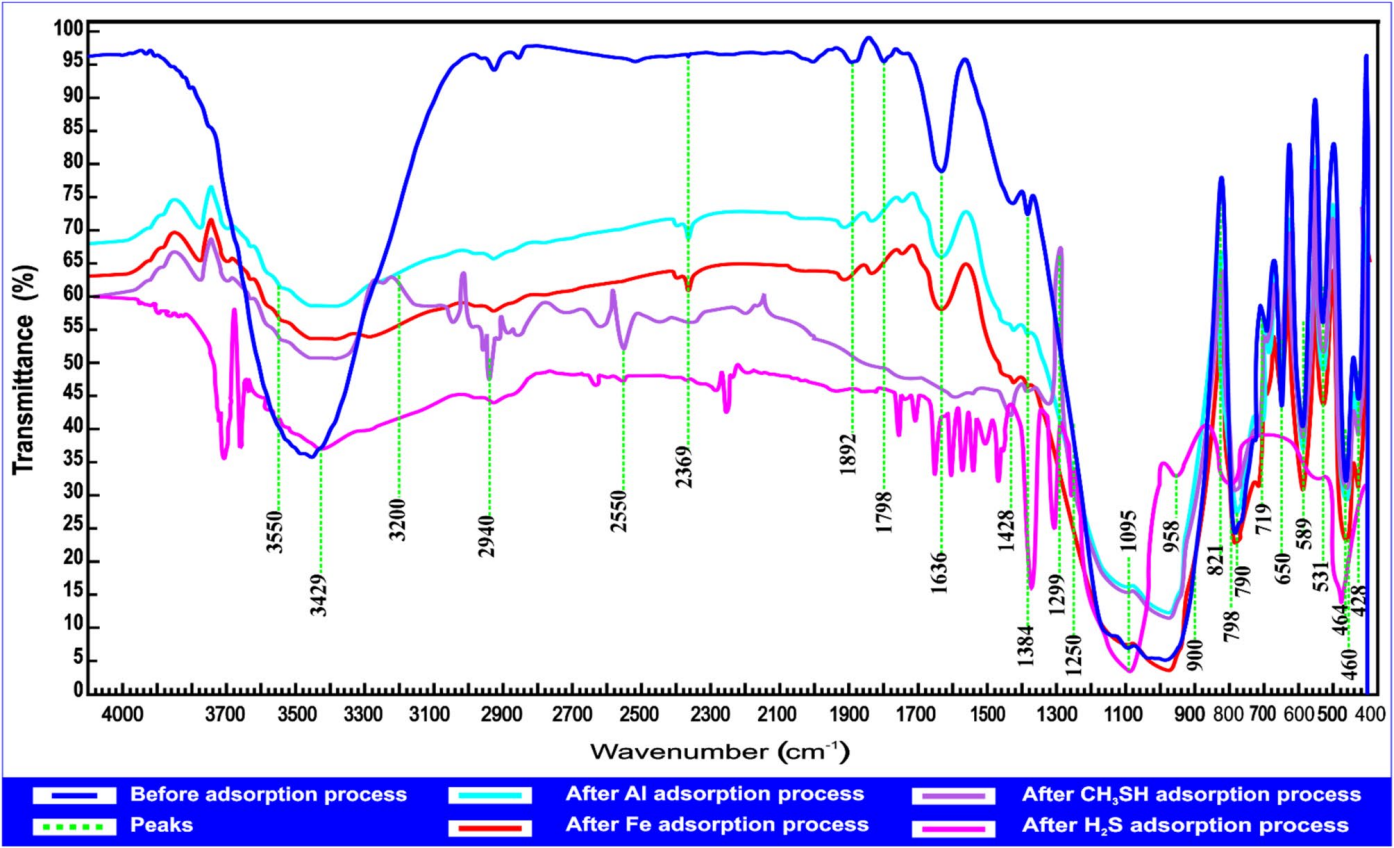
FTIR spectrophotometer analysis of andesite before and after Al(III), Fe(III), H_2_S, and CH_3_SH adsorption.

Figure 2 depicts a previous image of andesite prior to and following the adsorption of Al(III), Fe(III), H_2_S, and CH_3_SH. The vibrational stretching frequency at 2940 cm⁻^1^ is caused by the C-H bond in the alkane molecule. Thiol group (S-H) creates the peak at 2550 cm⁻^1^ ref.^44^. Al(III) adsorption may cause the peak at 790 cm⁻^1^, while the peak at 460 cm⁻^1^ is induced by Fe(III) adsorption on the Si–O group.

**Fig. 3:**
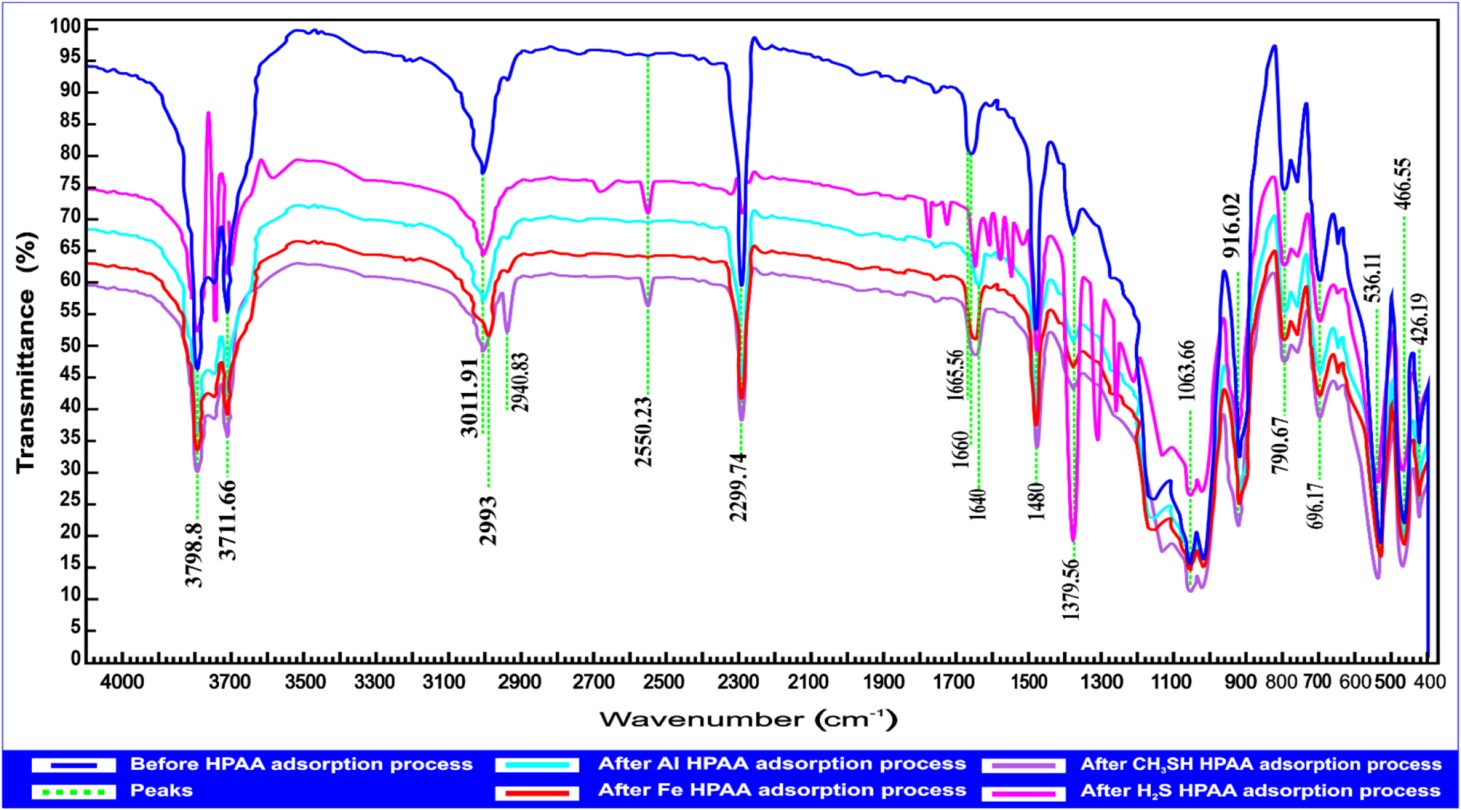
FTIR spectrophotometer analysis of HPAA composite before and after Al(III), Fe(III), H_2_S, and CH_3_SH adsorption.

Figure 3 illustrates an FTIR spectrophotometer analysis before and following adding metal ions (Al(III), Fe(III)) and molecules (H_2_S, CH_3_SH) to the HPAA composite as an adsorbent. As previously stated, new peaks occurred following the andesite alteration, with the development of 3011.91 cm⁻^1^ for N-H referring to the amine group. The peak at 2993 cm⁻^1^ suggests Fe(III) adsorption. Furthermore, the groups C-H and S-H were found at 2940.83 cm⁻^1^ and 2550.23 cm⁻^1^ ref.^44^, respectively, providing evidence for CH_3_SH and H_2_S adsorption on the HPAA composite. 1665.56 cm⁻^1^ for the C = N referring to oxime group^42^. The stretching frequency at 1660 cm⁻^1^ is indicative of Al(III) adsorption.

#### Brunauer Emmett Teller (BET)

It was employed to evaluate the surface characteristics of both natural andesite and the synthesized HPAA composite. The analysis was conducted under controlled conditions using a sample mass of 0.4 g, a manifold volume of 12.476 cm^3^, nitrogen (N₂) as the adsorptive gas, and a manifold temperature of 19.825 °C to ensure consistency and accuracy in surface area and porosity measurements.

**Fig. 4:**
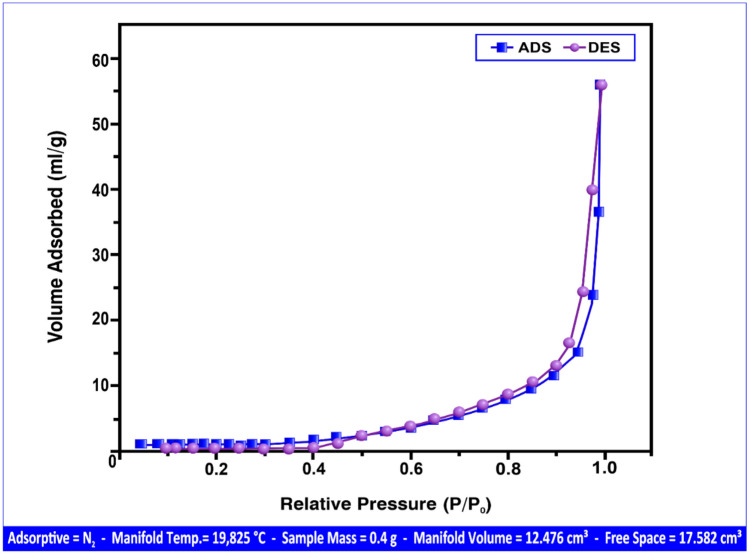
BET adsorption (ADS) and desorption (DES) isotherms of the HPAA composite.

Figure 4 illustrates the nitrogen adsorption–desorption isotherms for the HPAA composite, which revealed a type III isotherm. This type is indicative of weak adsorbate–adsorbent interactions and is commonly associated with macroporous materials, characterized by pore diameters greater than 50 nm^37^. The isotherm shape confirms the macroporous structure of the HPAA composite, aligning with IUPAC classification standards.

Quantitative BET results further substantiated this observation. The specific surface area and average pore diameter of andesite were determined to be 1.281 m^2^/g and 92.231 nm, respectively^38^. In contrast, the HPAA composite exhibited a significantly higher specific surface area of 5.020 m^2^/g and an average pore diameter of 283.331 nm. These values clearly support the macroporous classification for both materials, with the HPAA composite demonstrating enhanced surface properties due to its higher porosity and surface area.

The presence of large pore sizes is particularly advantageous for adsorption processes involving bulky or multivalent species. In this study, the macroporous structure facilitated the effective diffusion and accessibility of metal ions such as Al(III) and Fe(III)^45^, as well as gaseous pollutants including hydrogen sulfide (H_2_S) and methyl mercaptan (CH_3_SH)^46^. The improved permeability of both materials allowed for efficient interaction between the adsorbate molecules and the internal adsorption sites, ultimately enhancing their adsorption capacities.

Moreover, the higher surface area of the HPAA composite suggests a greater availability of active sites, which likely contributes to its superior performance compared to natural andesite. The synergistic effect of increased surface area and macropore volume underscores the HPAA composite’s potential for advanced environmental applications, particularly in the adsorption of metal ions and gaseous contaminants from aqueous and air phases.

#### Scanning electron microscope (SEM)

Research on andesite porosity indicates that natural andesite is an excellent adsorbent, as shown with a scanning electron microscope (SEM; JEOL JSM 6510 Iv, England), revealing a heterogeneous surface morphology comprising both smooth and rough textures. The rough surfaces exhibit trench-like structures with distinct linear formations containing macrospores, which are particularly prominent in these regions. The presence of macrospores significantly enhances the surface area^38^ of andesite, thereby improving its adsorption efficiency. Further SEM imaging at magnifications of 1 × 700 and 1 × 2000 confirmed the highly porous nature of the andesite, which is a key factor in its adsorption capabilities.

**Fig. 5:**
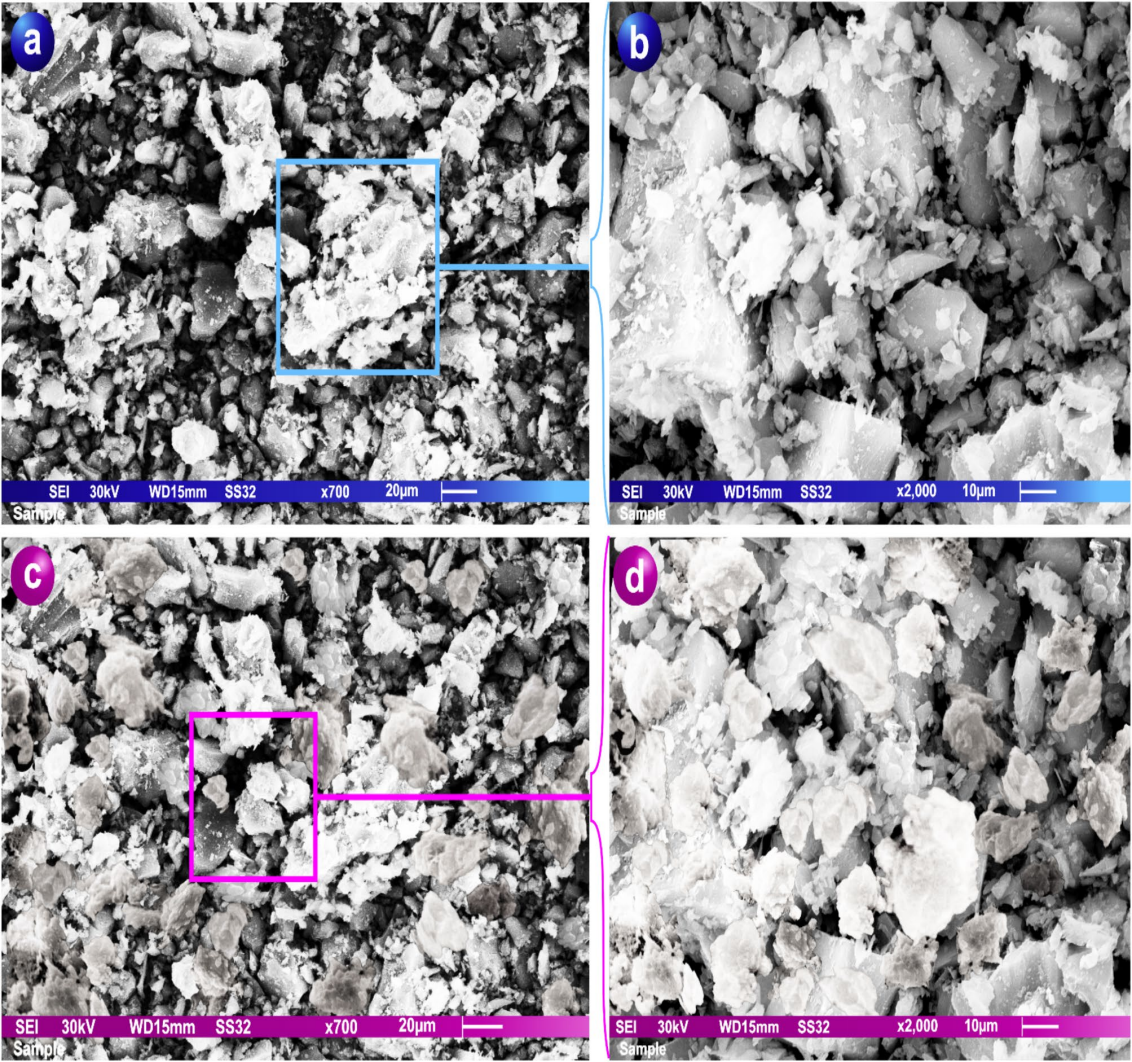
SEM of andesite at magnifications of 1 × 700 and 1 × 2,000 before (**a**, **b**) and after (**c**, **d**) Al(III), Fe(III), H_2_S, and CH_3_SH adsorption.

Figure 5 presents SEM micrographs of andesite at magnifications of 1 × 700 (a, b) and 1 × 2000 (c, d), capturing its surface morphology before (a, c) and after (b, d) adsorption of Al(III), Fe(III), H_2_S, and CH_3_SH. Prior to adsorption, the SEM images (Fig. 5a,c) reveal the heterogeneous and highly porous nature of the andesite, with a combination of smooth and rough textures. The rough areas are marked by trench-like features and pronounced linear structures interspersed with macrospores, which play a critical role in enhancing the specific surface area and thus the adsorption efficiency^47^.

In addition to its morphological features, the mineralogical composition of andesite includes aluminum silicates and various associated elements such as calcium (Ca), copper (Cu), iron (Fe), potassium (K), and sodium (Na), contributing to the material’s structural stability and potential surface reactivity.

Post-adsorption images (Fig. 5b,d) demonstrate the successful loading of Al(III), Fe(III), H_2_S, and CH_3_SH onto the andesite surface. The adsorbed species partially fill the pores and coat the surface, indicating effective interaction between the adsorbates and the porous matrix of the mineral. The comparison between pre- and post-adsorption states highlights the capacity of natural andesite to function as an efficient and sustainable adsorbent^48^, leveraging its inherent porosity, extensive surface area, and chemical composition to capture diverse inorganic and organic contaminants.

Overall, the SEM observations provide strong evidence supporting the use of andesite in environmental remediation applications, particularly for the adsorption of metal ions and sulfur-containing compounds.

#### Zeta potential

A separate zeta potential^38^ assessment was conducted on andesite using the same analytical technique. Typically, a threshold zeta potential of ± 30 mV is employed to differentiate stable suspensions from unstable ones.

Particles exhibiting zeta potential values beyond ± 30 mV are generally considered stable due to enhanced electrostatic repulsion, which mitigates aggregation. The zeta potential of the HPAA composite was measured under the following conditions: a clear disposable zeta cell, an attenuator setting of 7, a temperature of 24.9 °C, a measurement duration of 60 s, a count rate of 156.10 kcps, and a measurement location of 2.20 mm^49^.

**Fig. 6:**
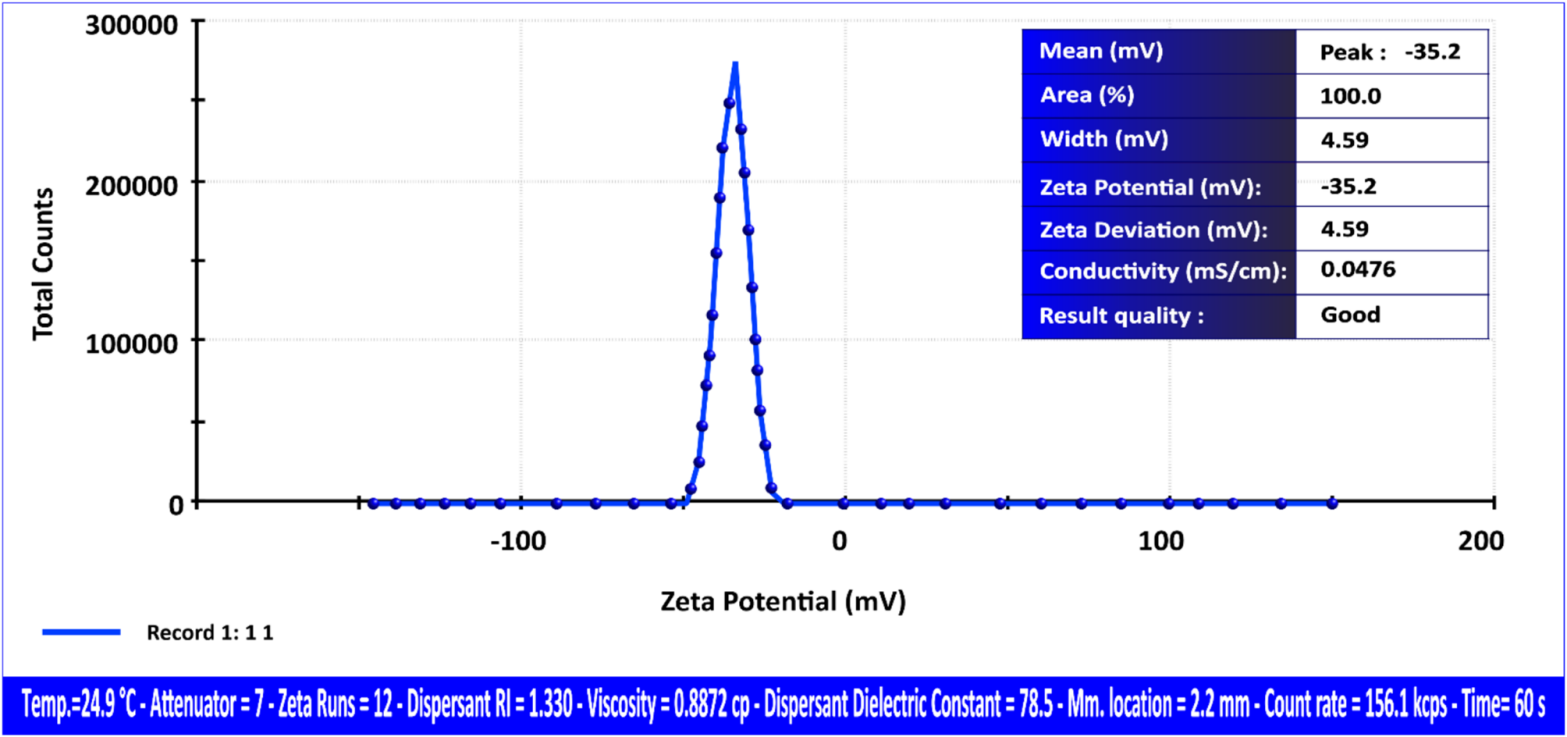
Zeta potential distribution of HPAA composite.

As depicted in Fig. 6, the HPAA composite demonstrates a zeta potential of -35.20 mV, indicating a pronounced negative surface charge. This substantial negative potential suggests excellent colloidal stability, which is critical in maintaining dispersion in aqueous systems and preventing particle aggregation. More importantly, the negative charge enhances the material’s affinity for positively charged contaminants such as Al(III) and Fe(III) ions, facilitating their efficient adsorption. Additionally, the negative surface potential likely contributes to the composite’s capacity to interact with and remove neutral or weakly polar species such as H_2_S and CH_3_SH, possibly through mechanisms such as hydrogen bonding or van der Waals interactions. Collectively, these findings underscore the HPAA composite’s promising application in wastewater treatment and contaminant removal, providing a strong foundation for further exploration of its performance under varied environmental conditions and against a broader range of pollutants.

#### X-ray diffraction (XRD)

**Fig. 7:**
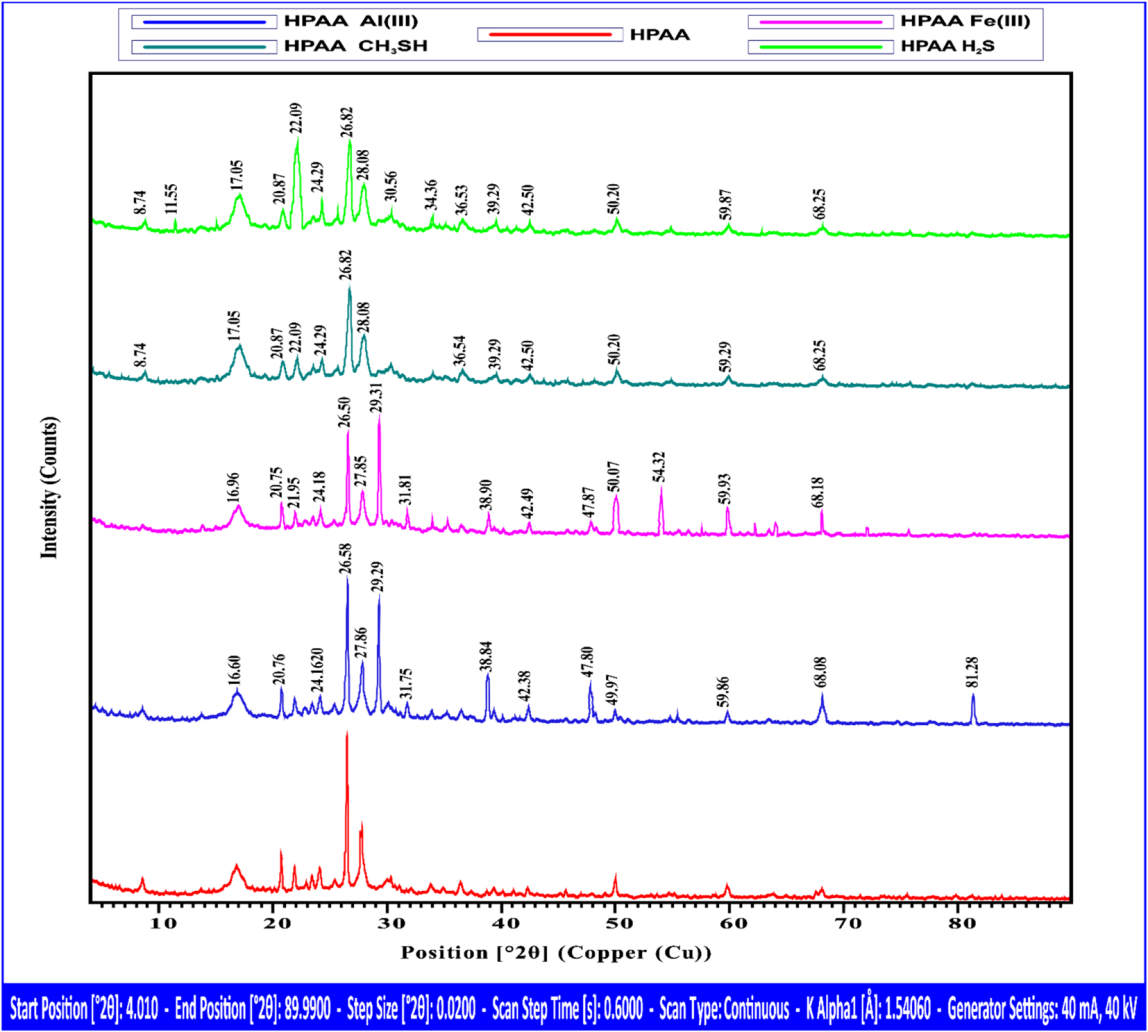
XRD analysis of HPAA composite, before and after Adsorption of Al(III), Fe(III), H_2_S, and CH_3_SH.

The composite (poly AN-Andesite) HPAA before and after Al(III), Fe(III), H_2_S, and CH_3_SH revealed the X-ray diffraction pattern. All five samples underwent identical measurements (40 mA, 40 kV, continuous scan, 1.5406 Å radiation), as shown in Fig. 7. With a step size of 0.02°, the scan range is 4.01° to 89.99° 2θ.

As noted in Fig. 7, each dataset exhibits peaks at distinct 2θ values, suggesting variations in the crystallographic structure, and the highest intensity peaks for each sample change, indicating different dominant phases or structural shifts. This is the outcome of the structural interpretation in Table 3.

**Table 3:** Structural interpretation of samples.

Sample	Highest intensity peak (2θ)	Notable differences and expected phases	Crystallite size (nm)
HPAA	26.49° (197.36 cts)	The strong peak at 26.49°, smaller peaks spread out. Pure reference phase (likely a metal aluminosilicate)	13.11
HPAA Al(III)	26.58° (151.34 cts)	Major peaks at 29.29° and 27.87°, suggesting Al^3+^ alters the structure or new Al-oxide phase^51^	9.85
HPAA Fe(III)	29.31° (142.93 cts)	The highest intensity shifts to 29.31°, peak at 26.56° is weaker. Fe^3+^ causes structural shifts or iron oxide formation^52^	10.10
HPAA CH_3_SH	26.83° (86.31 cts)	Lower overall intensities, and broad peaks, indicating possible amorphous content and organic-sulfur interaction^53^	7.17
HPAA H_2_S	26.82° (86.31 cts)	Peaks around 36.53° and 39.29° could be associated with and lowest crystallinity, possible sulfide compound formation^54^	7.71

We may get the average crystallite size by applying the Scherrer equation^50^:

5 $$D \, = \frac{K\lambda }{{\beta \cos \theta }}$$

For Cu-Kα, "λ" is the X-ray wavelength (1.5406 Å); "β" is the Full Width at Half Maximum (FWHM, in radians); "θ" is the Bragg angle (in degrees); and "D" is the crystallite’s size (in nm); "K" is the form factor, often 0.9.

The adsorption of Al^3^⁺, Fe^3^⁺, H_2_S, and CH_3_SH can create structural distortions, flaws, and amorphous layers, resulting in smaller crystallites. It was shown that surface contacts, amorphization, and nanophase formation^55,56^ are the main causes of the crystallite size decrease following adsorption.

#### X-ray photoelectron spectroscopy (XPS)

It was employed to elucidate the surface chemical modifications of HPAA composite, especially following functionalization with Al(III), Fe(III), CH_3_SH, and H_2_S. These modifications are critical to understanding the material’s potential for environmental applications, including adsorption and catalysis^57^. The XPS survey spectrum of the HPAA composite revealed distinct peaks corresponding to C 1s (~ 285 eV), attributed to aliphatic C–C and C–H, bonds from the HPAA polymer backbone**.** O 1s (~ 531 eV)**,** associated with lattice oxygen and hydroxyl groups. Si 2p (~ 102 eV)**,** indicative of the silicate structure of andesite^45^. N 1s (~ 400 eV)**,** suggesting the presence of oxime functionalities from HPAA. Elemental identification and chemical state assignments were locates in Table 4.

**Table 4:** Summary of expected XPS features and assignments.

Element	Peak (ev)	Chemical state	Assignment
C 1S	~ 285	C–C, C–H	Polymer backbone
O 1S	~ 531	Lattice O, OH	Matrix and surface reactivity
N 1S	~ 400	–NH, –CN-OH–	Polymer oxime groups
SI 2P	~ 102	Si–O	Andesite silicate structure
Al 2P	~ 74	Al^3^⁺	Incorporated aluminum species
Fe 2P	~ 710–724	Fe^3^⁺	Incorporated iron species
S 2P	~ 162–170	S^2^⁻, S⁶⁺	Sulfide/sulfonate functionality

**Fig. 8:**
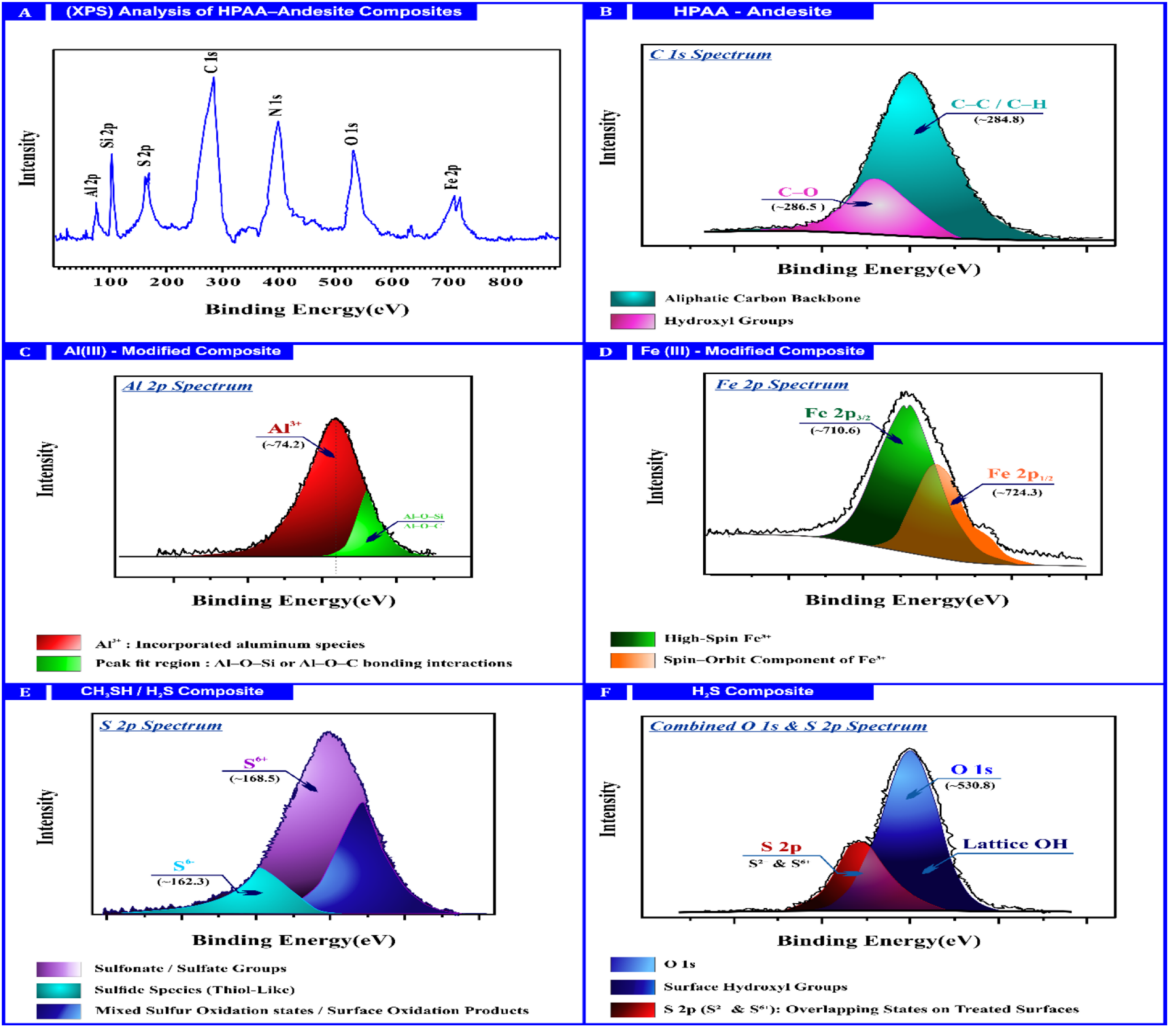
X-ray Photoelectron Spectroscopy for HPAA composite before and after adsorption process.

X-ray photoelectron spectroscopy (XPS) analysis confirmed the successful incorporation of aluminum species into the composite upon treatment with Al(III), as evidenced by the emergence of a distinct Al 2p peak at approximately 74 eV. This observation suggests the presence of Al^3+^ ions, likely forming Al–O bonds through interactions with hydroxyl or carboxyl groups within the polymer matrix. Supporting this, minor shifts observed in the O 1 s spectrum indicated changes in the local electron density, further implying Al–O bonding interactions. Similarly, in the Fe(III)-modified sample, XPS analysis revealed well-defined Fe 2p_3/2_ and Fe 2p_1/2_ peaks at approximately 710 and 724 eV, respectively, accompanied by characteristic satellite features typical of Fe^3+^ species. These spectral signatures confirmed that ferric ions were chemically integrated into the composite structure, most plausibly via Fe–O–C linkages. Correspondingly, the O 1 s spectrum exhibited increased intensity and minor peak splitting, indicative of enhanced interactions between Fe species and oxygen-containing functionalities within the material. In the case of sulfur-treated composites exposed to CH_3_SH and H_2_S, the incorporation of sulfur was validated by the appearance of S 2p peaks within the 162–170 eV range. These included components attributable to S^2-^ at ~ 162 eV, pointing to the formation of sulfide species, and S^6+^ in the 168–170 eV region, indicating partial oxidation likely resulting in the formation of sulfonate or sulfate groups. The C 1 s spectra revealed the presence of C–S bonding environments, while subtle shifts in the O 1 s peaks suggested modifications in surface acidity and reactivity^45^. As shown in Fig. 8, these spectral features collectively highlight the successful functionalization of the composite surface with sulfur-containing species and their impact on the chemical environment.

#### Retention time influence

**Fig. 9:**
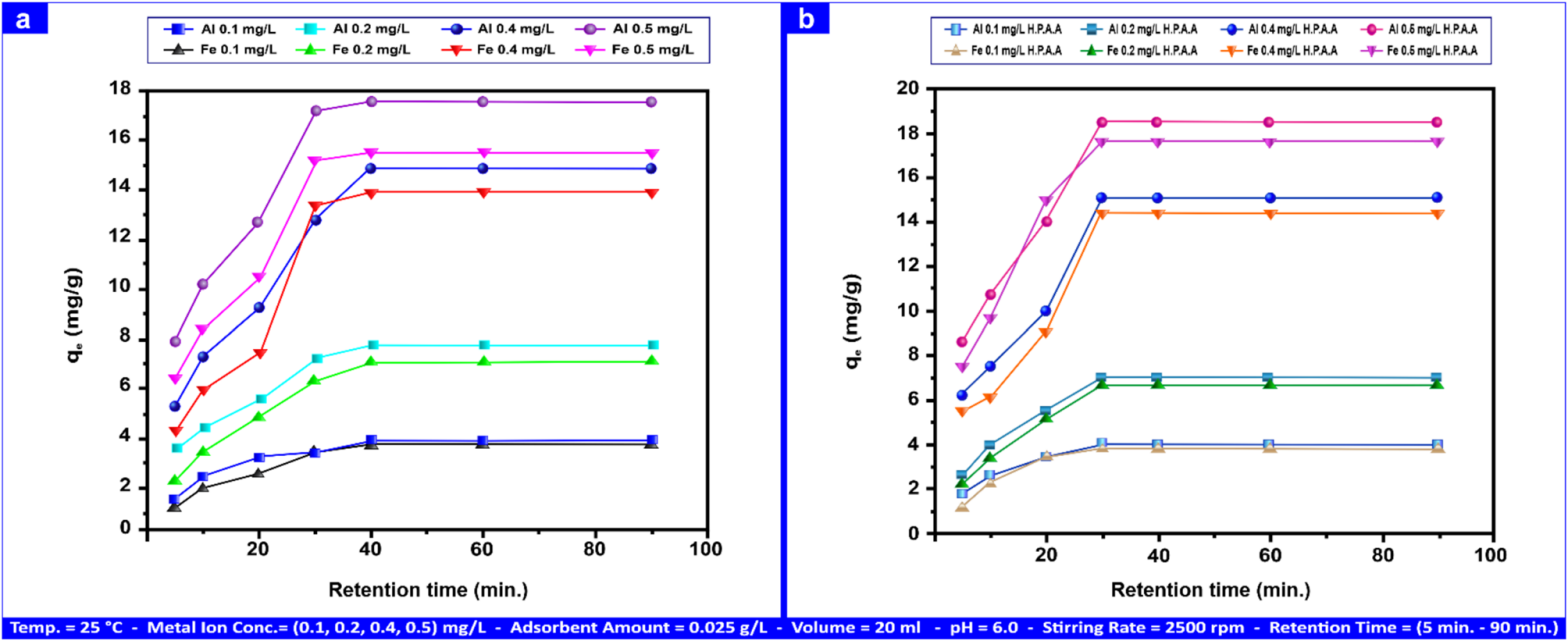
Retention time influence on Al(III) and Fe(II) adsorption on andesite (**a**) and HPAA composite (**b**).

As demonstrated in Fig. 9a using the subsequent parameters: 20 ml of solution volume, pH 6.0, (0.1, 0.2, 0.4, 0.50) mg L⁻^1^ of metal ions, 0.025 g L⁻^1^ of adsorbents, and an 2500 rpm stirring rate. The retention times of Al(III) and Fe(III) adsorption were investigated at 5 to 90 min intervals. The target adsorbates Al(III) and Fe(III) showed different adsorption capacities in each study. When the metal ion achieved equilibrium, it was separated using a centrifuge, and the filtrate was found using an inductively linked plasma mass spectrometer^58^. Concerning Fig. 9a, we found that for the two cations, Al(III) and Fe(III), equilibrium is reached at 30 min. The adsorption process and retention time^59^ might be exactly proportional.

Because of the tight relationship involving adsorption capacity and the number of active sites, the interaction of positive cations from the liquid’s main phase with negative empty adsorption sites^60^ accelerated the adsorption process. The maximum adsorption capacity for Al(III) and Fe(III) was 17.35 mg g⁻^1^ for Al(III) and 15.39 mg g⁻^1^ for Fe(III) at a concentration of 0.5 mg L⁻^1^, reaching equilibrium after 30 min of shaking. The andesite also showed a high affinity for Al(III) and Fe(III). From 5 to 30 min, Al(III) and Fe(III) adsorption capacity increases from 1.25 to 17.35 mg g⁻^1^ and 0.89 to 15.39 mg g⁻^1^ at concentrations of 0.1 to 0.5 mg L⁻^1^, respectively. Before saturation, the adsorption capacity stays constant for 30 to 60 min. The equilibrium sorption capacity of the two metal ions was Al > Fe, showing that hydroxyl, aluminol, and silanol groups bind to andesite. According to the hydrated cations diameter (Al^+3^ ionic radius = 53.00 pm and Fe^+3^ ionic radius = 78.50 pm) and the cation charge density (Al^+3^ > Fe^+3^), the pattern that was observed was most likely caused by adsorption processes, which led to a faster equilibrium for Al ions^61^ than Fe ions. Figure 9b depicts the adsorption process employing hydrolysis poly acrylonitrile andesite HPAA, as 18.60 mg g⁻^1^ was the greatest adsorption capability for Al(III) and 17.76 mg g⁻^1^ for Fe(III) at concentrations of 0.5 mg L⁻^1^ for both Al(III) and Fe(III). From 5 to 30 min, Al(III) and Fe(III) adsorption capacity increases from 1.75 to 18.60 mg g⁻^1^ and 1.20 to 17.76 mg g⁻^1^ at concentrations ranging from 0.1 to 0.5 mg L⁻^1^, respectively. Because of its larger surface area and additional active groups, such as oxime groups (C = N‒OH) and NH_2_, compared to andesite, the HPAA composite has a greater adsorption capability. CH_3_SH and H_2_S adsorption were investigated at 25 °C, pH 9.0, stirring rate of 2500 rpm, gas concentrations of (100 and 10) mg L⁻^1^ for H_2_S and CH_3_SH, respectively, andesite and HPAA weight of 1.00 g L⁻^1^. The retention time of CH_3_SH and H_2_S adsorption was measured at intervals of 2 to 25 min. After 10 min of shaking, equilibrium was achieved, with the highest adsorption capacity of 94.03 mg g⁻^1^ for H_2_S and 9.02 mg g⁻^1^ for CH_3_SH at concentrations of 100 mg L⁻^1^ and 10 mg L⁻^1^, respectively, as shown in Fig. S4a. The short retention time for gas evacuation may be due to an adsorption process on the andesite surface. Figure S4b depicts how retention time affects H_2_S and CH_3_SH adsorption on HPAA composite has a maximum adsorption capacity of 98.00 mg g⁻^1^ for H_2_S and 9.60 mg g⁻^1^ for CH_3_SH at concentrations between 100 and 10 mg L⁻^1^. HPAA composite have a higher adsorption capacity than andesite.

#### Dose of adsorbent influence

**Fig. 10:**
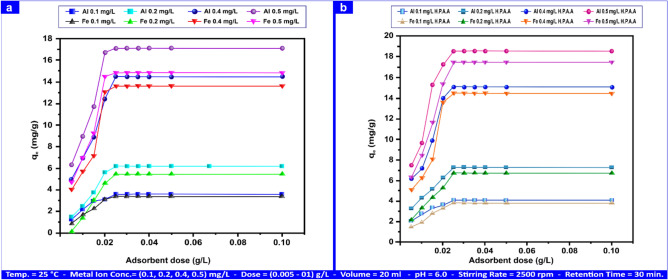
Various adsorbent doses influence Al(III) and Fe(II) adsorption on andesite (**a**) and HPAA composite (**b**).

The primary purpose of this research was to remove the desired metal ion concentration using the least amount of adsorbent. Further investigations were conducted under modified conditions, including a solution pH of 6.0, a retention time of 30 min, and a stirring rate of 2500 rpm. The study examined metal ion concentrations of 0.1, 0.2, 0.4, and 0.5 mg/L while varying adsorbent doses from 0.005 g/L to 0.1 g/L. Figure 10a shows the effects of different dosages.

The adsorption capacity increased from 0.005 to 0.025 g L⁻^1^ of sorbent and thereafter remained quite stable. Because andesite can remove Al(III) and Fe(III) from their surface via adsorption on unoccupied active sites, adsorption will occur till the sites are saturated. Subsequently, it was observed that there were multiple feasible adsorption sites within the andesite surface, having significant energy differences depending on whether the site was inside the fault site^62^ or outside. Later research revealed that the andesite surface included a range of viable adsorption sites, each with a significant energy differential depending on whether their location was on the edge or in the defect.

Both uniformity and specificity were lacking in the adsorption sites^62^. Equilibrium was achieved with the highest capacity for adsorption of 17.20 mg g⁻^1^for Al(III) and 15.16 mg g⁻^1^ for Fe(III) at concentrations of 0.5 mg L⁻^1^ for both Al(III) and Fe(III) and an andesite dose of 0.025 g L⁻^1^. At 25 °C, 20 ml of solution, pH 6.0, 30 min of retention, 2500 rpm stirring rate, metal ion concentrations of (0.1, 0.2, 0.4, and 0.50) mg L⁻^1^, and HPAA, the greatest adsorption capacity was 18.8 mg g⁻^1^ for Al(III) and 17.10 mg g⁻^1^ for Fe(III) at concentrations of 0.5 mg L⁻^1^ for both Al(III) and Fe(III) 0.0.025 g L⁻^1^, as Fig. 10b illustrates.

The adsorption capacity grew at doses ranging from 0.005 to 0.1 g L⁻^1^ because the HPAA composite has more unoccupied active sites on its surface than the andesite. Gases, CH_3_SH, and H_2_S removal were studied at concentrations of 100 mg L⁻^1^ for H_2_S and 10 mg L⁻^1^ for CH_3_SH, at 25 °C, pH 9.0, 2500 rpm of stirring rate, andesite and HPAA dose of 0.02–2 g L⁻^1^, and a retention time of 10 min.

Figure S5a demonstrates that equilibrium was reached with a maximum adsorption capacity of 95.12 mg g⁻^1^ for H_2_S and 9.25 mg g⁻^1^ for CH_3_SH at concentrations of (100 and 10) mg L⁻^1^, respectively, and an adsorbent dose of 1.00 g L⁻^1^. According to earlier research, andesites with a vesicular or porous structure and a porphyritic texture exhibited the highest adsorption capacity.

Figure S5b shows that the maximum adsorption capacity on the HPAA composite surface was 99.00 mg g⁻^1^ for H_2_S and 9.78 mg g⁻^1^ for CH_3_SH at concentrations of 100 and 10 mg L⁻^1^, correspondingly and adsorbent dose of 1.00 g L⁻^1^.

#### Effect of pH

The hydrogen concentration of ions is a highly essential variable regulating the adsorption process since it determines the solubility of metal ions, the concentration of counterions on the functional groups of the adsorbent, and the extent of ionization of the adsorbate during reaction^63,64^. The temperature, solution volume, sorbent dosage, retention time, shaking time, and initial metal ion concentration for Al(III) and Fe(III) were set to 25 °C, 20 mL, 0.025 g L⁻^1^, 30 min, 2500 rpm, and (0.1, 0.2, 0.4, and 0.50) mg L⁻^1^, respectively. The study looked at the effects of pH on adsorbent surface charge by varying the pH between 2, 3, 4, 6, 7, and 8.

**Fig. 11:**
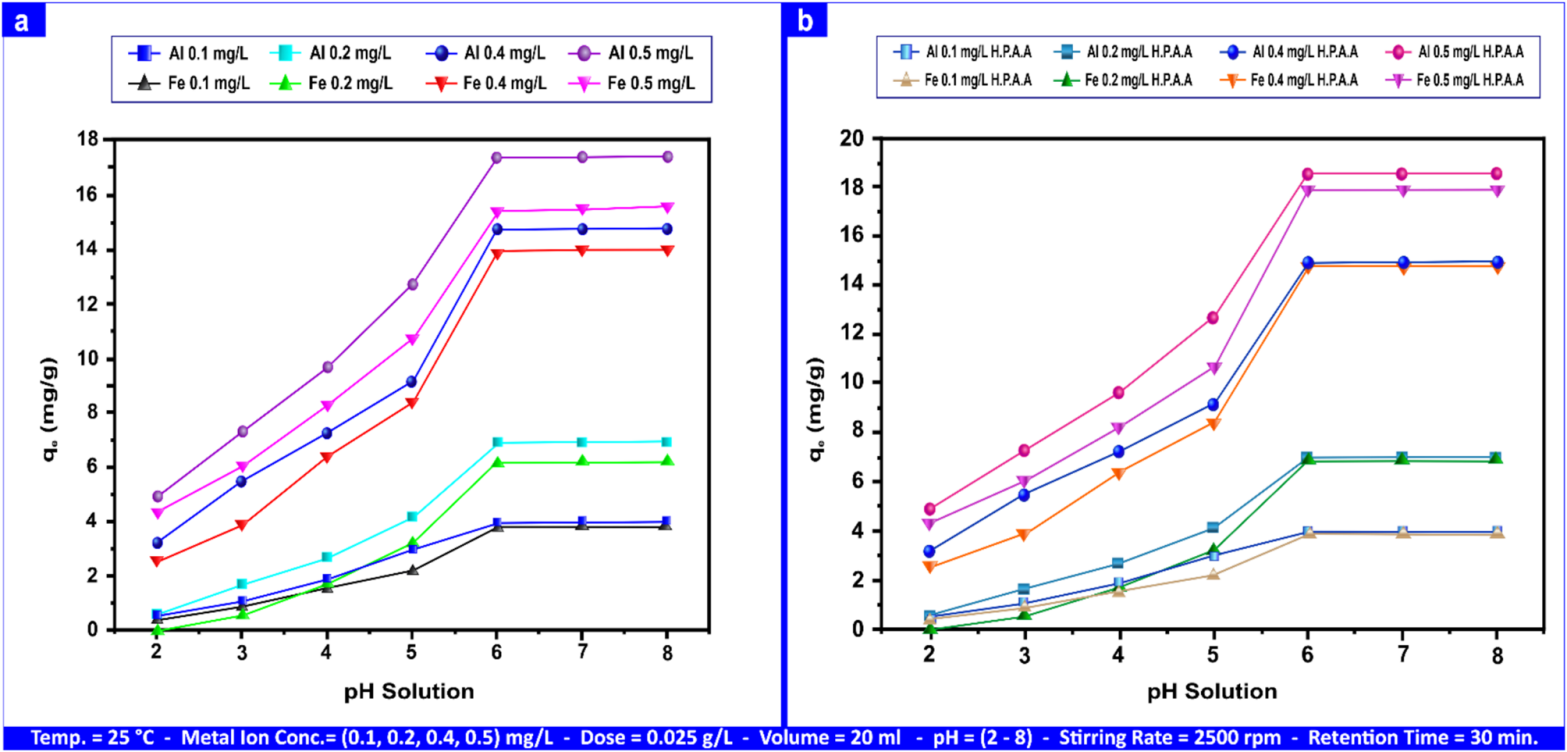
pH influence on Al(III) and Fe(III) adsorption on andesite (**a**) and HPAA composite (**b**).

Figure 11a illustrates how the pH of the initial solution affected adsorption, demonstrating that as pH increased, so did adsorption capacity. According to the graph, adsorption increases from 2 to 8 and achieves its maximum rate at pH 6.

The highest values of the adsorption capacity for Al(III) and Fe(III) were 3.76, 7.48, 14.77, and 17.43 mg g⁻^1^ and 3.68, 6.79, 13.9, and 15.5 mg g⁻^1^, accordingly, when the pH of the solution increased from 2 to 8. Adsorption capacity rose from 0.55 to 17.34 mg g⁻^1^ for Al(III) and from 0.42 to 15.5 mg g⁻^1^ for Fe(III) at concentrations ranging from 0.1 to 0.5 mg L⁻^1^ and pH values around 2 and 6.

After pH > 6, the adsorption rate of Al(III) and Fe(III) increased somewhat, suggesting that pH 6 is the ideal value for Al(III) and Fe(III) adsorption. This is due to the presence of negatively charged active sites like silanol (Si-OH) and aluminol (Al-OH) on the andesite surface.

In general, metal removal decreased at a low pH level. The increased H^+^ concentration^65^ may be responsible for the rivalry among metal ions and H^+^ for adsorption sites. Additionally, because of the sorbent’s surface’s positive charge, metal cations are less drawn to it. The adsorbent’s surface gains a negative charge as pH rises, creating electrostatic interactions beneficial for adsorbing cationic molecules^66^.

As pH rose, so did the sorption of metal cations, suggesting that mechanisms, ion interaction with different ions connected to an acidic functional group, or surface complexation made metal ionic species less durable in solution. Silanol (Si-OH) and aluminol (Al-OH) groups are crucial for metal adsorption efficiency. At pH levels greater than 3–4, (Al–OH) and (Si0OH) groups are deprotonated and negatively charged. As a result, positively charged metal ions would attract more readily. Although partial metal desorption may happen as a result of the interaction of metal ions with OH ions, precipitating form a metal hydroxide^67^, raising the pH after saturation does not encourage metal sorption.

At 25 °C, 20 mL, 0.025 g L⁻^1^, 30 min., 2500 rpm, (0.1, 0.2, 0.4, and 0.50) mg L⁻^1^, and a solution pH of 2 to 8, the adsorption capacity increased, with maximum values of (3.90, 7.62, 14.99, and 18.63) mg g⁻^1^ for Al(III) and (3.90, 7.51, 14.79, and 17.99) mg g⁻^1^ for Fe(III). On the HPAA Compositesurface, adsorption capacity grew from 0.95 to 18.63 mg g⁻^1^ for Al(III) and 0.70 to 17.99 mg g⁻^1^ for Fe(III) at concentrations ranging from 0.1 to 0.5 mg L⁻^1^ and pH ranging from 2 to 6, as shown in Fig. 11b. The hydrolysis of the PAA compositeincreases the number of active groups, including NH₂, aluminol (Al-OH), silanol (Si-OH) groups, and an oxime group (C = N‒OH) by converting the nitrile group (C≡N).

With a pH change from 2 to 10, a stirring rate of 2500 rpm, an andesite dosage of 1.00 g L⁻^1^, a retention time of 10 min, and concentrations between (100 and 10) mg L⁻^1^, Fig. S6a illustrates how the initial solution pH affects adsorption for H_2_S and CH_3_SH. Figure S6a shows that adsorption increases from 2 to 10 and achieves its maximum value at pH 8. The adsorption capacity increased considerably as the solution pH grew from 2 to 10, with the highest being 94.48 mg g⁻^1^ for H_2_S and 9.08 mg g⁻^1^ for CH_3_SH.

The adsorption capacity rose from (32.50 to 94.48) mg g⁻^1^ and from 1.25 to 9.08 mg g⁻^1^ at concentrations of (20 to 100) and (2 to10) mg L⁻^1^ for H_2_S and CH_3_SH, at pH ranging from 2 to 10, indicating that the ideal pH value for adsorption of H_2_S and CH_3_SH was 8 and that a pH greater than 8 was preferable for the synchronous elimination of gaseous hydrogen sulfide. At basic pH, the thiol group deprotonated (reaction pH 9.3)^68,69^.

Figure S6b shows how pH 8 maintained the maximum adsorption capacity, which increased by the use of HPAA Composite, reaching 99.40 mg g⁻^1^ for H_2_S and 9.85 mg g⁻^1^ for CH_3_SH. Figure S6b shows that at concentrations of 10 to 100 mg L⁻^1^ for H_2_S. The adsorption capacity of CH_3_SH, increased between 44.45 and 99.40 mg g⁻^1^ and 2.45 and 9.85 mg g⁻^1^, respectively.

The adsorption performance of the composite material was evaluated as a function of pH, initial concentration, and the presence of HPAA, with a comparative analysis between metal ions and gas-phase species. Regarding pH dependence, the adsorption capacity was found to improve progressively with increasing pH values. For Al(III) and Fe(III), adsorption stabilized beyond pH 6, whereas for the gaseous species H_2_S and CH_3_SH, equilibrium was achieved after pH 8. In terms of concentration dependence, increasing the initial concentrations of metal ions led to a corresponding increase in adsorption capacity, indicating a strong driving force for mass transfer at higher concentrations. The influence of HPAA was also significant, as it consistently enhanced adsorption efficiency across all target analytes (Al(III), Fe(III), H_2_S, and CH_3_SH) with the most notable improvements observed under higher concentration and alkaline pH conditions. When comparing the adsorption behavior of the two metal ions, Al(III) demonstrated superior adsorption performance relative to Fe(III) under all experimental conditions tested, suggesting a stronger affinity or more favorable interaction mechanisms with the composite surface.

#### Pollutants initial ion concentration

**Fig. 12:**
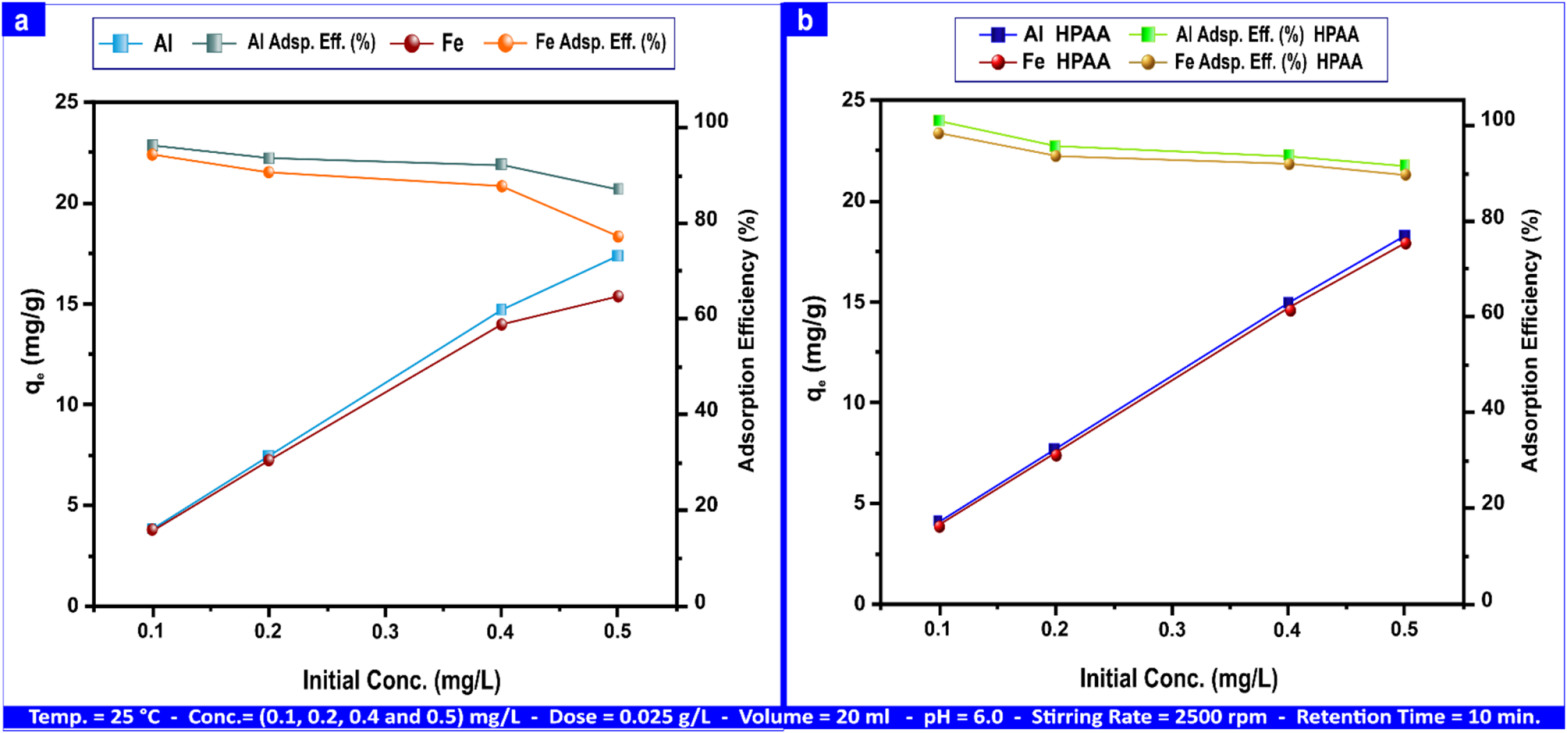
The influence of metal ion concentration on the adsorption of Al(III) and Fe(III) on andesite (**a**) and HPAA composite (**b**).

To provide optimal Al(III) and Fe(III) concentrations and high adsorption capacity on andesite surface, this factor was studied at the temperature parameters, solution volume, pH, sorbent dosage, retention time, shaking time, and initial metal ion concentration, which were set at (25 °C, 20 mL, 6, 0.025 g L⁻^1^, 30 min., 2500 rpm, and (0.1, 0.2, 0.4, and 0.50) mg L⁻^1^). Because the adsorbent (Andesite and HPAA composite) has particular devoid active spots that attract metal ions until they are saturated^37^ at constant adsorbent doses, the adsorption process is inversely related to adsorption efficiency% as well as directly proportional to adsorption capacity^70^, as shown by the data in Fig. 12a. The maximum adsorption capacity for Al(III) and Fe(III) was recorded at 17.35 and 15.39 mg g⁻^1^, accordingly, when the initial concentration was 0.5 mg L⁻^1^. In contrast, the minimum adsorption capacity was 3.84 mg g⁻^1^ for Al(III) and 3.76 mg g⁻^1^ for Fe(III) at an initial concentration of 0.1 mg L⁻^1^. The adsorption efficiency on the andesite surface reached 96% for Al(III) and 94.08% for Fe(III) at an initial concentration of 0.1 mg L⁻^1^, whereas at 0.5 mg L⁻^1^, the efficiency was 86.77% and 76.97% for Al(III) and Fe(III), correspondingly.

Figure 12b depicts the adsorption process on the HPAA compositesurface, with maximum adsorption capacities of 18.15 and 17.79 mg g⁻^1^ for Al(III) and Fe(III) at an initial concentration of 0.5 mg L⁻^1^, 4.00 mg g⁻^1^ and 3.90 mg g⁻^1^ at a starting concentration of 0.1 mg L⁻^1^ for both metal ions. For Al(III) and Fe(III), the absorption efficiency% were (100 and 97.58) at an initial concentration of 0.1 mg L⁻^1^, and (90.77 and 88.97) at an initial concentration of 0.5 mg L⁻^1^.

The impact of initial ion concentration on H_2_S and CH_3_SH removal was examined at 25 °C and concentrations varying from 20 to 100 mg L⁻^1^ and 2 to 10 mg L⁻^1^ for H_2_S and CH_3_SH, correspondingly, with a solution volume of 20 ml, pH 9, stirring rate of 2500 rpm, an adsorbent dose of 1 g L⁻^1^, and a retention duration of 10 min.

Figure S7a demonstrates that the adsorption process is directly proportional to adsorption capacity and inversely correlated with adsorption efficiency%. The maximal adsorption capacity and adsorption efficiency% were (94.48 mg g⁻^1^, 97.50) at (20 to 100) mg L⁻^1^ for H_2_S and (9.08 mg g⁻^1^, 95.00) at (2 to 10) mg L⁻^1^ for CH_3_SH.

Figure S7b shows an adsorption process on hydrolysis polyacrylonitrile andesite HPAA at 25 °C and concentrations varying from 20 to 100 mg L⁻^1^ and 2 to 10 mg L⁻^1^ for H_2_S and CH_3_SH, correspondingly, with a solution volume of 20 ml, pH 9, stirring rate of 2500 rpm, an adsorbent dose of 1 g L⁻^1^, and a retention duration of 10 min, with maximal adsorption capacity and adsorption efficiency% were (98.40 mg g⁻^1^, 100.00) at (20 to 100) mg L⁻^1^ for H_2_S and (9.75 mg g⁻^1^, 99.00) at (2 to 10) mg L⁻^1^ for CH_3_SH.

HPAA modification drastically enhances both adsorption capacity and removal efficiency for Al and Fe compared to unmodified andesite. The composite ensures more uniform and stronger metal uptake across a wider range of concentrations, likely due to increased functional groups (Si–OH, Al–OH, C = N–OH, NH_2_) providing more and stronger binding sites. The HPAA-modified andesite significantly improves adsorption performance for both H_2_S and CH_3_SH, consistent with Langmuir monolayer adsorption. The modification increases the number and strength of adsorption sites, particularly benefiting the less-adsorptive CH_3_SH.

#### Adsorption isotherms

There have been three adsorption models of Al(III), Fe(III), H_2_S, and CH_3_SH studied on andesite and HPAAsurfaces. Isotherm formulas, focusing on the physical adsorption of vapors and gases, reveal the crucial characteristics of industrial sorbents, including pore volume, pore size or specific surface area^71^, and energy distribution. Langmuir isotherm is the most basic kind of isotherm. The Langmuir equation has limitations since it assumes single-layer adsorption on the adsorbate surface without molecular attraction. Nevertheless, by applying this formula:

6 $$C_{e} / \, q_{e} = \, 1 \, / \, \left( {q_{m} K_{L} } \right) \, + \, \left( {1/q_{m} } \right) \, \times \, C_{e}$$

**Fig. 13:**
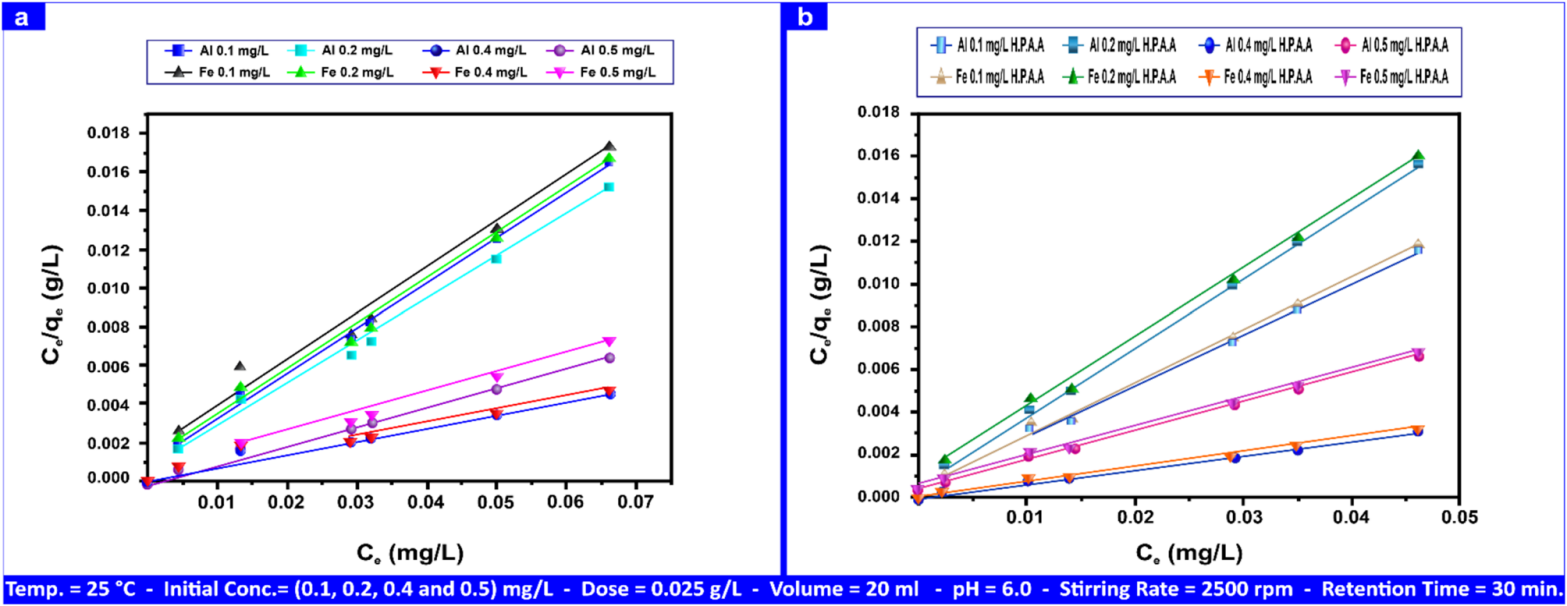
Al(III) and Fe(III) Langmuir isotherms on andesite (**a**) and HPAA composite (**b**) range from (0.1- 0.50) mg L⁻^1^.

The ratios of "q_m_" and "K_L_" for Al(III) and Fe(III), in addition to both CH_3_SH and H_2_S, may be found using the slope and intercept of the linear plot generated by the Langmuir equation, which is displayed in Fig. 13a,b and Fig. S8a,b.

To simulate multi-layer adsorption onto heterogeneous surfaces, one particular method is the Freundlich isotherm, which is explained by the following equation^72^:

7 $$q_{e} = \, K_{F} C_{e}^{\beta } = \, K_{F} C_{e}^{1/n}$$

where "q_e_" is the equilibrium quantity adsorbed per unit mass of adsorbent (mg g⁻^1^), "C_e_" is the equilibrium concentration of the adsorbate in solution (mg L⁻^1^), "K_F_" is the Freundlich isotherm constant (mg g⁻^1^), and "n" is the adsorption intensity. The logarithmic linear version of Freundlich’s equation is as follows:

8 $$Log \, q_{e} = \, \log \left( {K_{F} } \right) \, + \, \left( {1/n} \right)\left( {\log \, C_{e} } \right)$$

According to the Temkin isotherm equation, adsorption is typified by a uniform distribution of bonding energies up to a high binding energy^73^, and because of adsorbent-adsorbate interactions, every molecule in the layer experiences a linear drop in adsorption heat as coverage increases. The following^74^ is the Temkin linearised form of the equation:

9 $$q_{e} = \, B \, \ln \, A_{T} + \, B \, \ln \, C_{e}$$

"A_T_" stands for the binding constant (L mg⁻^1^), and "B" for the adsorption heat (KJ mol⁻^1^).

10 $$B \, = \, RT/b_{T}$$

"R" is a gas constant (universal) (8.314 J/K mol), "T" is the absolute temperature in Kelvin (K), and 1/ b_T_ indicates the adsorbent potential adsorption. According to certain claims, linearization graphs might not be a trustworthy foundation for approving or disapproving a model^75^. It was determined whether the three models (Freundlich, Langmuir, and Temkin) were applicable by evaluating how well they adapted to the experimental data. The data fitness was determined using one statistical measure (R^2^), known as the determination coefficient.

Table 4 displays the determination coefficient values for each of the three models, not all of which are appropriate for describing the data. R^2^, "q_m_", and "K_L_" values for Al(III) and Fe(III) ranged from (0.981–0.994), (4.18–18.15) mg g⁻^1^, and (0.23–0.38) L mg⁻^1^, to (0.959–0.988), (4.13–16.28) mg g⁻^1^, and (0.16–0.30) L mg⁻^1^, respectively. For H_2_S (0.975, 163.39) mg g⁻^1^, and 0.24 L mg⁻^1^, whereas those for CH_3_SH (0.978, 10.49) mg g⁻^1^, and 2.23 L mg⁻^1^.

Table S1 shows all of the descriptions data derived from the process of adsorption utilizing HPAA compositefor the three models (Freundlich, Langmuir, and Temkin). The Langmuir parameters "R^2^", "q_m_", and "K_L_" for Al(III) and Fe(III) were (0.995–0.998), (4.08–18.68) (mg g⁻^1^), and (0.60–1.14) L mg⁻^1^, and (0.993–0.997), (4.03–18.41) (mg g⁻^1^), and (0.5–0.78) L mg⁻^1^. The values for H_2_S were (0.951, 111.11) mg g⁻^1l^, and 3.23 L mg⁻^1^, whereas those for CH_3_SH were (0.996, 14.83) mg g⁻^1^, and 7.39 L mg⁻^1^, respectively. Higher "K_L_" values indicate a higher affinity of the adsorbent for the metal. The interaction of all variables significant to the adsorption isotherms revealed that the adsorption of Al(III), Fe(III), H_2_S, and CH_3_SH on andesite and HPAA followed the Langmuir the linear form adsorption equation, as seen in Fig. 13a,b, and Fig. S8a,b.

**Fig. 14:**
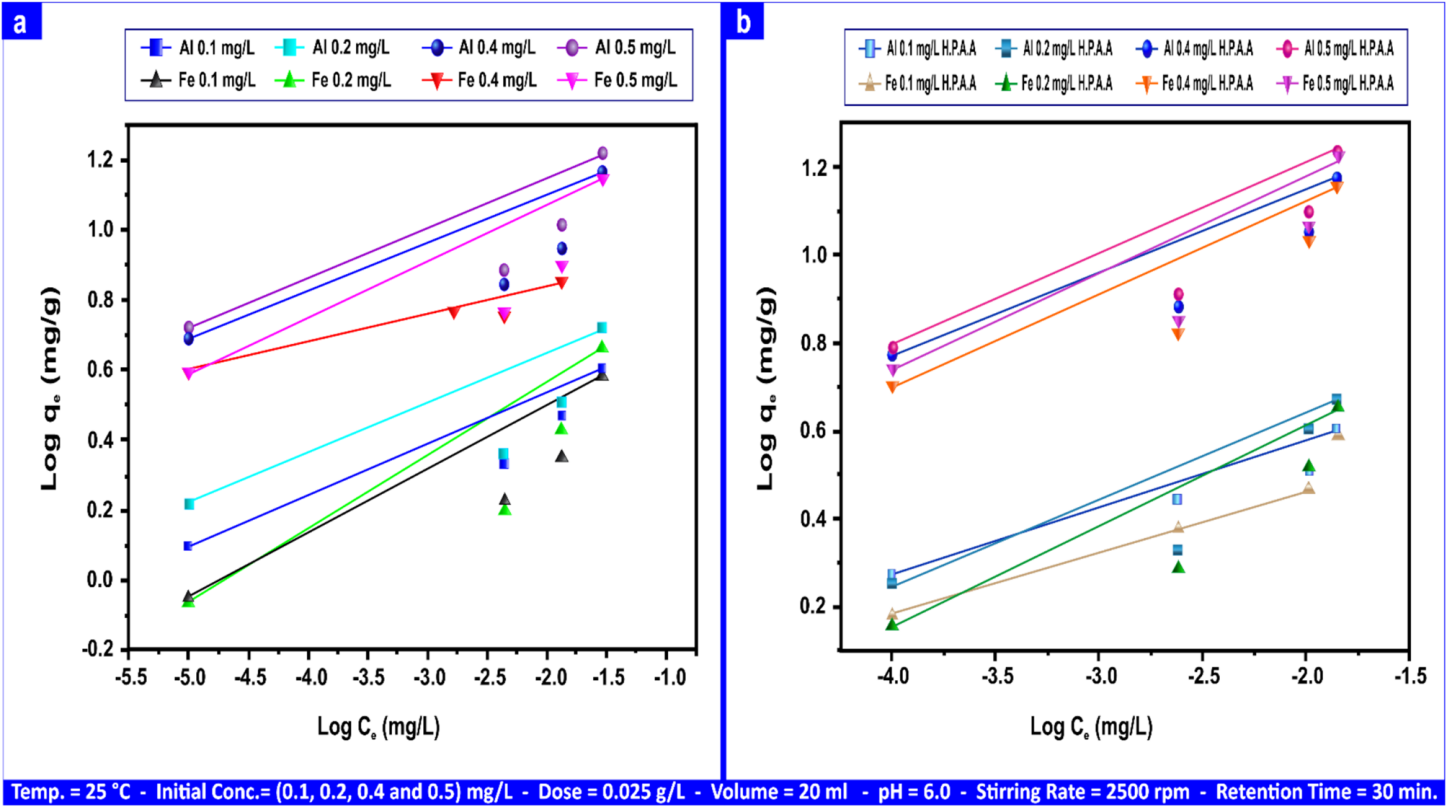
Al(III) and Fe(III) Freundlich isotherms on andesite (**a**) and HPAA composite (**b**) range from (0.1- 0.50) mg L⁻^1^.

Figure 14a presents the Freundlich mg L⁻^1^ linear forms of Al(III) and Fe(III) with andesite. Freundlich parameters ("R^2^", "n", "K_F_") for Al(III) and Fe(III) were (0.731–0.980), (6.25–8.70), and (5.31–19.00) mg g⁻^1^, respectively; (0.664–0.857), (5.12–7.51), and (5.00–16.40) mg g⁻^1^. Figure 14b shows the Freundlich linear forms of Al(III) and Fe(III) utilizing HPAA. The Freundlich parameters ("R^2^", "n", and "K_F_") for Al(III) and Fe(III) were (0.801–0.955), (6.00–8.12), and (6.68–27.00) mg g⁻^1^, respectively.

Figure S9a indicates that the Freundlich values ("R^2^", "n", "K_F_") for H_2_S and CH_3_SH on the surface of andesite, as an adsorbent, were (0.995, 0.993), (1.34, 1.22), and (29.9, 9.9) mg g⁻^1^, respectively. A value of "n" larger than one indicates advantageous adsorption, where the amount adsorbed increases in proportion to the solute concentration.

HPAAwas utilized as an adsorbent in Freundlich isotherm, as indicated in Fig. S9b, and the values ("R^2^", "n", "K_F_") for H_2_S and CH_3_SH were (0.974, 0.989), (2.43, 1.58), and (76.8, 24.8) (mg g⁻^1^), respectively. While H_2_S and CH_3_SH adhered to the Freundlich adsorption equation in linear form, Al(III), Fe(III), and HPAAdid not.

Both a steady rise in the amount of solute adsorbed and an increase in solute concentration are linked to less suitable adsorption, which is represented with a value of "n" below 1. In addition, the quantity adsorbed and the concentration of the adsorbate have a linear relationship when "n" = 1. The Freundlich sorption isotherm model has an "n" value of 1 < n < 10, implying advantageous sorption behavior^76^. The n value may reflect sorption favorability, with an "n" value of 1 to 10 indicating favorable sorption. Assuming multilayer adsorption on the surface, limitless surface coverage is mathematically expected since the Freundlich isotherm cannot forecast when the adsorbate^77^ will reach sorbent saturation.

**Fig. 15:**
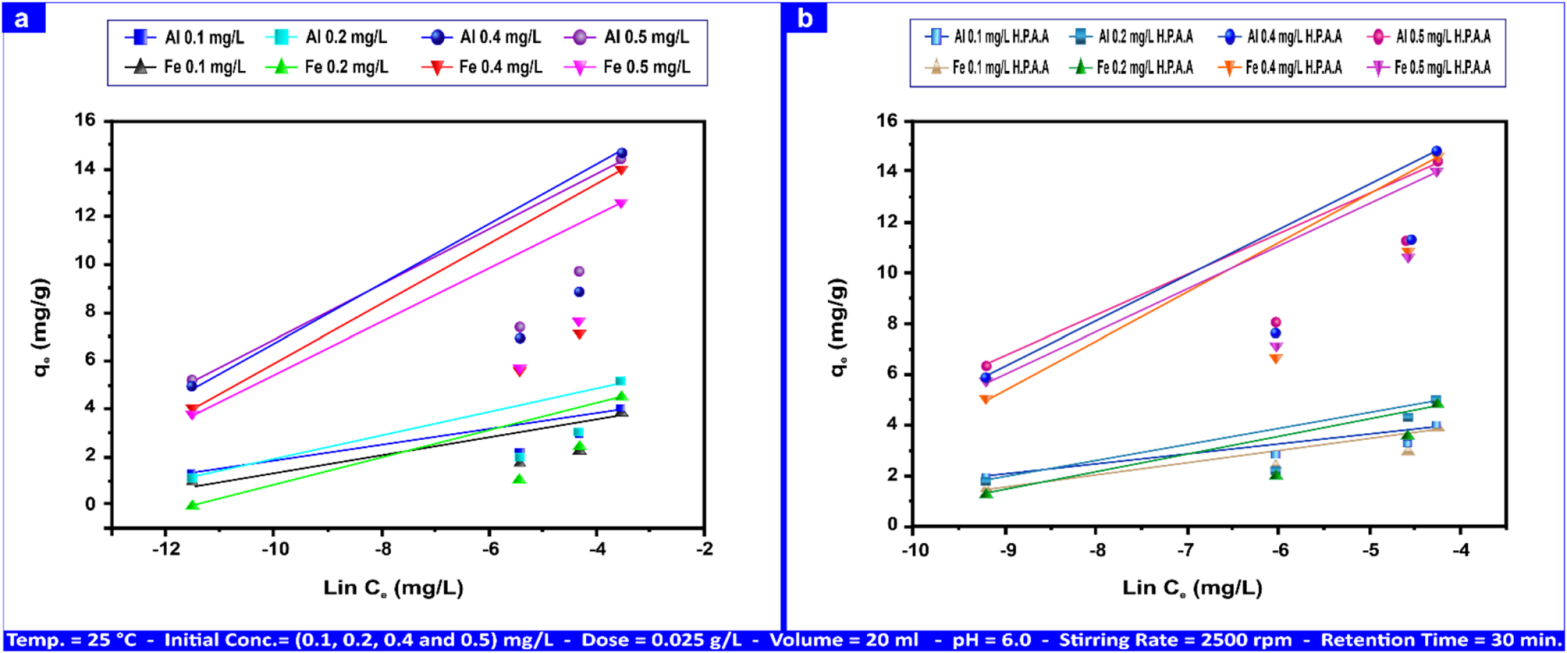
Al(III) and Fe(III) Temkin isotherms on andesite (**a**) and HPAA composite (**b**) range from (0.1- 0.50) mg L⁻^1^.

Temkin values ("R^2^", "A_T_", "b_T_") for Al(III) ranged from (0.583–0.775), (0.116–0.372) L mg⁻^1^, and (2.64–8.42) kJ mol⁻^1^; for Fe(III), they varied from (0.499–0.663), (0.114–0.342) L mg⁻^1^, and (2.87–8.60) kJ mol⁻^1^on the andesite surface. The values for Al(III) and Fe(III) utilizing HPAA Compositewere, respectively, (0.756–0.900, (0.14–0.61) L mg⁻^1^, (1.59–6.57) kJ mol⁻^1^, (0.731–0.853), (0.16–0.621) L mg⁻^1^, and (1.51–6.04) kJ mol⁻^1^, as illustrated in Fig. 15a,b.

Temkin values for H_2_S and CH_3_SH on andesite surfaces ("R^2^", "A_T_", "b_T_") were (0.961, 0.978), (0.003, 0.017) L mg⁻^1^, and (0.078, 0.90) kJ mol⁻^1^, respectively. Figure S10a,b shows composite values of (0.955, 0.983), (0.02, 0.08) L mg⁻^1^, and (0.09, 0.80) kJ mol⁻^1^. The Temkin adsorption potentials ("A_T_") of andesite for Al(III), Fe(III), H_2_S, and CH_3_SH were (0.372, 0.342, 0.003, and 0.0117) L mg⁻^1^, while those of HPAA Compositewas (0.61, 0.62, 0.02, and 0.08) L mg⁻^1^, showing a reduced kinetic potential for H_2_S.

The Temkin constant, "b_T_", of andesite about the heat of sorption for Al(III), Fe(III), H_2_S, and CH_3_SH were (8.42, 8.60, 0.078, and 0.90) kJ mol⁻^1^, and of HPAA compositewas (6.57, 6.04, 0.09, and 0.80) kJ mol⁻^1^, accordingly. According to research^78^, the ion exchange process typically requires a bonding energy of 8–16 kJ mol⁻^1^.

In contrast to H_2_S and CH_3_SH, the adsorption of Al(III) and Fe(III) on andesite and HPAA did not follow the linear form of the Temkin adsorption equation. The study’s poor findings suggest an ion exchange mechanism for H_2_S and CH_3_SH by indicating a weak contact between the sorbent and sorbate. Tables 5 and S1 contain data on the parameters of the three adsorption models.

**Table 5:** Adsorption isotherm for Al(III), Fe(III), H_2_S, and CH_3_SH andesite´s Langmuir, Freundlich, and Temkin parameters.

		AL(III) (mg L⁻^1^)				Fe(III) (mg L⁻^1^)				H_2_S (mg L⁻^1^)	CH_3_SH (mg L⁻^1^)
Isotherms	Parameters	0.1	0.2	0.4	0.5	0.1	0.2	0.4	0.5	100	10
Langmuir	q_m_ (mg g⁻^1^)	4.18	7.84	15.81	18.15	4.13	7.28	15.48	16.28	163.39	10.49
	K_L_ (L mg⁻^1^)	0.38	0.35	0.23	0.38	0.21	0.27	0.16	0.30	0.24	2.23
	R^2^	0.994	0.992	0.981	0.993	0.978	0.988	0.959	0.988	0.975	0.978
Freundlich	K_F_ (mg g⁻^1^)	5.31	8.10	16.10	19.00	5.00	8.50	14.00	16.40	29.90	9.90
	N	6.25	8.70	7.19	8.69	5.12	5.84	5.92	7.51	1.43	1.22
	R^2^	0.980	0.731	0.733	0.765	0.857	0.818	0.664	0.736	0.995	0.993
Temkin	A_T_ (L mg⁻^1^)	0.116	0.166	0.353	0.372	0.114	0.194	0.339	0.342	0.003	0.017
	b_T_ (kj mol⁻^1^)	8.42	5.89	2.78	2.64	8.60	5.04	2.89	2.87	0.078	0.903
	R^2^	0.775	0.622	0.583	0.655	0.663	0.670	0.499	0.610	0.961	0.978

#### Kinetics of adsorption

The majority of the transformation processes of sorption and desorption of different solid phases are reliant on time. Understanding the kinetics of these processes is crucial^79^ to forecast the fate of pollutants over time and to comprehend their dynamic interactions with solid phases. The kinetic adsorption process consists of two phases. It is presumed that during the first stage, the adsorbate was moved from the main solution to the adsorbent site. The next procedure therefore disperses and arranges the adsorbent within the absorbent pores.

Another part of describing the adsorption mechanism^79^ is figuring out how quickly the adsorption process takes place. It presents the hypothesis that the adsorption percentage constant results from a first-order kinetic process.

11 $$dq_{t} /d_{t} = \, k_{1} \left( {q_{e} - \, q_{t} } \right)$$

For first-order adsorption, "k_1_" stands for the adsorption rate constant; "q_e_" and "q_t_" indicate the quantity of substrate adsorbed at saturation (mg g⁻^1^) and time (t) (mg g⁻^1^), respectively. The next expression results from the integration of Eq. (8):

12 $$Ln\left( {q_{e} - \, q_{t} } \right) \, = \, Ln\left( {q_{e} } \right) \, - \, k_{1} t$$

The slope and intercept of the linear graph of Ln(q_e_—q_t_) versus "t" correspond to the values of "k_1_" and Ln(q_e_), accordingly. By comparing the "q_e_" values derived from the plot crossings and actual data^80^, it is evident that the adsorption process follows first-order kinetics.

**Fig. 16:**
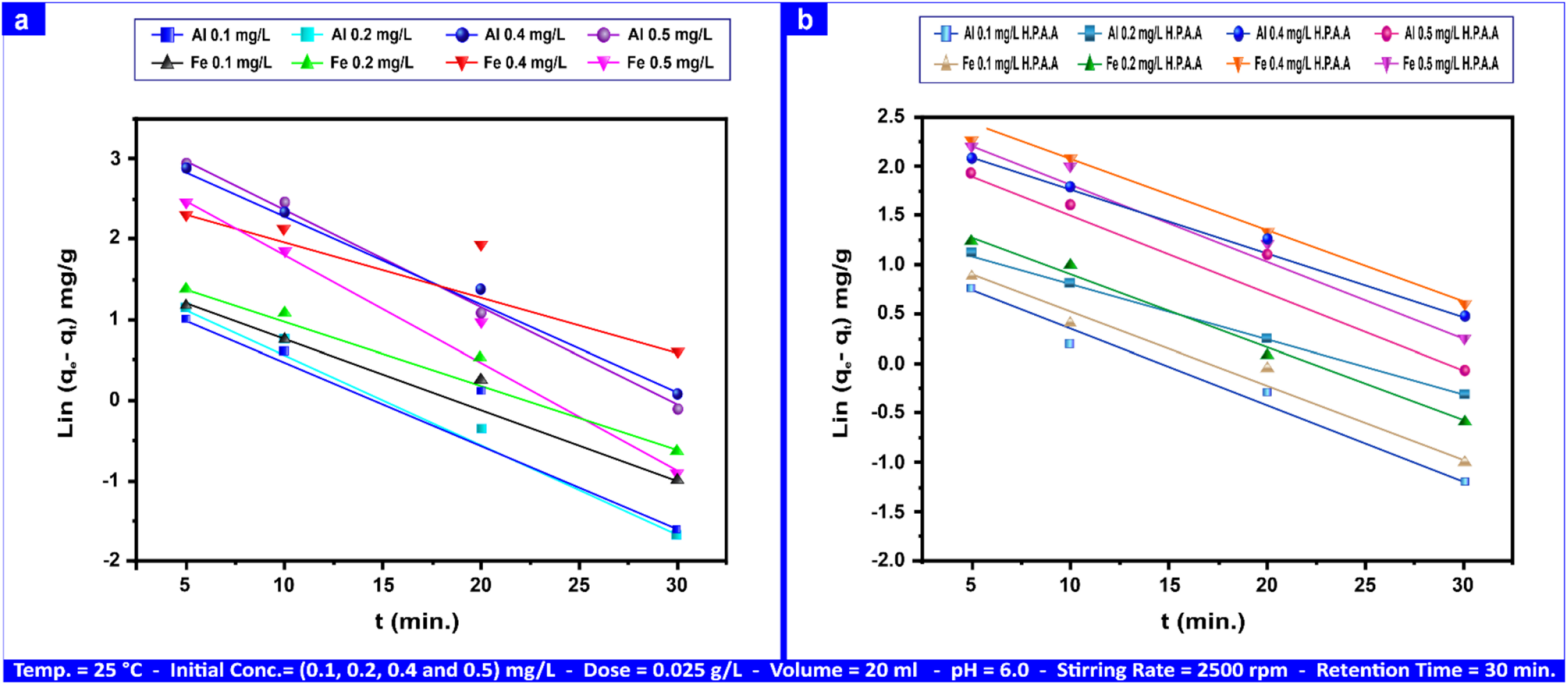
First-order kinetical curves of Al(III) and Fe(III) adsorption by andesite (**a**) and HPAA composite (**b**) range from (0.1- 0.50) mg L⁻^1^.

The adsorption kinetic curves shown in Figs. 16a,b, S11a,b, 17a,b, and S12a,b are for Al(III) and Fe(III), which range from 0.1 to 0.50 mg L⁻^1^, as well as for H_2_S and CH_3_SH, which range from 20 to 100 mg L⁻^1^ and 2–10 mg L⁻^1^, correspondingly. Al(III) and Fe(III) needed 30 min to reach equilibrium, whereas H_2_S and CH_3_SH did so in 10 min. Tables 6 and S2 present the correlation coefficient "R^2^" along with the calculated and summarized values of "k_1_", "k_2_", and "q_e_".

**Table 6:** Adsorption rate constant for first-order and second-order on andesite as an adsorbent range from (0.1- 0.50) mg L⁻^1^ for Al(III) and Fe(III), (20–100) mg L⁻^1^ and (2–10) mg L⁻^1^ for H_2_S and CH_3_SH.

		AL(III) (mg L⁻^1^)				Fe(III) (mg L⁻^1^)				H_2_S (mg L⁻^1^)	CH_3_SH (mg L⁻^1^)
Kinetic model	Parameters	0.1	0.2	0.4	0.5	0.1	0.2	0.4	0.5	100	10
Pseudo-first-order	q_e_ (mg g⁻^1^)	5.23	9.55	28.08	35.12	26.10	55.96	105.71	112.53	604.67	14.57
	k_1_ (min⁻^1^)	0.10	0.10	0.12	0.15	0.25	0.28	0.26	0.28	0.80	0.41
	R^2^	0.938	0.836	0.797	0.807	0.752	0.763	0.723	0.742	0.921	0.976
Pseudo-second-order	q_e_ (mg g⁻^1^)	6.15	9.48	18.67	22.66	6.48	12.90	13.82	17.86	175.43	16.90
	K_2_ × 10^–3^ (g mg⁻^1^ min⁻^1^)	8.27	8.66	3.25	3.59	4.91	2.55	5.08	4.83	0.63	0.65
	R^2^	0.999	0.998	0.992	0.998	0.991	0.991	0.994	0.999	0.996	0.993

Applying the pseudo-second-order reaction kinetic^81^ yields the following expression:

13 $$dq_{t} /d_{t} = \, k_{2} \left( {q_{e} - \, q_{t} } \right)^{2}$$

For second-order adsorption, "k_2_" is the adsorption rate constant; "q_t_" is the quantity of adsorbed substrate (mg g⁻^1^) at the time (t); and "q_e_" is the quantity of adsorbed substrate at saturation (mg g⁻^1^). The next expression results from the integration of Eq. (9):

14 $$t/q_{t} = \, 1/k_{2} q_{e}^{2} + \, t/q_{e}$$

The graphing of t/q_t_ vs. t yields the values of "q_e_" and "k_2_". This gives both experimental and calculated values for "q_e_" along with a straight line.

The first-order parameters ("R^2^", "K_1_", "q_e_") for Al(III) and Fe(III) measured with andesite ranged from (0.797–0.938), (0.1–0.15) min⁻^1^, (5.23–35.12) mg g⁻^1^, (0.723–0.763), (0.25–0.28) min⁻^1^, and (26.10–112.53) mg g⁻^1^, respectively.

The parameters for HPAA composite were ((0.758–0.846), (0.28–0.36)) min⁻^1^, and (22.31–104.96) mg g⁻^1^ for Al(III), ((0.761–0.794), (0.24–0.36)) min⁻^1^, and ((17.94–234.86)) mg g⁻^1^for Fe(III).

The ("R^2^", "K_1_", "q_e_") values for H_2_S and CH_3_SH utilizing both adsorbents andesite and HPAA were (0.921, 0.976), (0.80, 0.41) min⁻^1^, (604.67, 14.57) mg g⁻^1^, (0.982–0.959), (0.51–0.52) min⁻^1^, and (190.38, 22.56) mg g⁻^1^.

According to a previous study, as shown in Figs. 16a,b and S11a,b, the sorption of Al(III), Fe(III), H_2_S, and CH_3_SH by andesite and HPAAwas not as well predicted by the pseudo-first-order kinetic model.

**Fig. 17:**
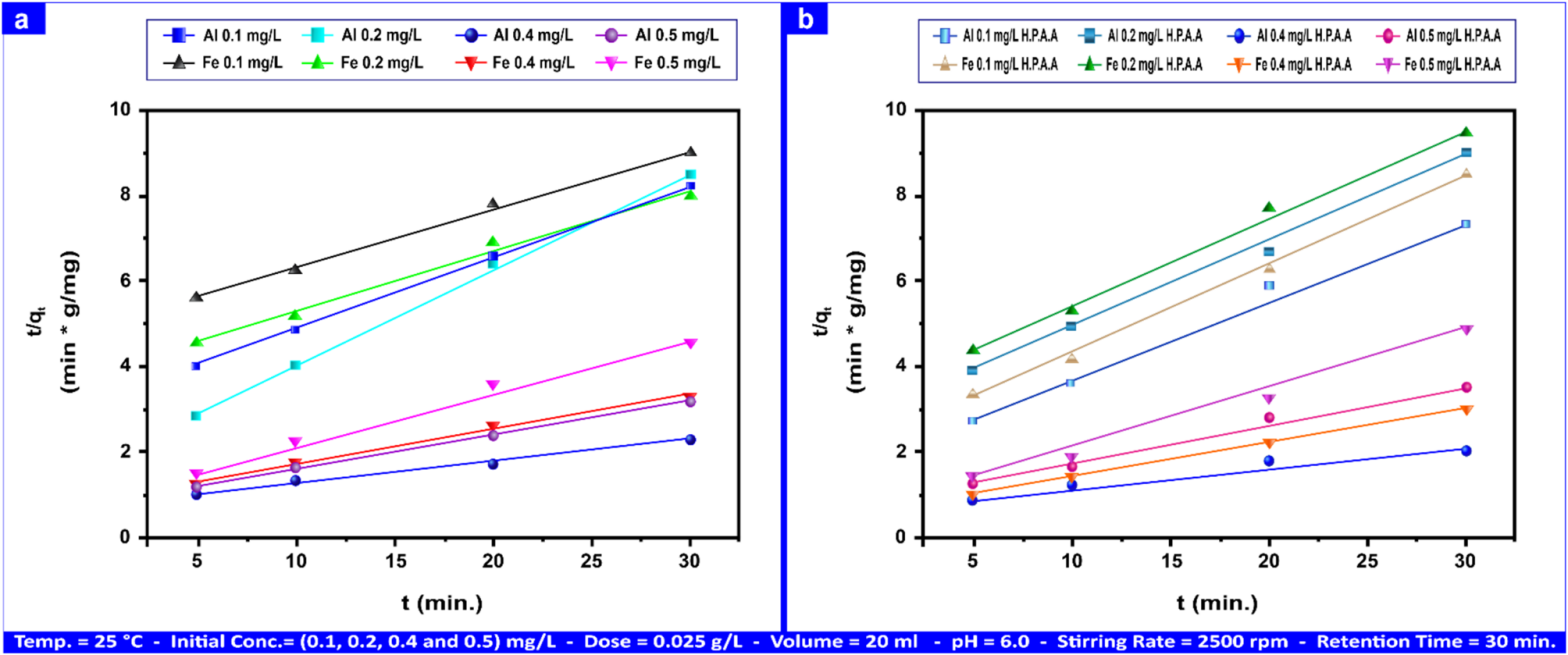
Second-order kinetical curves of Al(III) and Fe(III) adsorption by andesite (**a**) and HPAA composite (**b**) range from (0.1- 0.50) mg L⁻^1^.

Figures 17a,b and S12a,b provide a summary of the ("R^2^", "k_2_", "q_e_") values, as do Tables 6, S2. It is more accurate to estimate the sorption of Al(III), Fe(III), H_2_S, and CH_3_SH by andesite and HPAA using the pseudo-second-order kinetic model; for Al(III) and Fe(III) ranged from (0.992–0.999), (3.25–8.27) (g mg⁻^1^ min⁻^1^), (6.15–22.66) mg g⁻^1^, (0.991–0.999), (2.55–5.08)) (g mg⁻^1^ min⁻^1^) and (6.48–17.86) mg g⁻^1^ respectively on andesite surface, and by composite, parameters were (0.0938–0.995), (0.002–0.02) (g mg⁻^1^), (5.20–23.05) mg g⁻^1^, (0.949–0.996), (0.008–0.02) (g mg⁻^1^ min⁻^1^) and (4.30–16.23) mg g⁻^1^.

Using pseudo-second-order adsorption, the values for H_2_S and CH_3_SH on andesite were (0.996, 0.993), (0.63–0.65) (g mg⁻^1^ min⁻^1^), and (175.43–16.90) mg g⁻^1^, respectively. The values for HPAA were (0.999, 0.997), (0.001, 0.01) (g mg⁻^1^ min⁻^1^), and (144.09, 15.08) mg g⁻^1^.

Using the following equation, Weber and Morris proposed the intra-particle diffusion model:

15 $$Q_{t} = \, K_{pi} t^{1/2} + \, C$$

**Fig. 18:**
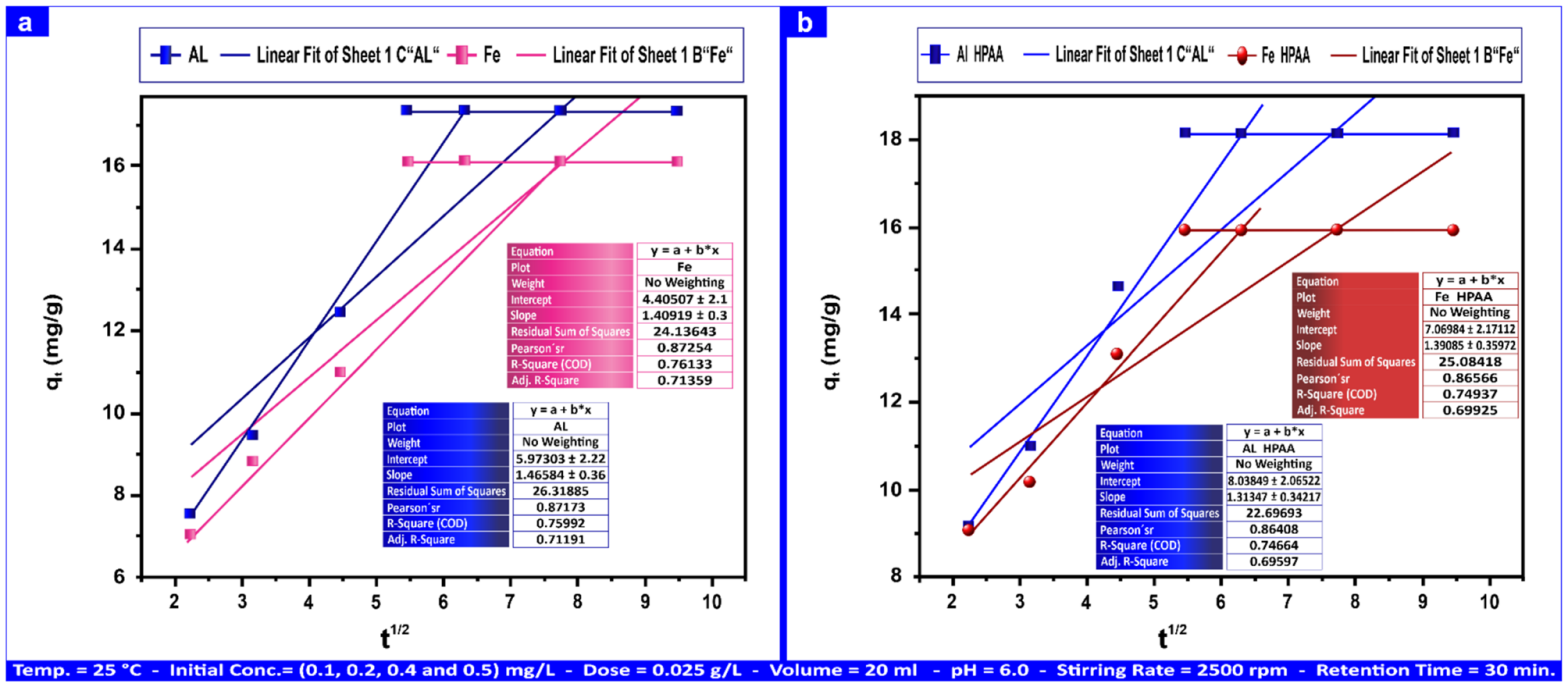
Intra-particle diffusion model for adsorption of Al(III) and Fe(III) on andesite (**a**) and HPAA composite (**b**) as an absorbent at 0.50 mg L⁻^1^.

**Fig. 19:**
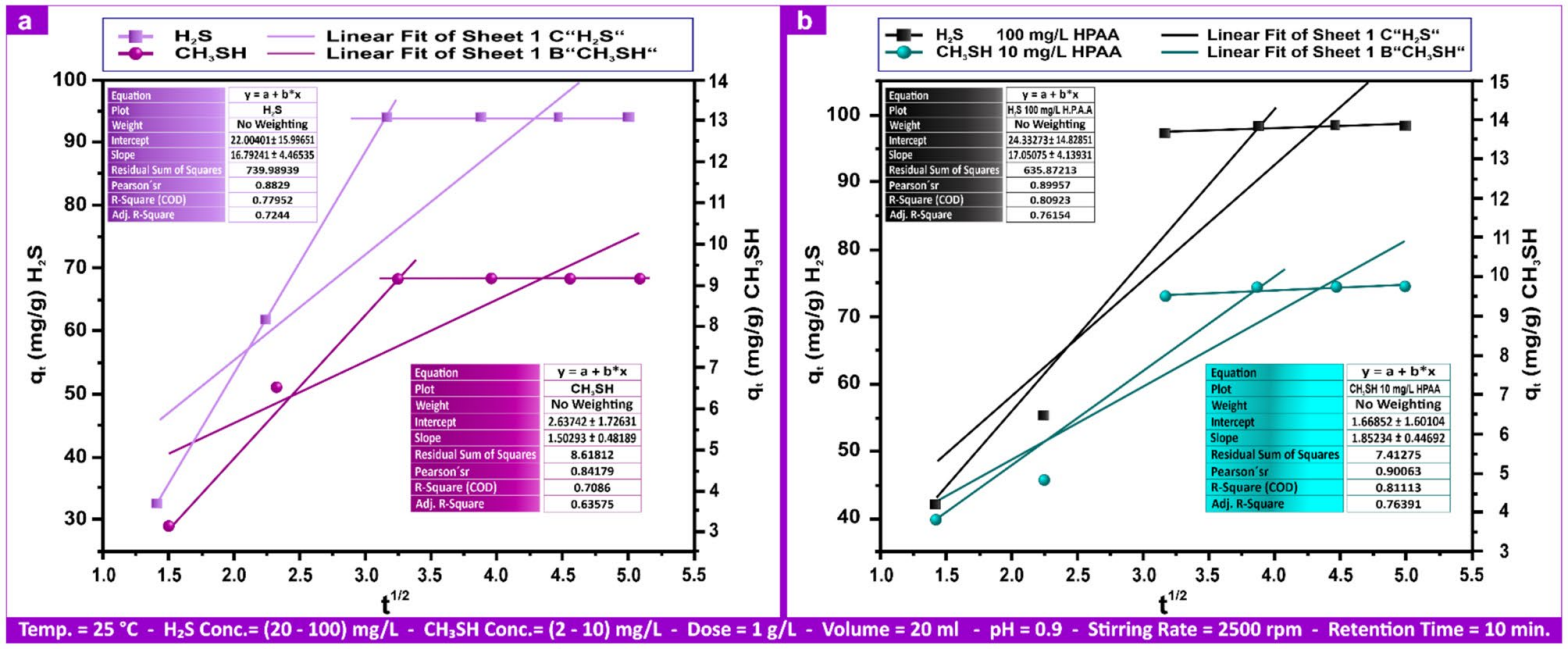
Intra-particle diffusion model for adsorption of H_2_S and CH_3_SH adsorption by andesite (**a**) and HPAA composite (**b**) range from (20–100) mg L⁻^1^ and (2–10) mg L⁻^1^.

The intra-particle diffusion rate constant is denoted by "K_pi_" (mg g⁻^1^ min⁻^1/2^), whereas the thickness of the boundary layer is described by "C" (mg g⁻^1^). Figures show the Q_t_ vs. t^1/2^ plot. The slope "K_pi_" and an intercept "C" have a linear connection, as shown in Figs. 18a,b and 19a,b.

The following steps comprise the overarching strategy for every adsorption process:

- Metal ions and gas molecules must penetrate from the edge of the boundary zone film to the adsorbent surface by means of the adsorbent’s border zone impact.
- Intra-particle solute diffusion takes place in liquid-filled pores or adsorbed conditions, whereas pollutants penetrate the porous structure of the adsorbent^82^. Table 7 shows the "C" value, which indicates the thickness of the boundary zone; the higher the intercept, the larger the border layer’s impact. Furthermore, the straight line’s deviation from its starting point signifies that sorbate^83^ removal may entail many rate-controlling steps in addition to pore dispersion.The method consists of continuously stirring with a magnetic stirrer to rapidly diffuse Al(III), Fe(III), H_2_S, and CH_3_SH onto the andesite surface. Al(III) and Fe(III) ions may permeate the permeable andesite structure and react with hydroxyl groups until equilibrium is reached^39^. While H_2_S and CH_3_SH engage with hydroxyl groups to create hydrogen bonds, as silanol and aluminol groups deprotonate at basic pH levels over 8. As noted in Table S3, the values of the boundary layer thickness C obtained from the adsorption process on HPAA composite are bigger than in the case of andesite. As a result, adsorption in HPAA has a greater effect on the boundary layer, as Al(III) and Fe(III) ions occur in the composite’s porous structure, and H_2_S and H_3_SH can form hydrogen bonds with hydroxyl that exist in silanol, aluminium, and oxime groups in addition to the amine group. Until equilibrium is attained, they have a high chance of spreading on the surface as well as binding inside the adsorbent due to an increase in active groups.

**Table 7:** Intra-particle diffusion model values for Al(III), Fe(III), H_2_S, and CH_3_SH adsorption on andesite as an absorbent.

		AL(III) (mg L⁻^1^)	Fe(III) (mg L⁻^1^)	H_2_S (mg L⁻^1^)	CH_3_SH (mg L⁻^1^)
Kinetic models	Parameters	0.5	0.5	100	10
Intra-particle diffusion	K_pi_ (mg g⁻^1^ min^-1/2^)	1.46	14.1	16.79	1.50
	C (mg g⁻^1^)	5.79	4.40	22.00	2.63
	R^2^	0.759	0.713	0.799	0.708

#### Thermodynamic

**Fig. 20:**
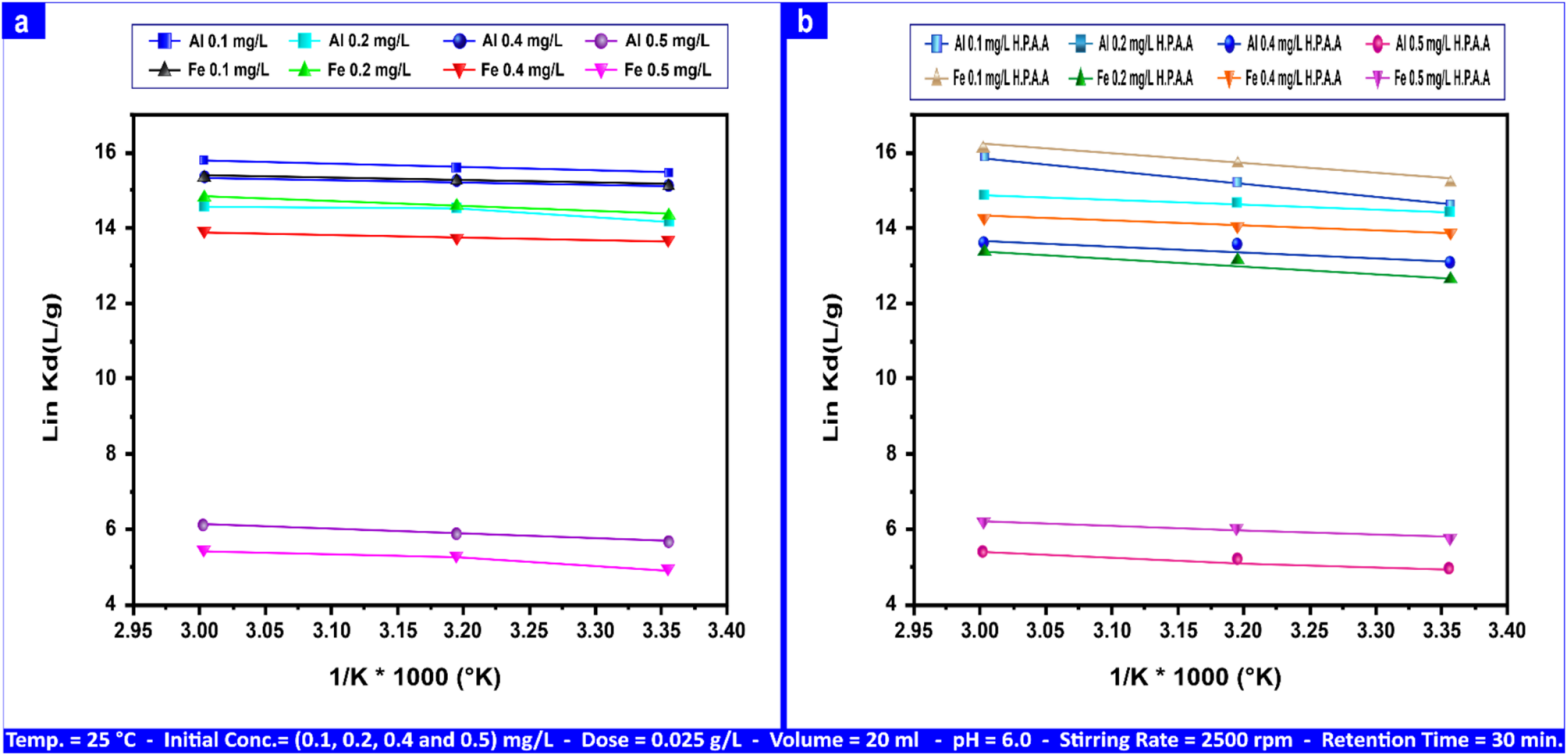
The impact of temperature on the sorption of Al(III) and Fe(III) on andesite (**a**); and the thermodynamic behavior of the HPAA composite (**b**).

Figures 20a,b and S13a,b, which represent the ln "K_d_" slope and intercept versus 1/T, were utilized to compute the thermodynamic parameters on the surfaces of both adsorbents (andesite and HPAA composite) for Al(III), Fe(III), H_2_S, and CH_3_SH, respectively.

16 $$\ln \, K_{d} = \, \left( {\Delta S \, / \, R} \right) \, {-} \, \left( {\Delta H \, / \, R_{T} } \right)$$

"K_d_", in this context, stands for temperature (in Kelvin), enthalpy, gas constant, and entropy, and it also represents the distribution coefficient. We used a well-known equation to measure the Gibbs free energy (ΔG) of the particular sorption.

17 $$\Delta G \, = \, \Delta H \, {-} \, T. \, \Delta S$$

Tables 8 and S4 detail the thermodynamic parameter values for the adsorption of Al(III) and Fe(III) onto both andesite and HPAA composite as adsorbents, within the concentration range of (0.1–0.50) mg L⁻^1^ . Furthermore, these tables also include the thermodynamic parameters for the adsorption of H_2_S and CH_3_SH within the concentration ranges of (20–100) mg L⁻^1^ and (2–10) mg L⁻^1^, respectively.

**Table 8:** Thermodynamic parameters for Al(III) and Fe(III) adsorption on andesite adsorbent range from (0.1- 0.50) mg L⁻^1^, (20–100) mg L⁻^1^, and (2–10) mg L⁻^1^ for H_2_S and CH_3_SH.

	ΔH (kJ mol⁻^1^)	ΔS (J/k/mol)	E_a_ (J/k/mol) (298–333)K	- ΔG (kJ mol⁻^1^)		
				298	313	323
Al(III (mg L⁻^1^)						
0.1	15.45	172.54	17.92	51.40	53.99	57.44
0.2	9.53	150.21	12.01	44.75	47.00	50.01
0.4	11.59	155.4	14.06	46.30	48.63	51.74
0.5	9.55	78.36	12.02	23.34	24.51	26.08
Fe(III) (mg L⁻^1^)						
0.1	18.72	184.87	21.19	55.07	57.84	61.54
0.2	17.06	168.10	19.54	50.07	52.59	55.96
0.4	10.06	145.83	12.54	43.44	45.63	48.55
0.5	12.20	82.06	14.68	24.44	25.67	27.31
H_2_S (mg L⁻^1^)						
20	16.36	85.97	18.84	25.60	26.89	28.61
40	10.80	64.22	13.28	19.12	20.09	21.37
60	5.79	46.61	8.26	13.88	14.58	15.51
80	5.40	43.92	7.87	13.08	13.74	14.62
100	3.95	36.97	6.43	11.01	11.56	12.30
CH_3_SH (mg L⁻^1^)						
2	12.54	66.54	15.02	19.81	20.81	22.14
4	11.04	59.65	13.52	17.76	18.66	19.85
6	8.95	50.17	11.42	14.94	15.69	16.69
8	14.01	66.79	16.49	19.89	20.89	22.22
10	4.19	33.23	6.66	9.89	10.39	11.06

A positive ΔH value indicates endothermic sorption, which works best at higher temperatures. According to observations, the negative Gibbs free energy increases as the temperature rises. The data suggests that spontaneous sorption does not require an induction phase. An endothermic reaction energy of adsorption may result from water removal from sorbed cations and the solid/solution interface.

As the concentrations of Al(III), Fe(III), H_2_S, and CH_3_SH increase, the value of ΔG becomes more positive. This suggests that spontaneity decreases as the quantity of ions to be absorbed grows. Additionally, a positive ΔS value indicates adsorption stability and promotes complication^65^. There is a structural change at the solid–liquid interface^83^ when ΔS is positive. Slope calculations were used to determine the energy of activation (Ea).

18 $$\Delta H \, = \, E_{a} - \, RT$$

All values obtained through the adsorption procedure on the surface of HPAA were much greater than those obtained on the surface of the andesite. This is owing to the high adsorption capability of HPAA Composite.

#### Adsorption mechanism

**Fig. 21:**
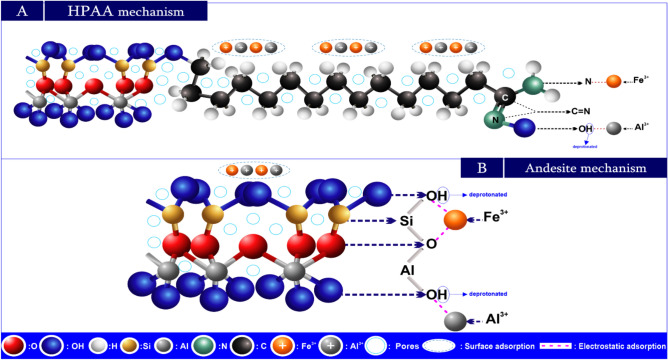
Adsorption mechanism of Al(III) and Fe(III) by HPAA (**A**) and andesite (**B**).

The adsorbate molecule M^+^ diffuses to the outside surfaces of solid matrices via the solvent or mixture. After that, it diffuses through the solid matrix’s internal pores, arrives at the active core, and employs electrostatic adsorption to adhere to the solid substance. Through chelation, the adsorbate molecule M^+^ and adsorption groups on the active site create a stable adsorption^84,85^. The adsorption probability process can be stated as shown in Fig. 21A,B.

Adsorption processes have employed andesite and modified andesite (hydrolysis polyacrylonitrile andesite HPAA composite) to eliminate metal ions Al(III) and Fe(III) as well as gases (H_2_S and CH_3_SH); consequently, every adsorbent has a distinct adsorption mechanism. The study of Al(III) and Fe(III) adsorption on andesite shows the variety of adsorption processes that can take place, including surface sorption and electrostatics. Active sites, which carry negative charges after deprotonation, are present in many of the pores on the surface of the andesite. The removal of H^+^ from charged functional groups significantly enhances surface adsorption capacity, since these areas, especially silanol (Si-OH) and aluminol (Al-OH), are electrostatically associated with the positive ions Al(III) and Fe(III).

HPAA composite use electrostatic forces and surface sorption to adsorb Al(III) and Fe(III). However, unlike andesite, HPAA composite have active groups such as oxime (C = N‒OH) and amine (NH_2_) that increase surface area and increase adsorption capacity by attracting positive metal ions to the OH^-^. The following^86^ states that the amino groups are principally in charge of the uptake of metal ions:

19 $$M^{2 + } + \, RNH_{2} + \to \, M \, \left( {RNH_{2} } \right)^{2 + }$$

**Fig. 22:**
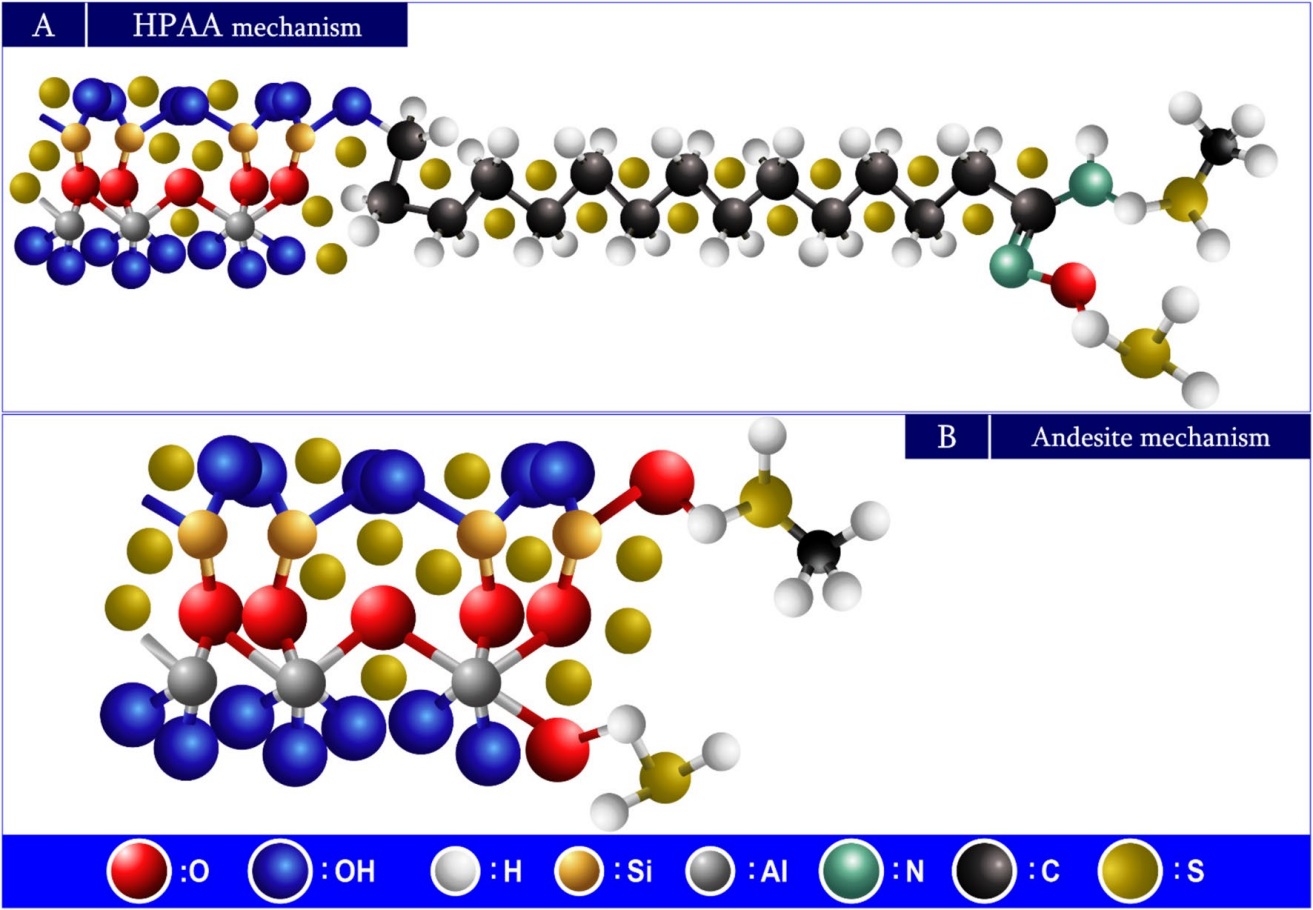
Adsorption mechanism of H_2_S and CH_3_SH by HPAA (**A**) and andesite (**B**).

Figure 22A,B illustrate the mechanisms for gas adsorption (H_2_S and CH_3_SH). Adsorption occurs as the symmetry axis of H_2_S is perpendicular to the surface^87,88^ of the andesite/HPAA composite. In this position, the S-H bond readily dissociates, allowing the resultant sulfur atom to firmly bind to the empty site.

As a consequence, the surface molecules and adsorbents (andesite and HPAA composite) form new bonds.

During dissociation, H_2_S, CH_3_SH, and S atoms easily adsorb on the surface. The single-pair electrons on their S atoms allow them to interact with the surface atoms.

The study found that HPAA composite surfaces contain active groups, including oxime (C = N‒OH) and amine (NH_2_), in addition to Al-OH and Si-OH, that were present in the andesite before modification. These groups enhance the binding of H_2_S and CH_3_SH molecules after dissociation^89^. The Comparative mechanistic Insights has been introduce in Table 9. Finally The adsorption mechanism on andesite and HPAA composites involves a nuanced interplay of diffusion, surface interaction, and chemical bonding**.** While andesite primarily relies on natural surface functionalities for ion exchange and electrostatic binding, HPAA composites demonstrate superior adsorption capacity and specificity due to their tailored chemical functionalities and enhanced surface characteristics. Experimental data including FTIR shifts, isotherm fitting, and kinetics modeling support these mechanistic conclusions.

**Table 9:** The comparative mechanistic insights for the adsorption of Al(III) , Fe(III) H2S and CH3SH by andesite and HPAA.

Aspect	Andesite	HPAA composite
Functional groups	Si–OH, Al–OH	Si–OH, Al–OH, C = NOH, NH₂
Dominant mechanism	Electrostatic, physisorption	Electrostatic, chelation, chemisorption
Adsorption sites	Natural pores, deprotonated hydroxyls	Modified pores + added functional groups
Metal ion uptake	Moderate (limited sites)	High (due to increased active group density)
Gas binding behavior	Via S–H dissociation and surface interaction	Stronger binding via multiple groups post dissociation

#### Desorption study

Previous research has been conducted on the capabilities of natural andesite in the desorption process using hydrochloric acid^90^, sodium chloride, and magnesium chloride. In this study, HPAA composite had maximum adsorption capacity and adsorption efficiency% of (18.15, 17.79) mg g⁻^1^, and (100.00, 97.58) for Al(III) and Fe(III) respectively, indicating high affinity between Al(III) and Fe(III) and HPAA composite. Therefore, the metal ions were released from the composite after 10 h and Fe(III) will released greater than Al(III) ,as the last has great affinity with adsorbent than first.

**Fig. 23:**
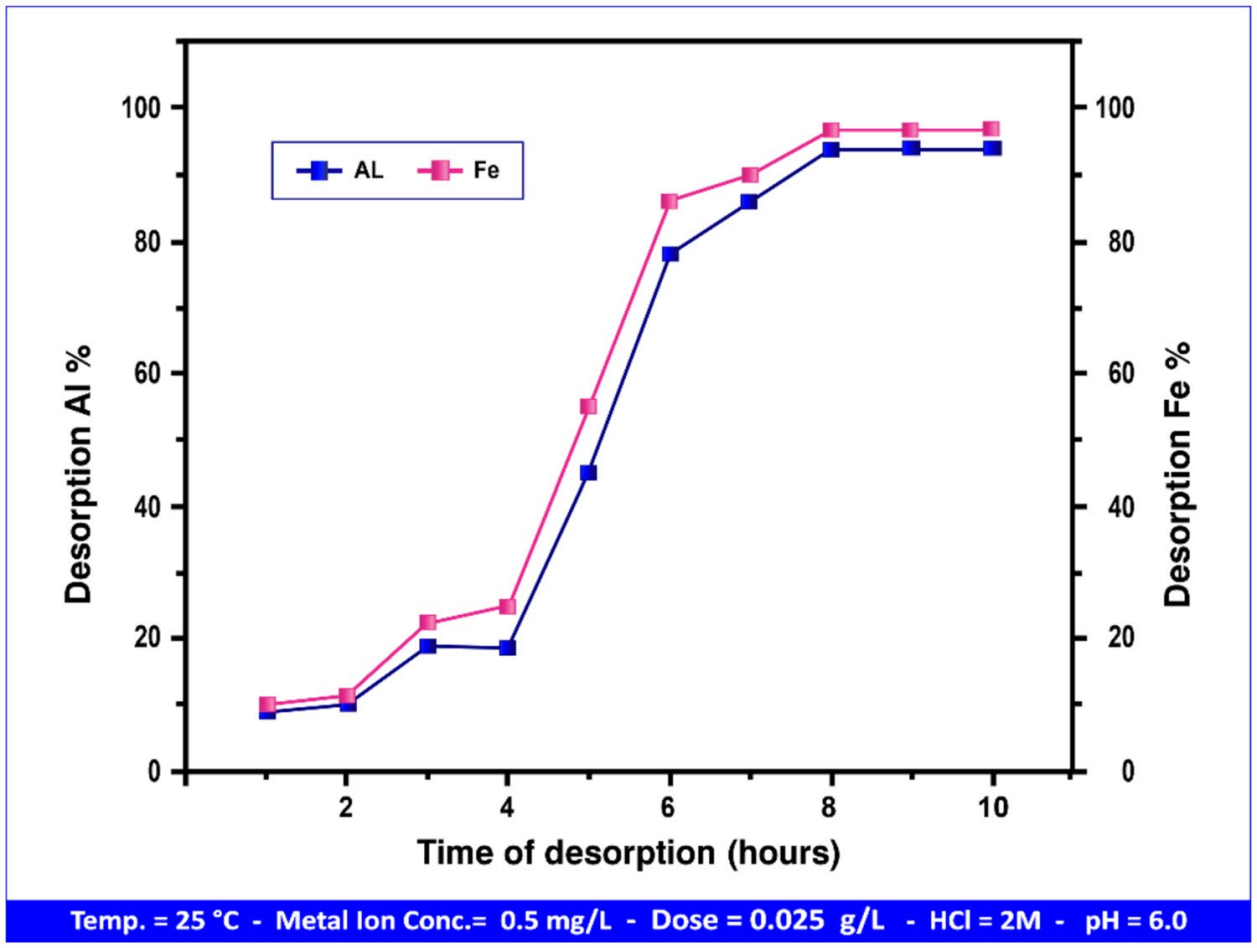
Desorption process of metal ion Al(III) and Fe(III)from andesite surface.

Figure 23, shows that the Al(III) and Fe(III) desorption percentage was initially lower. Another reason for this is that specialised locations absorbed more Al(III) and Fe(III) ions than generic sites^38^.

**Fig. 24:**
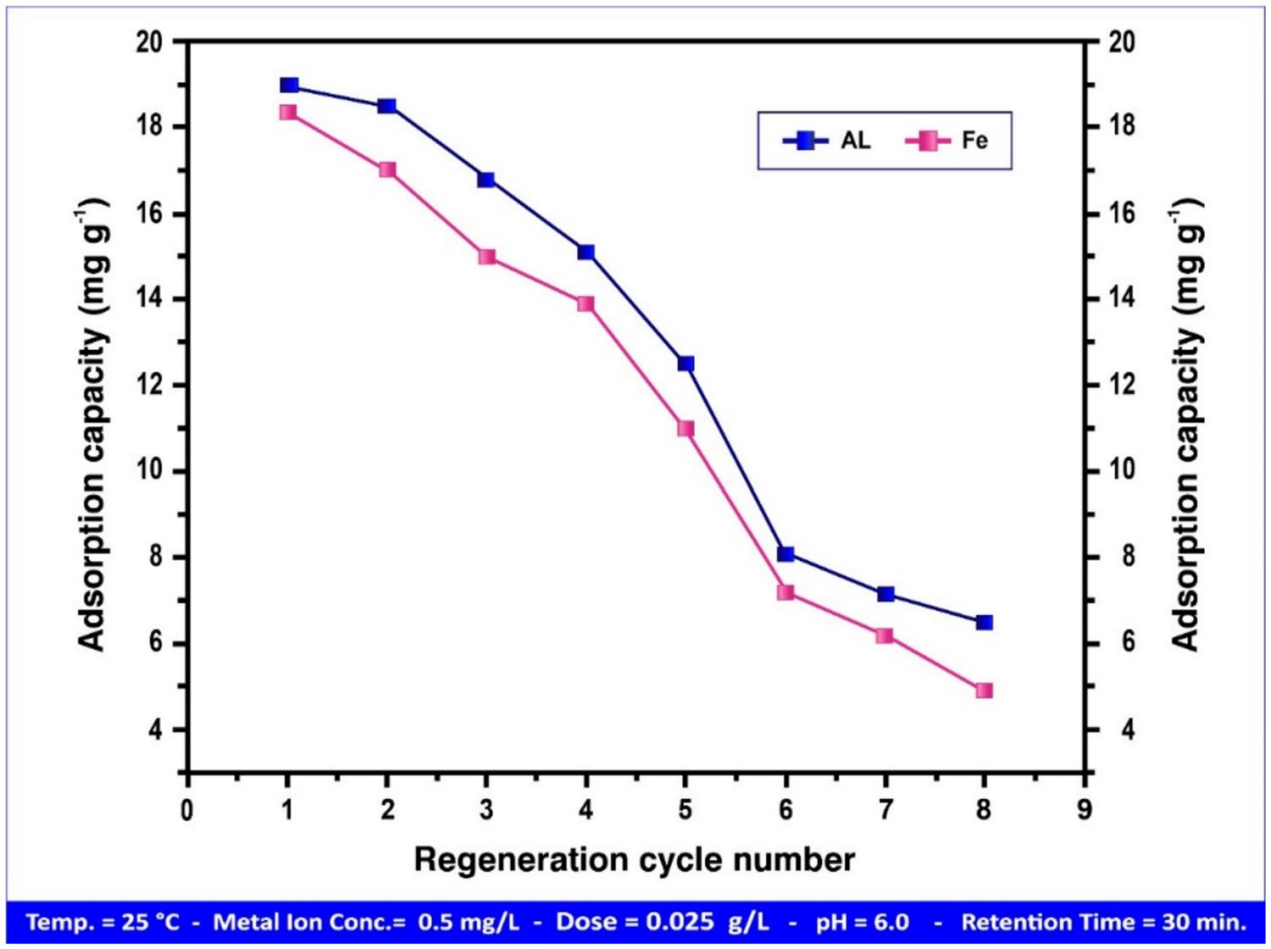
The influence of regeneration cycle numbers on the Al(III) and Fe(III) adsorption capacity.

Heavy metal ions loaded on the adsorbent can be replaced by H^+^ ions during the regeneration process, which employs HCl (2 M) to create HPAA composite, which can then be employed in additional adsorption tests. At the optimum condition for removing Al(III) and Fe(III) by HPAA composite pH, retention time, ion concentration, dosage, and temperature were 6.00, 30 min., 0.5 mg L⁻^1^, 0.025 g L⁻^1^, and 25 °C, accordingly, Fig. 24 shows how increasing the number of regeneration cycles reduces the elimination rate of the heavy metal ions indicating an inversely proportional. The adsorption capacity was (19.00, 18.36) mg g⁻^1^, for Al(III) and Fe(III), respectively in the first cycle and reached to (6.50, 4.90) mg g⁻^1^, for Al(III) and Fe(III), respectively after 8 cycles.

#### Application of HPAA composite as an adsorbent

Because of andesite’s propensity to absorb some types of contaminants from wastewater, it has been used as an adsorbent^38^. This issue could represent a serious challenge to the quality of the treated water that is used for other reasons. Six borosilicate bottles containing 200 ml of real samples were obtained from two tertiary wastewater treatment plants. Two of the bottles from plant 1, which has only one tank, were collected and acidified with 2% HNO_3_ to detect Al^3+^ and Fe^3+^, while the other sample was retained for the determination of Na^+^, NH_4_^+^, Cl^-^, Br^-^, NO_3_^-^, SO4^2-^, and K^+^, before and after adsorption process using HPAA composite under the following conditions: temperature of 25 °C, solution volume of 20 ml, contact time of 1 h, pH 6 and adsorbent dose of 0.025 g L⁻^1^, referring to Table 10, it was clear that HPAA composite has a great ability to remove Al(III) and Fe(III), NH_4_^+^, and SO_4_^2-^ from wastewater samples, and on the other side, Cl^-^, Br^+^, K^+^, and NO_3_—no influence, and Na^+^ has a slight effect, as the adsorption efficiency% for removing Al^3+^, Fe^3+^, NH_4_^+^, and SO_4_^2-^ is ranged from 99.90 to 100.00. For plant 2, four of the bottles were collected from tanks 1 and 2, as each sample was used for Hg^2+^, Al^3+^, Fe^3+^, H_2_S, and CH_3_SH analysis. All tests were carried out under the same circumstances as in plant 1 for metal ions Al^3+^, Fe^3+^, and at 25 °C, with a solution volume of 20 ml, pH 9, stirring rate of 2500 rpm, an adsorbent dose of 1 g L⁻^1^, and a retention duration of 10 min for H_2_S and CH_3_SH. Contaminants like Al^3+^, Fe^3+^, H_2_S, and CH_3_SH were effectively eliminated. Results in Table 11 prove that composite has excellent efficiency in removing Al^3+^, Fe^3+^, H_2_S, and CH_3_SH from real wastewater, as the maximum the adsorption efficiency% was 100.00, 99.80, 100.00 and 98.80 respectively, for the different two tanks related to plant 2.

**Table 10:** Real sample plant **1** during adsorption of NH_4_^+^, Cl^-^, Br^+^, NO_3**,**_ SO_4_^2-^, Na^+^, K^+^, Al^3+^, and Fe^3+^on HPAA composite as an adsorbent at conditions (dose of adsorbent 0.025 g L⁻^1^; temperature 25 °C; pH solution 6; solution volume 20 ml; and contact time 1 h).

Parameters	Before adsorption (mg L⁻^1^)	After adsorption (mg L⁻^1^)	Adsorption efficiency%	Raw tank NO
NH_4_^+^	0.50	0.0005	99.90	1
Cl^-^	245.00	240.00	2.04	1
Br^+^	10.20	10.10	0.98	1
NO_3_^-^	35.00	35.00	0.00	1
SO_4_^2 -^	182.00	0.10	99.94	1
Na^+^	98.00	98.00	0.00	1
K^+^	111.00	100.00	9.90	1
Al^3+^	0.79	0.00	100.00	1
Fe^3+^	0.39	0.00	100.00	1

**Table 11:** Real sample plant **2** during adsorption of Al^3+^, Fe^3+^, H_2_S, and CH_3_SH on HPAA composite as an adsorbent at conditions (dose of adsorbent 0.025 g L⁻^1^; temperature 25 °C; pH solution 6 ;solution volume 20 ml; and contact time 1h).

Parameters	Before adsorption (mg/L)	After adsorption (mg/L)	Uptake%	Raw tank NO
H_2_S	56.09	0.00	100.00	1
CH_3_SH	8.25	0.10	98.80	1
Al^3+^	0.81	0.00	100.00	1
Fe^3+^	0.69	0.0015	99.80	1
H_2_S	83.40	0.00	100.00	2
CH_3_SH	1.70	0.001	98.89	2
Al^3+^	0.61	0.00	100.00	2
Fe^3+^	0.79	0.002	99.75	2

At 25 °C, with a solution volume of 20 ml, pH 9, stirring rate of 2500 rpm, an adsorbent dose of 1 g L⁻^1^, and a retention duration of 10 min for H_2_S and CH_3_SH.

#### Evaluation of the performance of hydrolyzed polyacrylonitrile andesite composite

It’s essential to compare it with other common adsorbents in terms of key criteria such as adsorption capacity, selectivity, regeneration ability, cost-effectiveness, and environmental impact. As shown in Table 12**,** comparison between HPAA composite and similar adsorbents regarding to Adsorption Capacity.

**Table 12:** HPAA’s performance in comparison to similar adsorbents.

Adsorbent	Target pollutants	Adsorption capacity (mg/g)	Notes
HPAA composite	Heavy metals and gases (Al^3+^, Fe^3+^, H_2_S, CH_3_SH) ^Present study^	9.75–98.40 mg/g (varies by pollutant)	High due to synergistic effect of functional groups and porosity
Activated carbon	Organic pollutants, metals^91,92^	50–300 mg/g	Highly porous but less selective
Zeolites	Ammonium, heavy metals^93^	20–100 mg/g	Good ion exchange, lower capacity
Chitosan-based adsorbents	Metals, dyes^93,94^	80–200 mg/g	Biodegradable, but less stable
Bentonite-clay composites	Dyes, metals^95,96^	30–150 mg/g	Cheap, lower affinity for metals

Referring to Table 13, other common adsorbents in terms of key criteria such selectivity, regeneration ability, cost effectiveness, and environmental impact have been used to to highlight the advantages and limitations of HPAA.

**Table 13:** The advantages and limitations of HPAA composite compared to various adsorbents.

Criteria	HPAN-Andesite	Activated Carbon	Zeolites	Chitosan	Bentonite
1. Selectivity	Moderate-to-high for divalent/trivalent metal ions due to chelation	Poor, adsorbs a wide range of pollutants	Good, due to uniform pore size and ion exchange	High selectivity for specific metal ions	Low to moderate
2. Regeneration & reusability	Retains 70–90% capacity after 4–8 cycles with acid/base washing	Thermal regeneration possible but energy-intensive	Moderate regeneration, good chemical stability	Degrades over multiple cycles	Moderate
3. Cost & availability	Moderate cost; PAN is synthetic, andesite is abundant	Moderate to high, especially for high-grade forms	Moderate cost	High cost due to processing	Very low
4.Environmental impact & stability	Non-toxic, stable across pH range	Safe but high carbon footprint	Generally safe	Biodegradable, low toxicity	Benign

## Conclusions

The Arab Republic of Egypt’s whole desert, including the Eastern, Western, and Sinai regions, is covered in natural andesite. The following are the optimal circumstances for the adsorption of Al(III), Fe(III), H_2_S, and CH_3_SH from their aqueous solutions onto andesite and HPAA composite surfaces: The adsorption procedures for Al(III) and Fe(III) took place at 30 min, 6.00, 0.5 mg L⁻^1^, 0.025 g L⁻^1^, and 25 °C, whereas those for H_2_S and CH_3_SH took place at 10 min, 9.00, 100 mg L⁻^1^ H_2_S, 10 mg L⁻^1^ CH_3_SH, 1.00 g L⁻^1^, and 25 °C. The peak adsorption capacity and adsorption efficiency% for Al(III) and Fe(III) on andesite were (17.35, 15.39) mg g⁻^1^ and (96.00, 94.08), respectively; for H_2_S and CH_3_SH, the maximum adsorption capacity and adsorption efficiency% were (94.48, 9.08) mg g⁻^1^ and (97.50, 95.00). To increase surface area and pore size, andesite was changed utilizing the bulk method to prepare the PAA composite and hydrolysis to prepare the HPAA composite using acrylonitrile in the presence of potassium persulfate and hydroxyl amine. HPAA composite had maximum adsorption capacity and adsorption efficiency% of (18.15, 17.79) mg g⁻^1^, and (100.00, 97.58) for Al(III) and Fe(III) for H_2_S and CH_3_SH, was (98.40, 9.75) mg g⁻^1^ and (100.00, 99.00) respectively. Ideal values were used for the thermodynamic, kinetic, and isotherm investigations. Investigations were conducted into the adsorption mechanism of andesite and HPAA composite. According to the BET method, the particular surface area and average pore diameter for andesite were 1.281 m^2^/g and 92.231 nm, respectively, while for HPAA composite were 5.020 m^2^/g and 283.331 nm. On andesite and HPAA, the adsorption of Al(III), Fe(III), H_2_S, and CH_3_SH was shown to follow the linear version of the Langmuir adsorption equation. However, the adsorption of Al(III) and Fe(III) did not correspond to the linear version of the Temkin equation or the Freundlich adsorption equation. H_2_S and CH_3_SH adsorption followed the Freundlich and Temkin equations’ linear form. Furthermore, the pseudo-second-order kinetic model more accurately predicts the sorption of Al(III), Fe(III), H_2_S, and CH_3_SH by andesite and HPAA.It is feasible to reduce costs by reusing HPAA, which is fundamentally cost effective.

Andesite and the modified andesite-HPAA composite were both studied with the intra-particle diffusion model. The thermodynamic data show that the process is endothermic, with positive ΔH, ΔS, and negative ΔG. The andesite and HPAA composite adsorb spontaneously.

## Supplementary Information

Supplementary Information.

## Data Availability

All data generated or analyzed during this study are included in this published article [and its supplementary information files].
