# Supplementary material for: Application of andesite and hydrolyzed poly acrylonitrile andesite composite for adsorption of Al(III), Fe(III), CH3SH, and H2S form aqueous solutions
Source: Sci Rep. 2025 Jul 20;15:26364. doi: 10.1038/s41598-025-09497-8 (PMC12277450; doi:10.1038/s41598-025-09497-8)
Supplement: Supplementary file 1 — Supplementary Information. [file 41598_2025_9497_MOESM1_ESM.docx]

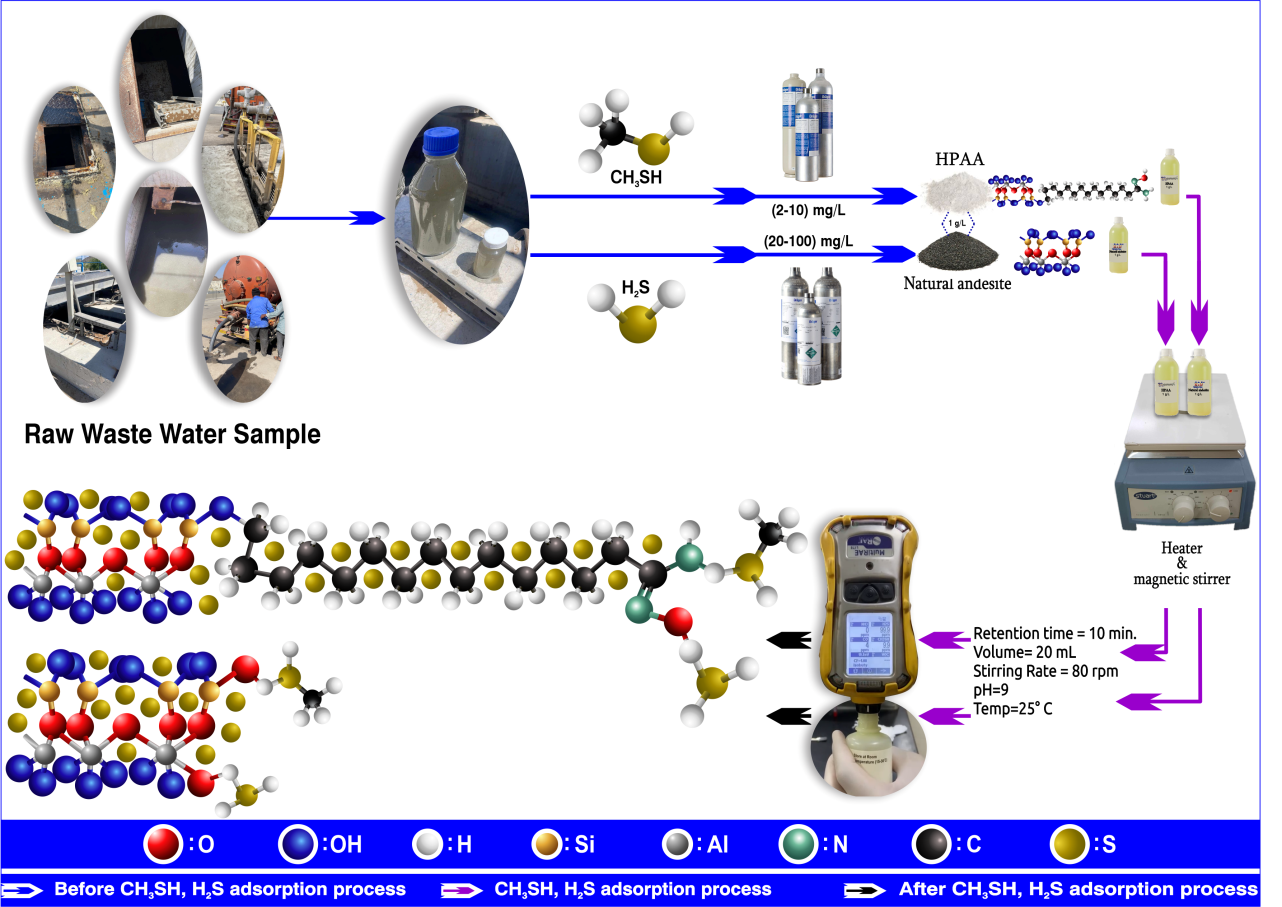

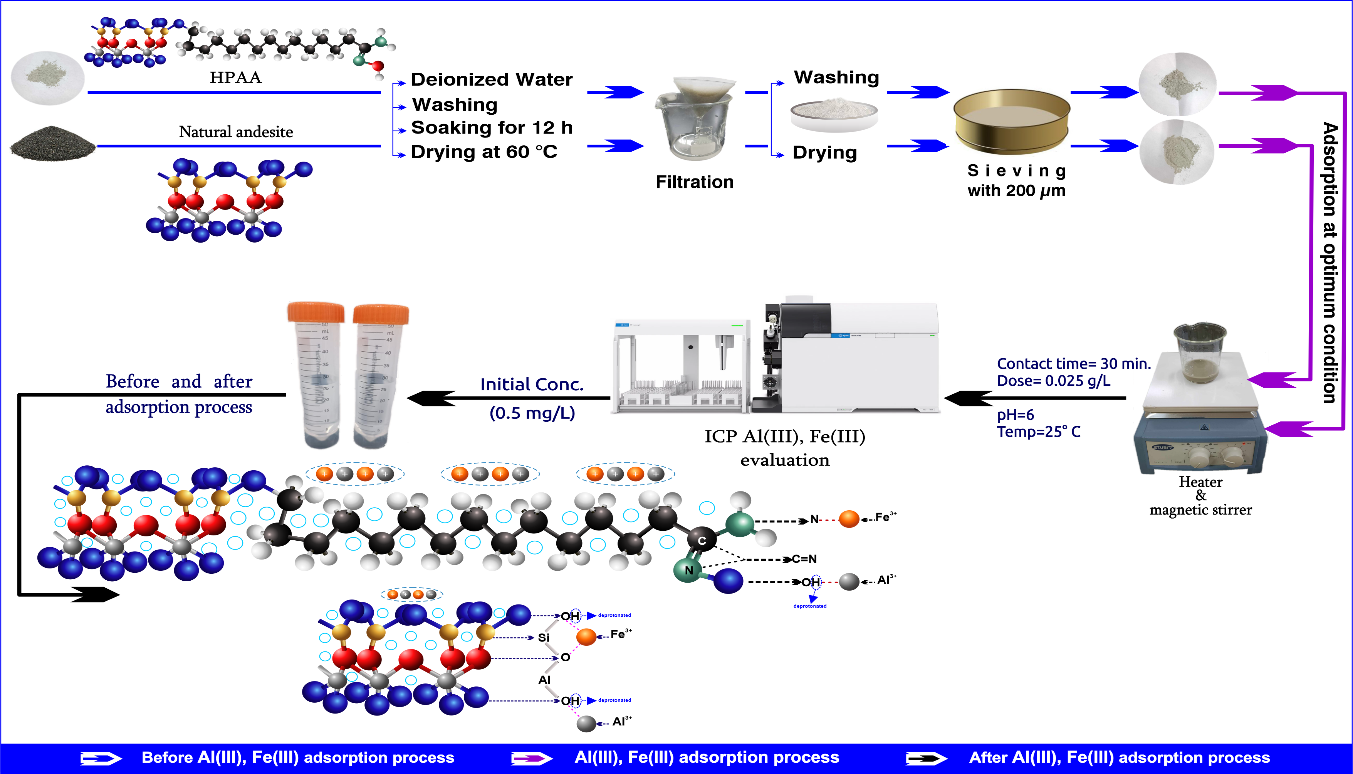
**Figure S1.** Representative diagram of Al(III) and Fe(III) adsorption processes.

**Figure S2.** Representative diagram of CH_3_SH and H_2_S adsorption processes.

**
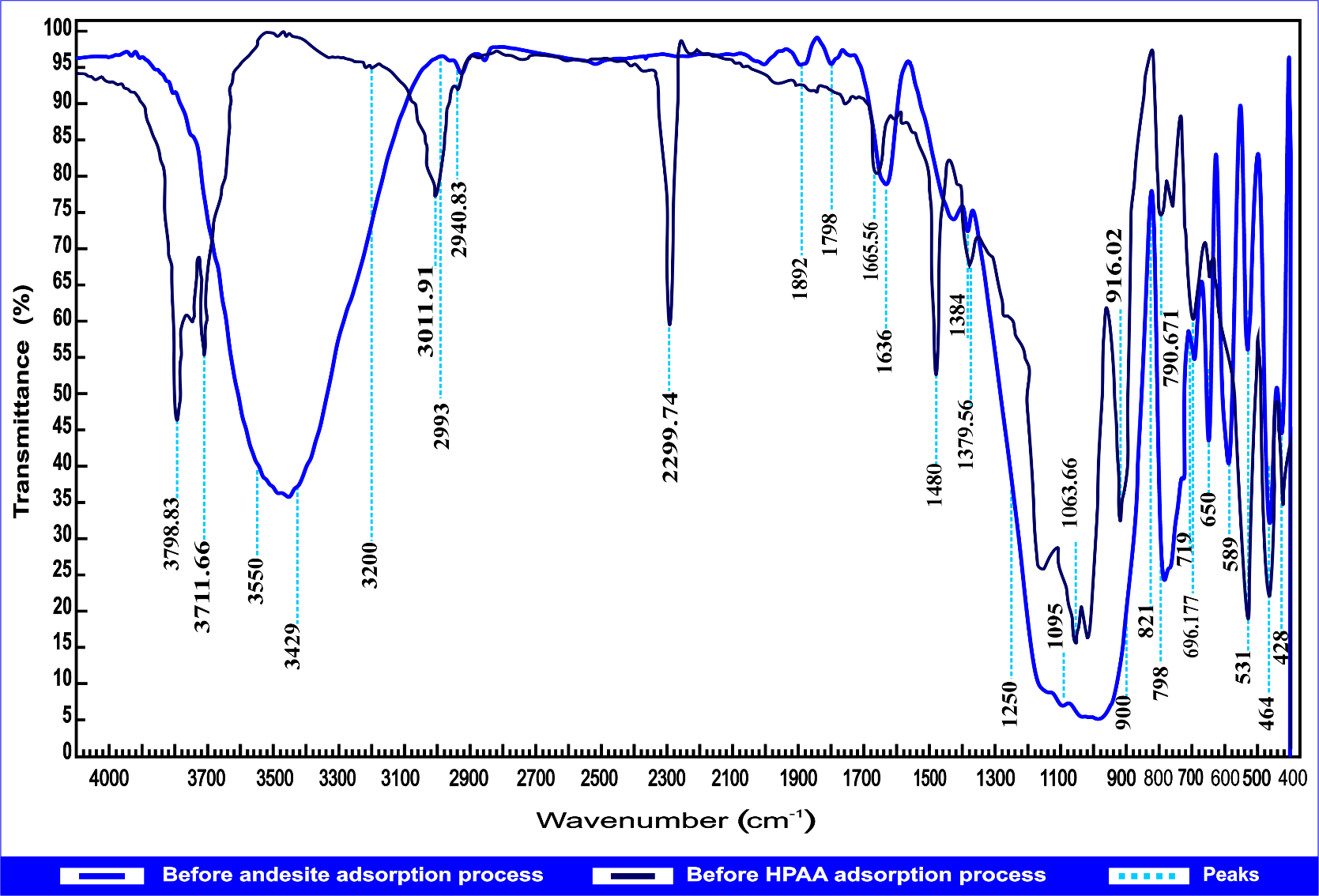
Figure S3**. FTIR spectrophotometer analysis of andesite and HPAA before adsorption process.

**
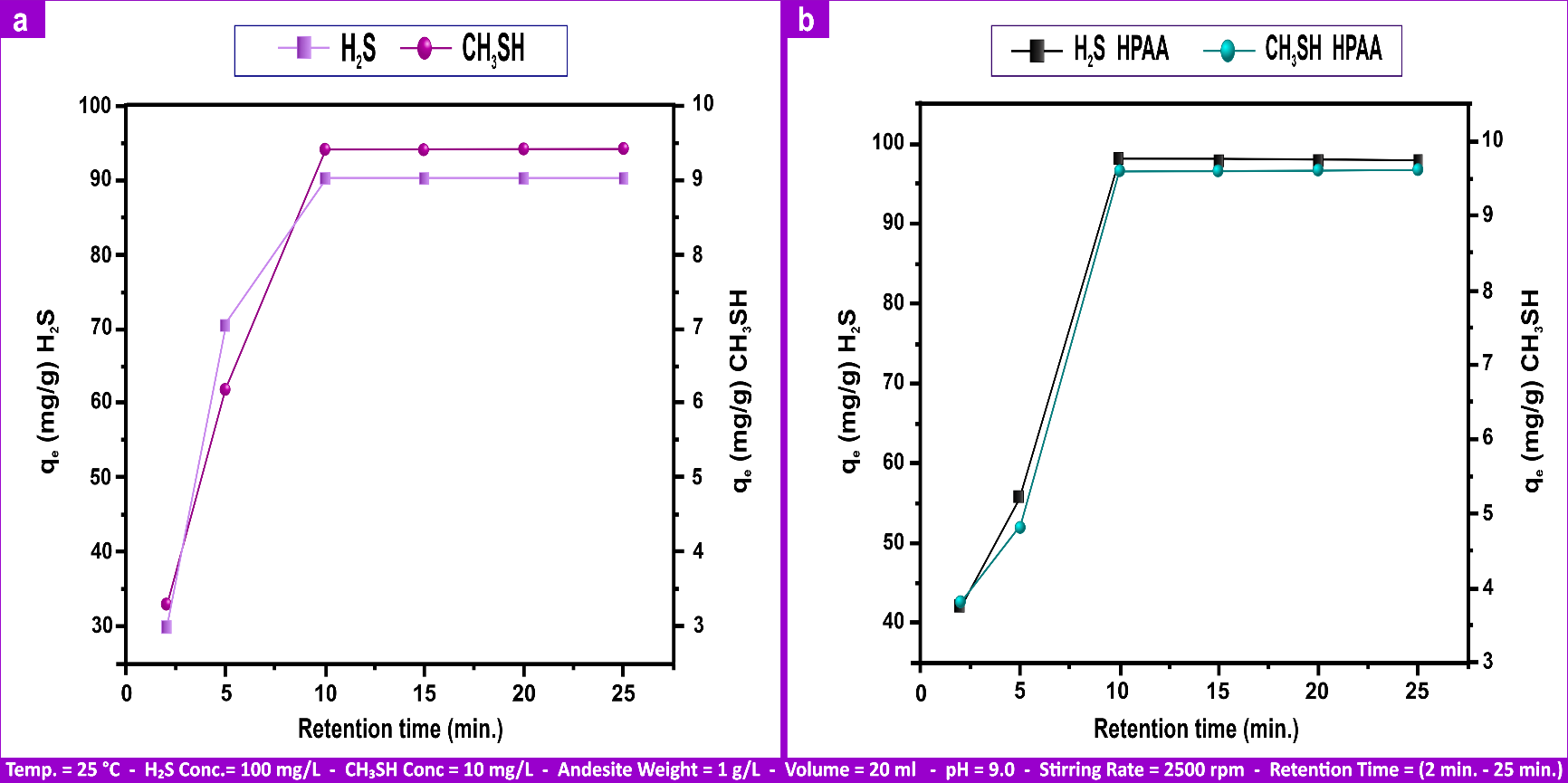
**

**Figure S4.** Retention time influence on H_2_S and CH_3_SH adsorption on andesite **(a)** and HPAA composite **(b)**.


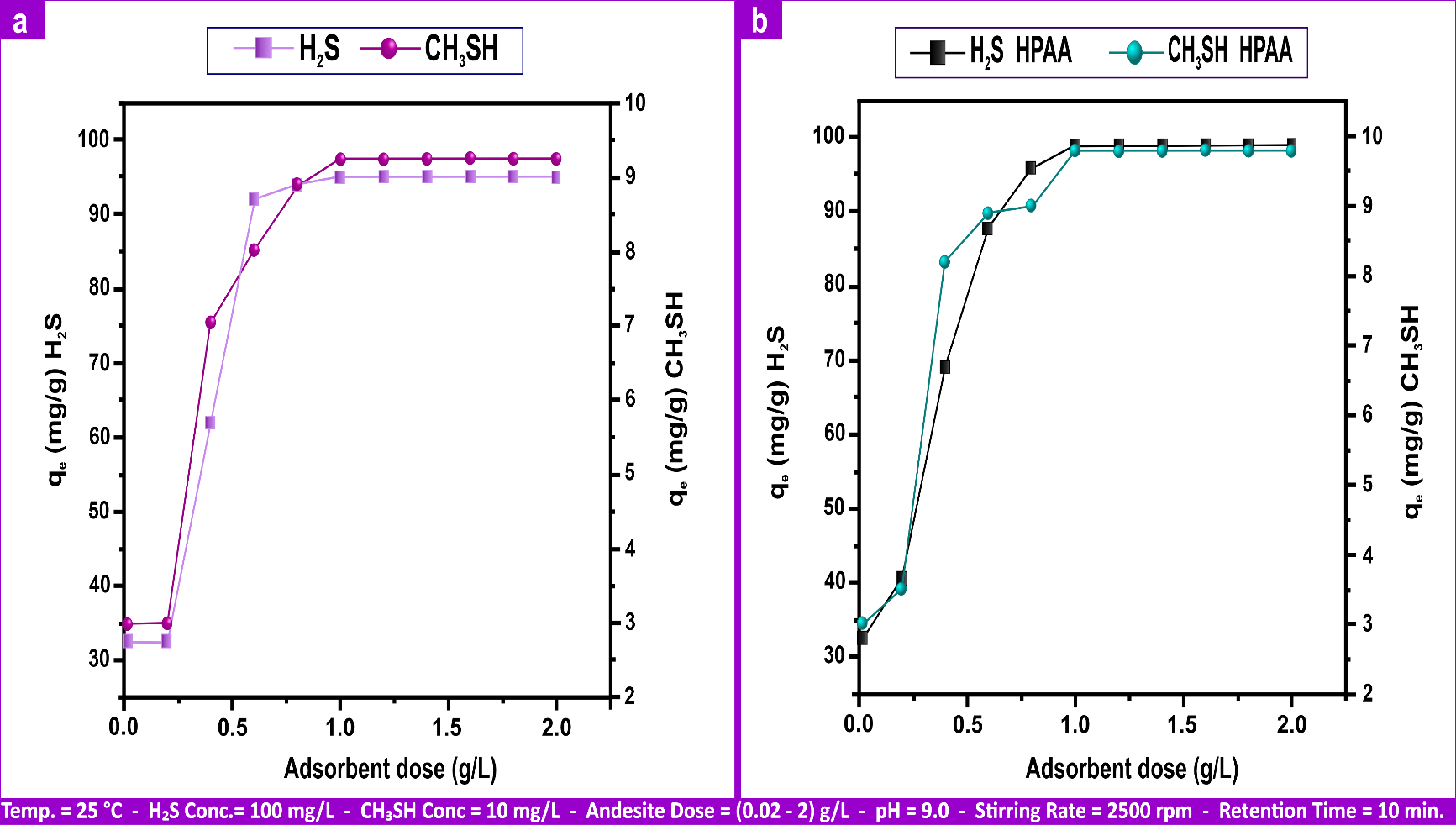
**Figure S5. (a)** Various adsorbent doses influence H_2_S and CH_3_SH adsorption on andesite and **(b)** HPAA composite.


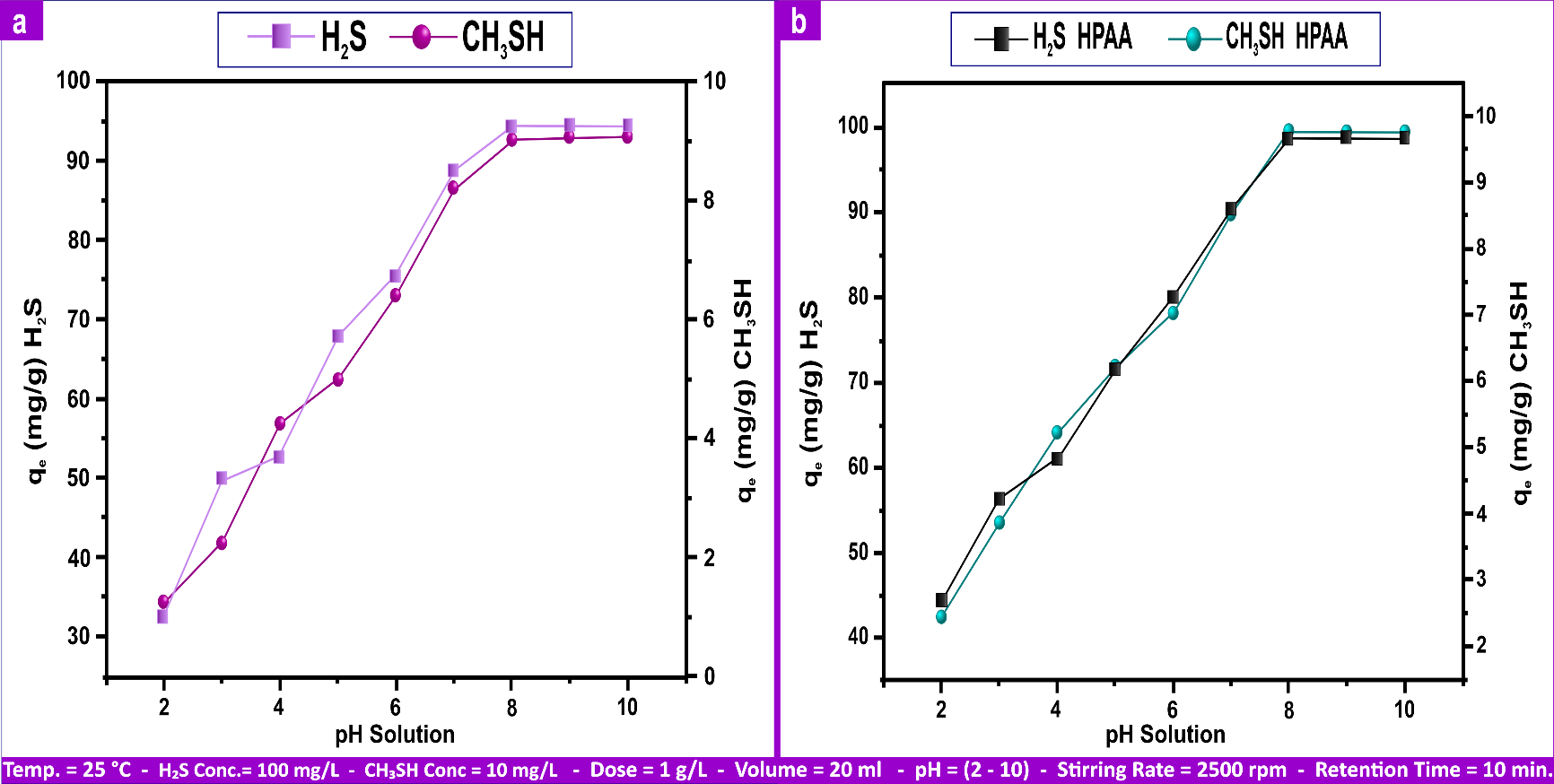


**Figure S6. (a)** pH influence on H_2_S and CH_3_SH adsorption on andesite and **(b)** HPAA composite.


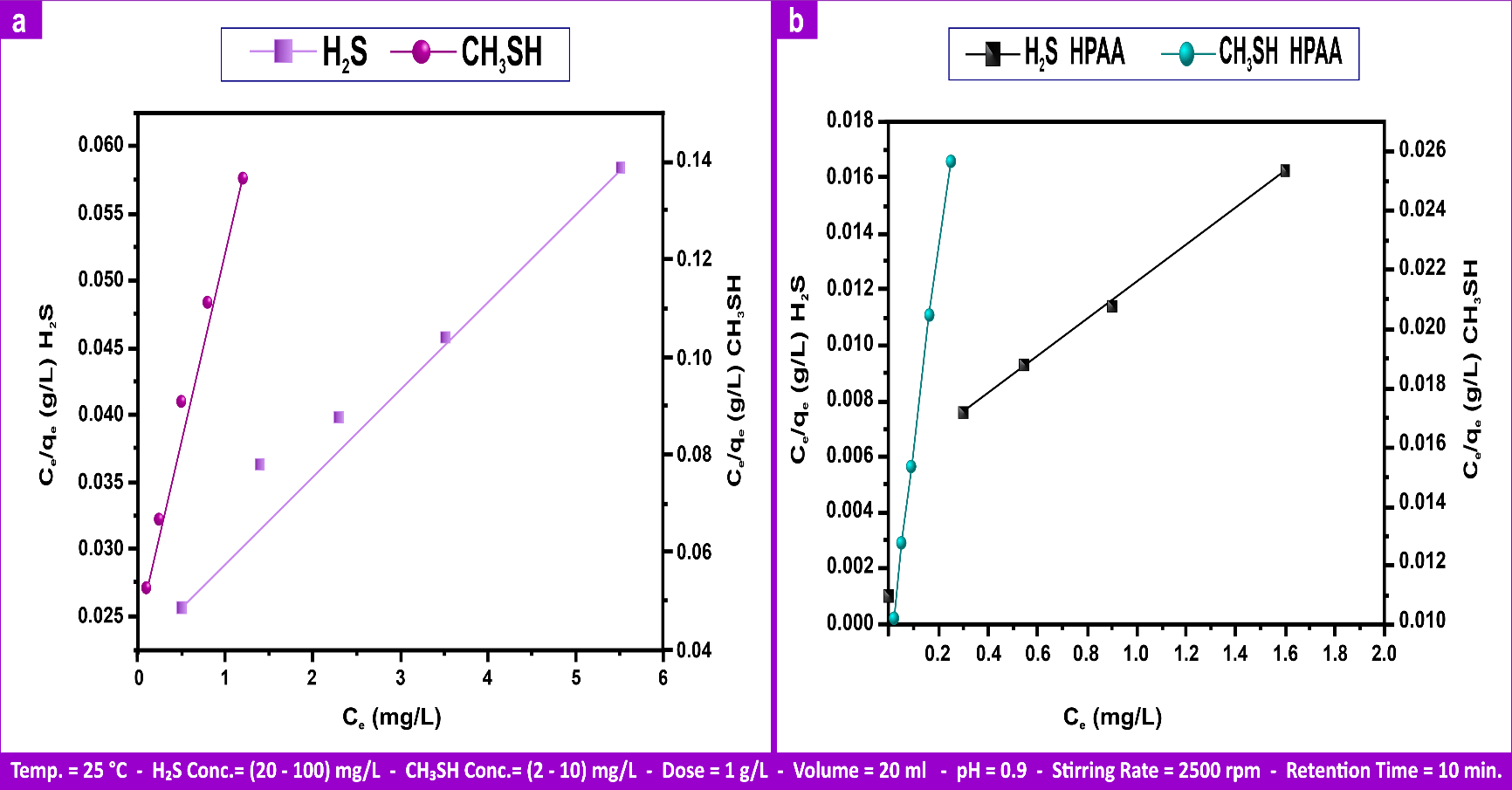

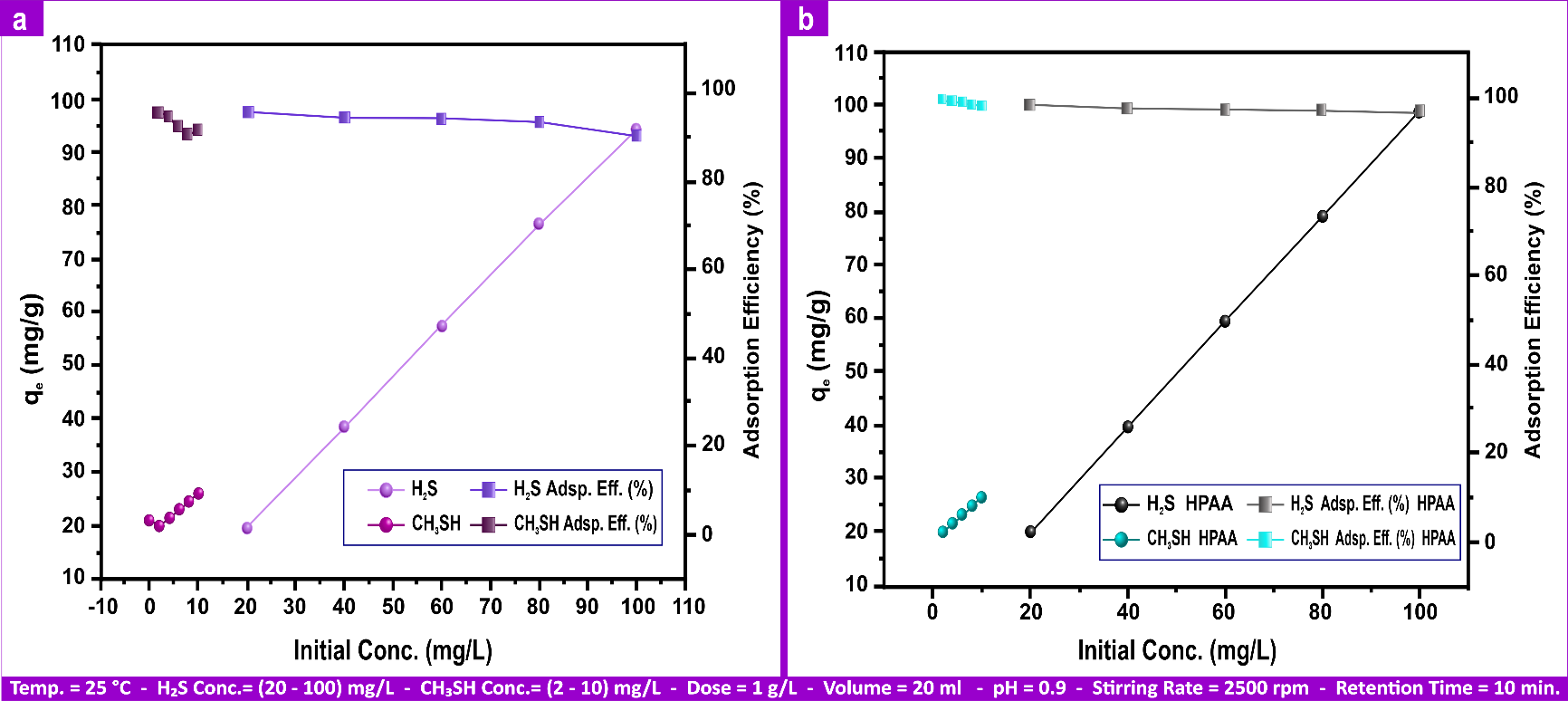
**Figure S7.** **(a)** Metal ion concentration influence on H_2_S and CH_3_SH adsorption on andesite and **(b)** HPAA composite

**Figure S8. (a)** H_2_S and CH_3_SH Langmuir isotherms on andesite and **(b)** HPAA composite range from (20-100) mg L⁻^1^ and (2-10) mg L⁻^1^.


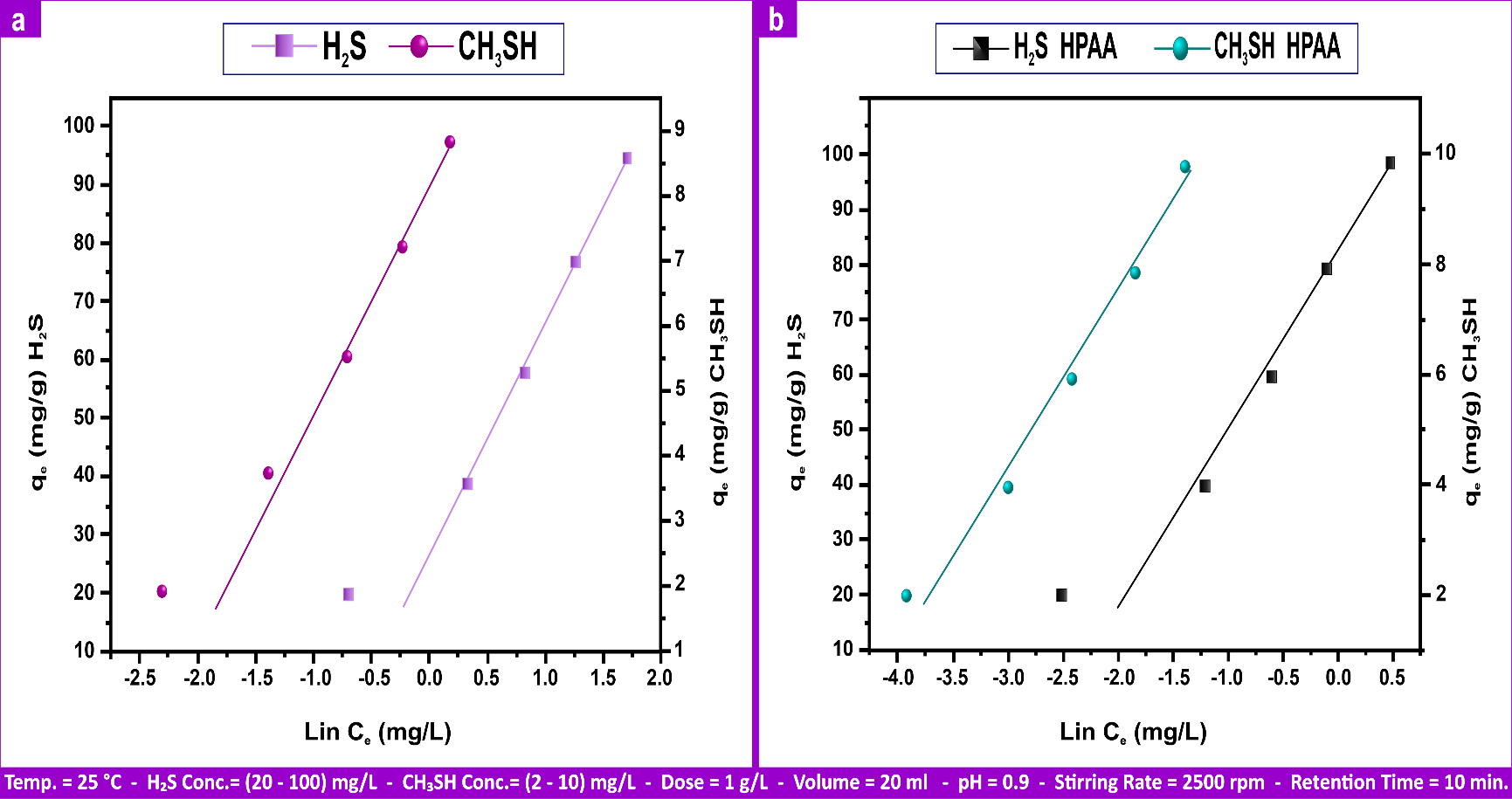

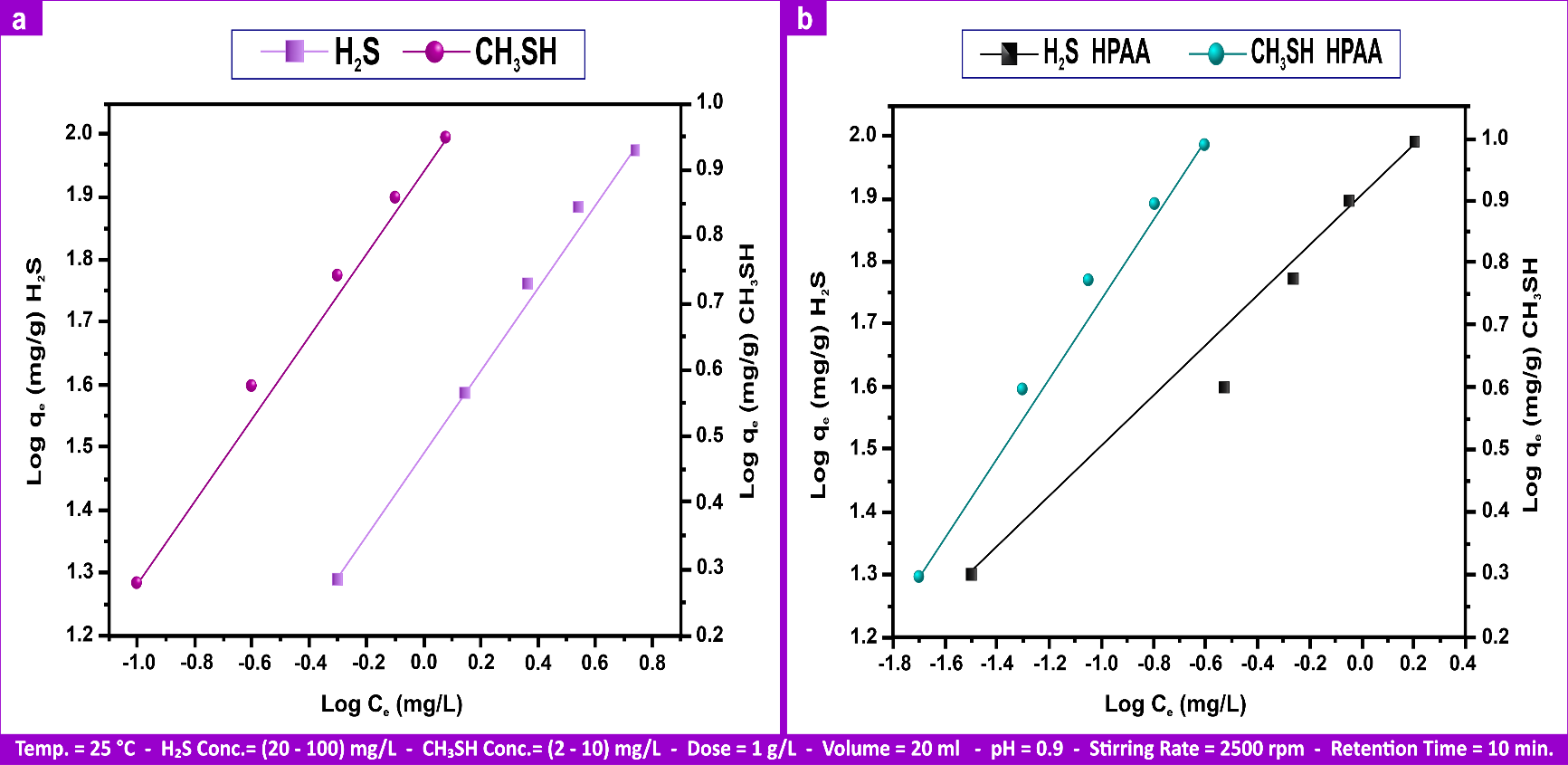
**Figure S9. (a)** H_2_S and CH_3_SH Freundlich isotherms on andesite and **(b)** HPAA composite range from (20-100) and (2-10) mg L⁻^1^.

**Figure S10.** **(a)** H_2_S and CH_3_SH Temkin isotherms on andesite and **(b)** HPAA composite range from (20-100) mg L⁻^1^ and (2-10) mg L⁻^1^.

|  | | **AL(III)**  **(mg L**⁻^1^**)** | | | |  | **Fe(III)**  **(mg L**⁻^1^**)** | | | |  | **H_2_S (mg L**⁻^1^**)** |  | **CH_3_SH (mg L**⁻^1^**)** |
| --- | --- | --- | --- | --- | --- | --- | --- | --- | --- | --- | --- | --- | --- | --- |
| **Isotherms** | **Parameters** | 0.1 | 0.2 | 0.4 | 0.5 |  | 0.1 | 0.2 | 0.4 | 0.5 |  | 100 |  | 10 |
| **Langmuir** | **q_m_ (mg g^-^**⁻^1^**)** | 4.08 | 7.77 | 15.45 | 18.68 |  | 4.03 | 7.66 | 15.32 | 18.41 |  | 111.11 |  | 14.83 |
|  | **K_L_ (L mg**⁻^1^**)** | 1.10 | 1.14 | 0.60 | 0.84 |  | 0.71 | 0.78 | 0.50 | 0.71 |  | 3.23 |  | 7.39 |
|  | **R^2^** | 0.997 | 0.998 | 0.995 | 0.996 |  | 0.994 | 0.997 | 0.993 | 0.996 |  | 0.951 |  | 0.996 |
| **Freundlich** | **K_F_ (mg g**⁻^1^**)** | 6.68 | 11.5 | 25.00 | 27.00 |  | 7.10 | 11.70 | 27.40 | 26.80 |  | 76.8 |  | 24.8 |
|  | **n** | 7.10 | 8.07 | 6.01 | 8.12 |  | 5.79 | 7.16 | 5.18 | 7.56 |  | 2.43 |  | 1.58 |
|  | **R^2^** | 0.955 | 0.801 | 0.848 | 0.838 |  | 0.940 | 0.841 | 0.839 | 0.803 |  | 0.974 |  | 0.989 |
| **Temkin** | **A_T_ (L mg**⁻^1^**)** | 0.14 | 0.26 | 0.60 | 0.61 |  | 0.16 | 0.27 | 0.64 | 0.62 |  | 0.02 |  | 0.08 |
|  | **b_T_ (kj mol**⁻^1^**)** | 6.57 | 3.64 | 1.62 | 1.59 |  | 6.04 | 3.56 | 1.51 | 1.56 |  | 0.09 |  | 0.80 |
|  | **R^2^** | 0.900 | 0.766 | 0.756 | 0.769 |  | 0.853 | 0.771 | 0.741 | 0.731 |  | 0.955 |  | 0.983 |


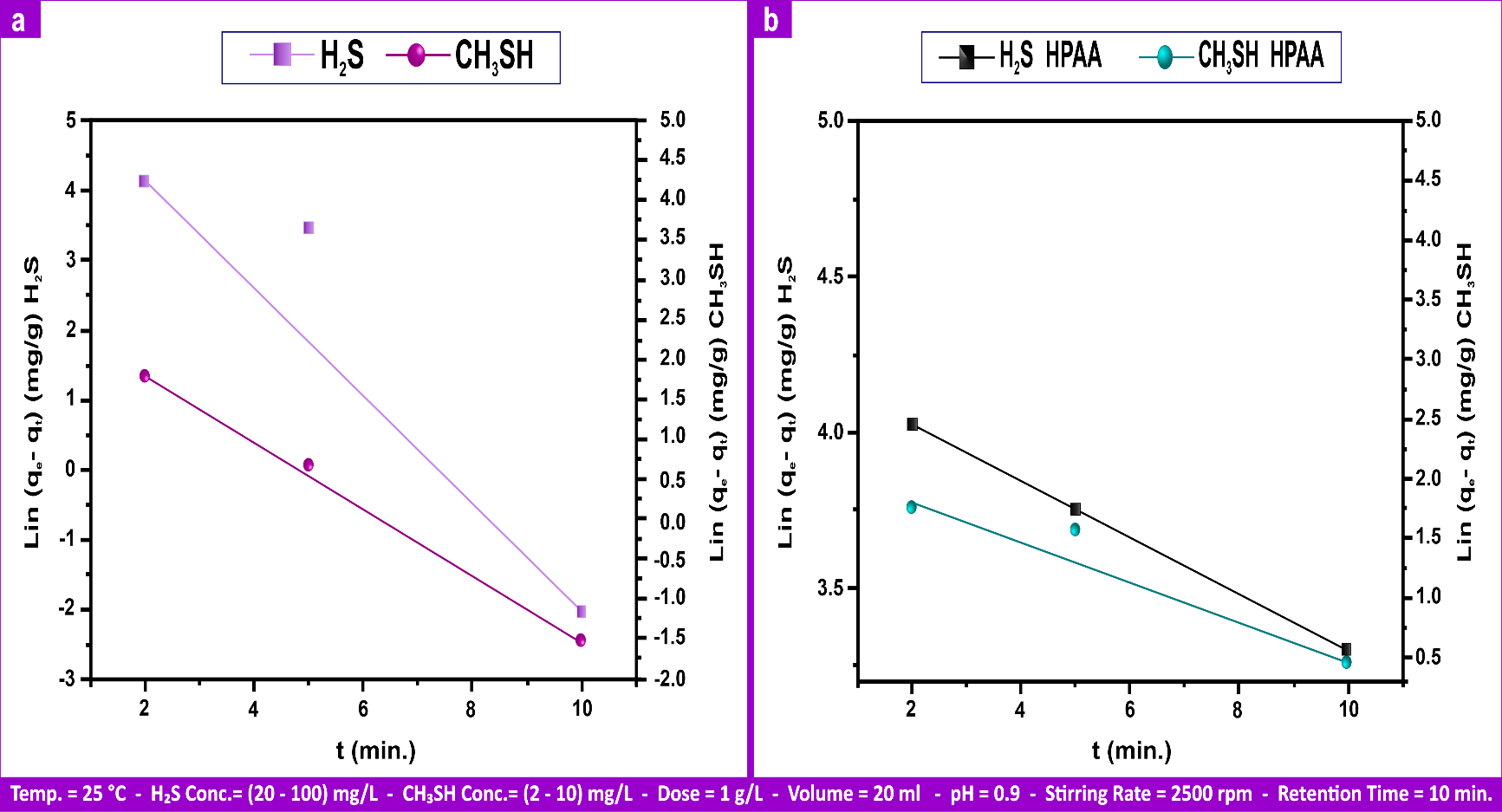
**Table S1.** Adsorption isotherm for Al(III), Fe(III), H_2_S, and CH_3_SH, HPAA composite´s Langmuir, Freundlich, and Temkin parameters.

**Figure S11. (a)** First-order kinetic curves for H₂S and CH₃SH adsorption on andesite and **(b)** HPAA composite range from (20-100) mg L⁻^1^ and (2-10) mg L⁻^1^.

**
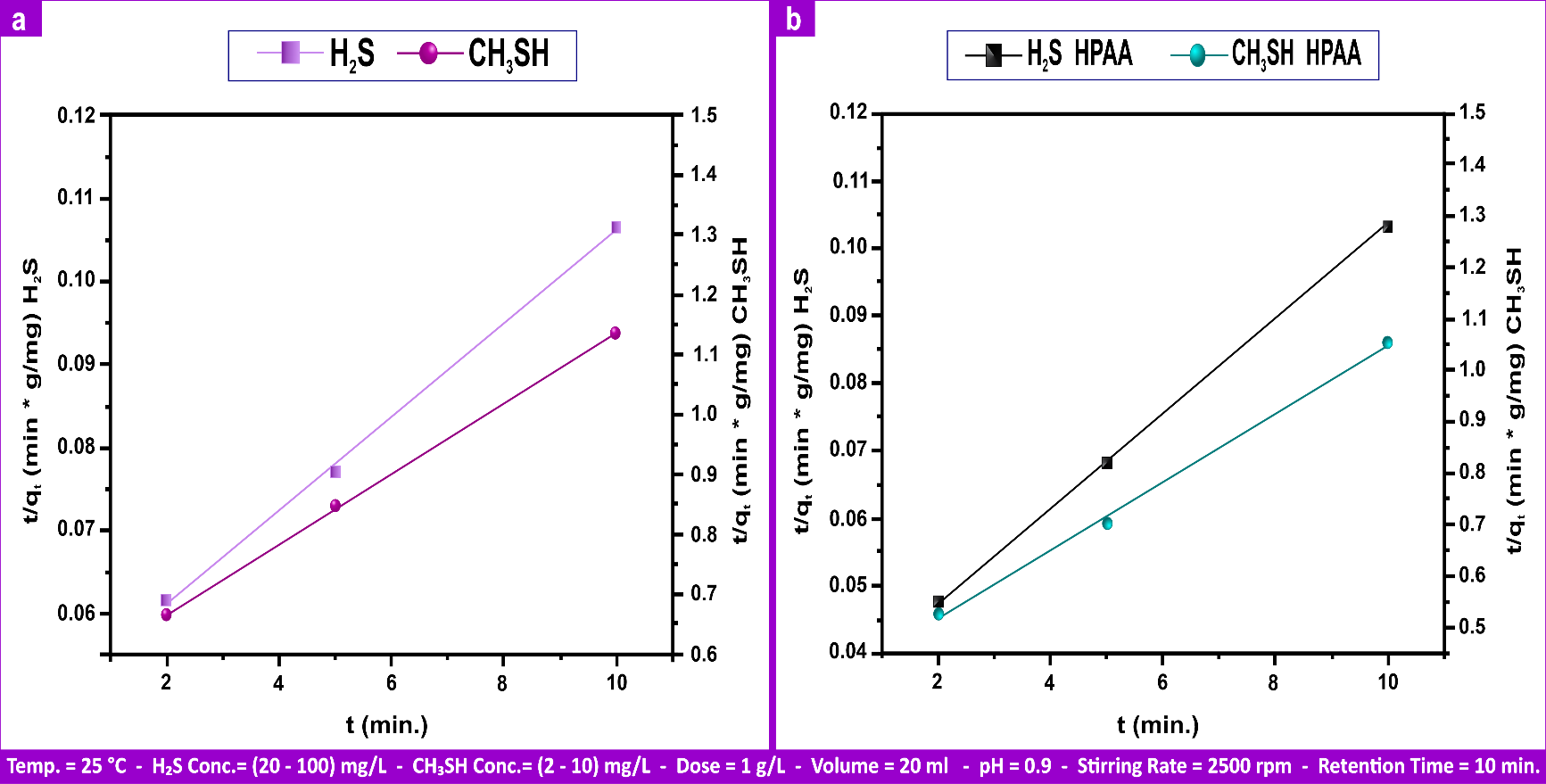
Figure S12. (a)** Second-order kinetic curves for H₂S and CH₃SH adsorption on andesite and **(b)** HPAA composite range from (20-100) mg L⁻^1^ and (2-10) mg L⁻^1^.

|  | | **AL(III)**  **(mg L⁻^1^)** | | | |  | **Fe(III)**  **(mg L⁻^1^)** | | | |  | **H_2_S**  **(mg L⁻^1^)** |  | **CH_3_SH (mg L⁻^1^)** |
| --- | --- | --- | --- | --- | --- | --- | --- | --- | --- | --- | --- | --- | --- | --- |
| **Kinetic model** | **Parameters** | 0.1 | 0.2 | 0.4 | 0.5 |  | 0.1 | 0.2 | 0.4 | 0.5 |  | 100 |  | 10 |
| **pseudo-first-order** | **q_e_ (mg g⁻^1^)** | 22.31 | 41.70 | 30.55 | 104.96 |  | 17.94 | 43.23 | 234.86 | 116.76 |  | 190.38 |  | 22.56 |
|  | **k_1_**  **(min⁻^1^)** | 0.28 | 0.29 | 0.36 | 0.28 |  | 0.24 | 0.27 | 0.36 | 0.28 |  | 0.51 |  | 0.52 |
|  | **R^2^** | 0.785 | 0.846 | 0.758 | 0.783 |  | 0.784 | 0.794 | 0.761 | 0.776 |  | 0.982 |  | 0.959 |
| **pseudo-second-order** | **q_e_ (mg g⁻^1^)** | 5.20 | 10.27 | 22.02 | 23.05 |  | 4.30 | 9.99 | 13.34 | 16.23 |  | 144.09 |  | 15.08 |
|  | **K_2_×10^-3^**  **(g mg⁻^1^ min⁻^1^)** | 0.02 | 0.009 | 0.002 | 0.004 |  | 0.02 | 0.008 | 0.009 | 0.01 |  | 0.001 |  | 0.01 |
|  | **R^2^** | 0.995 | 0.966 | 0.938 | 0.969 |  | 0.996 | 0.965 | 0.949 | 0.986 |  | 0.999 |  | 0.997 |

**Table S2**. Adsorption rate constant for first-order and second-order on HPAA composite as an adsorbent range from (0.1- 0.50) mg L⁻^1^ for Al(III) and Fe(III), (20-100) mg L⁻^1^ and (2-10) mg L⁻^1^ f or H_2_S and CH_3_SH.

|  |  | **AL(III)**  **(mg L⁻^1^)** |  | **Fe(III)**  **(mg L⁻^1^)** |  | **H_2_S**  **(mg L⁻^1^)** |  | **CH_3_SH (mg L⁻^1^)** |
| --- | --- | --- | --- | --- | --- | --- | --- | --- |
| **Kinetic models** | **Parameters** | 0.5 |  | 0.5 |  | 100 |  | 10 |
| **Intra-particle diffusion** | **K_pi_**  **(mg g⁻^1^min⁻^1/2^)** | 1.13 |  | 1.39 |  | 17.05 |  | 1.85 |
|  | **C (mg g⁻^1^)** | 8.03 |  | 7.06 |  | 24.33 |  | 8.03 |
|  | **R^2^** | 0.746 |  | 0.749 |  | 0.809 |  | 0.811 |


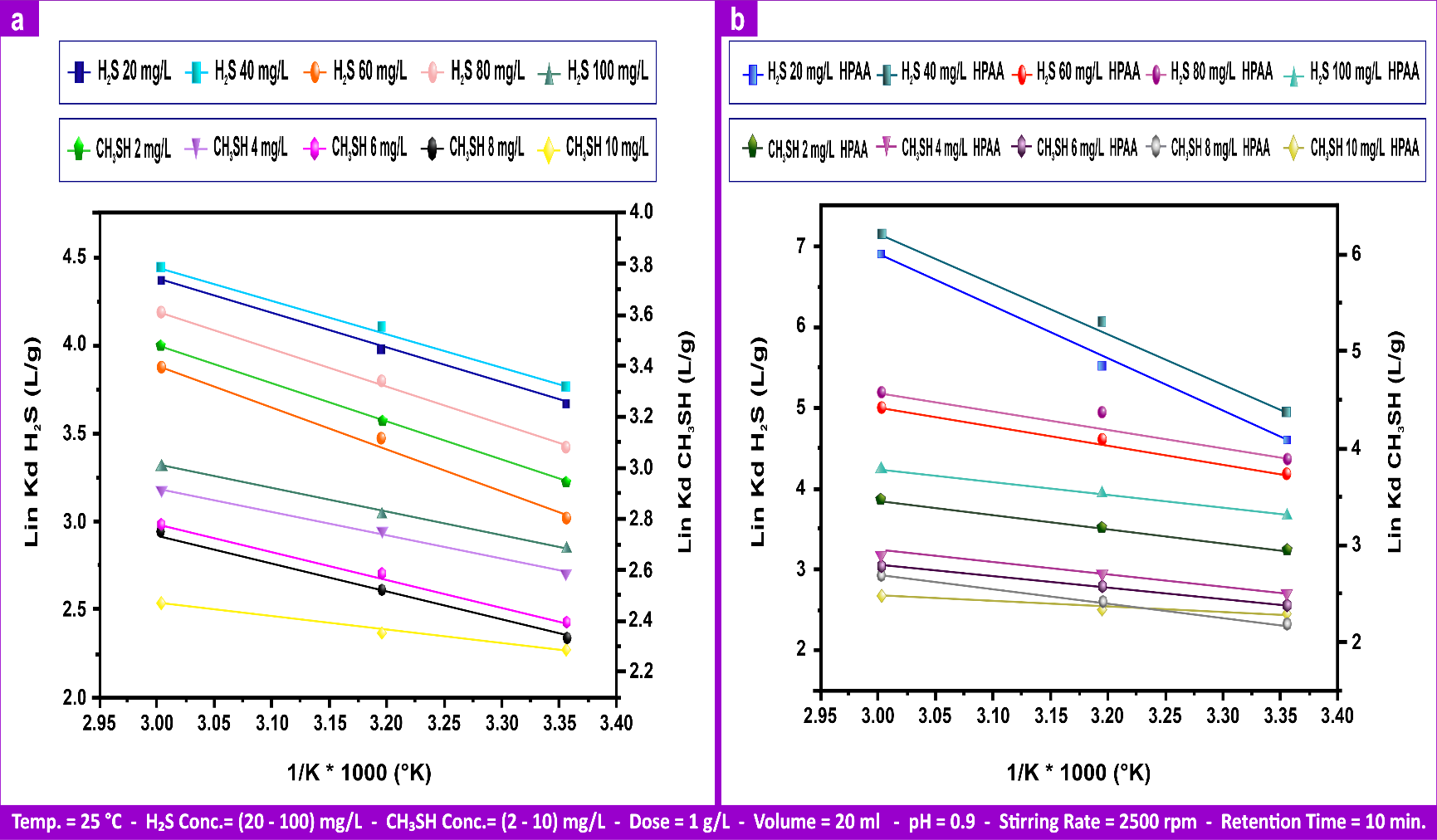
**Table S3.** Intra-particle diffusion model values for Al(III), Fe(III), H_2_S, and CH_3_SH adsorption on HPAA composite as an absorbent.

**Figure S13.** **(a)** Effect of temperature on the sorption of H_2_S and CH_3_SH on andesite; and **(b)** the thermodynamic behavior of the HPAA composite.

|  | **ΔH (kJ mol⁻^1^)** |  | **ΔS (J/k/mol)** |  | **E_a_**  **(J/k/mol)**  **(298-333)K** |  | **- ΔG (kJ mol⁻^1^)** | | |
| --- | --- | --- | --- | --- | --- | --- | --- | --- | --- |
|  |  |  |  |  |  |  | **298** | **313** | **323** |
| **Al(III (mg L⁻^1^)** |  |  |  |  |  |  |  |  |  |
| **0.1** | 30.63 |  | 232.51 |  | 33.11 |  | 69.26 | 72.74 | 77.39 |
| **0.2** | 9.52 |  | 151.98 |  | 11.99 |  | 47.56 | 47.56 | 50.59 |
| **0.4** | 11.62 |  | 156.56 |  | 14.09 |  | 46.64 | 48.99 | 52.12 |
| **0.5** | 9.87 |  | 83.03 |  | 12.35 |  | 24.73 | 25.97 | 27.64 |
| **Fe(III) (mg L⁻^1^)** |  |  |  |  |  |  |  |  |  |
| **0.1** | 20.57 |  | 195.93 |  | 23.04 |  | 58.36 | 61.30 | 65.22 |
| **0.2** | 8.31 |  | 145.00 |  | 10.78 |  | 43.20 | 45.37 | 48.27 |
| **0.4** | 8.60 |  | 143.99 |  | 11.08 |  | 42.90 | 45.06 | 47.94 |
| **0.5** | 8.84 |  | 77.86 |  | 11.32 |  | 23.19 | 24.36 | 25.92 |
| **H_2_S (mg L⁻^1^)** |  |  |  |  |  |  |  |  |  |
| **20** | 32.61 |  | 167.04 |  | 35.08 |  | 49.74 | 52.25 | 55.59 |
| **40** | 21.72 |  | 113.47 |  | 24.19 |  | 33.79 | 35.49 | 37.76 |
| **60** | 10.83 |  | 75.06 |  | 13.30 |  | 22.35 | 23.48 | 24.98 |
| **80** | 9.65 |  | 69.59 |  | 12.12 |  | 20.72 | 21.77 | 23.16 |
| **100** | 8.12 |  | 61.46 |  | 10.60 |  | 18.30 | 19.22 | 20.45 |
| **CH_3_SH (mg L⁻^1^)** |  |  |  |  |  |  |  |  |  |
| **2** | 54.67 |  | 246.21 |  | 57.15 |  | 73.31 | 77.01 | 81.93 |
| **4** | 37.91 |  | 164.13 |  | 40.39 |  | 48.87 | 51.33 | 54.61 |
| **6** | 19.26 |  | 99.54 |  | 21.74 |  | 29.64 | 31.13 | 33.12 |
| **8** | 16.36 |  | 87.73 |  | 18.83 |  | 26.12 | 27.44 | 29.19 |
| **10** | 13.91 |  | 77.19 |  | 16.39 |  | 22.98 | 24.14 | 25.69 |

**Table S4.** Thermodynamic parameters for Al(III) and Fe(III) adsorption on HPAA composite adsorbent range from (0.1- 0.50) mg L⁻^1^, (20-100) mg L⁻^1^, and (2-10) mg L⁻^1^ for H_2_S and CH_3_SH.
